# Supplementary material for: Genkwanin glycosides are major active compounds in Phaleria nisidai extract mediating improved glucose homeostasis by stimulating glucose uptake into adipose tissues
Source: Nat Commun. 2025 Aug 16;16:7648. doi: 10.1038/s41467-025-62689-8 (PMC12357923; doi:10.1038/s41467-025-62689-8)
Supplement: Supplementary file 1 — Supplementary Information [file 41467_2025_62689_MOESM1_ESM.pdf]

Supplemental information

Genkwanin glycosides are major active compounds in *Phaleria nisidai* extract mediating improved glucose homeostasis by stimulating glucose uptake into adipose tissues

Carla Horvath[1$], Joëlle Houriet[2,3$], Alexandra Kellenberger[1$], Caroline Moser[1], Lucia Balazova[1,4], Miroslav Balaz[1,4,5], Hua Dong[6], Aron Horvath[7], Isabel Reinisch[1], Vissarion Efthymiou[1], Adriano Rutz[2,3], Laurence Marcourt[2,3], Christopher Kitalong[7], Bertrand Graz[3,8,9], Victor Yano[8], Emerson Ferreira Queiroz[2,3], Jean-Luc Wolfender[2,3] and Christian Wolfrum[1,10*]*

[1]*Institute of Food, Nutrition and Health, ETH Zurich, Schorenstr. 16, 8603 Schwerzenbach, Switzerland*

[2]*School of Pharmaceutical Sciences, University of Geneva, CMU, Rue Michel Servet 1, 1211 Geneva, Switzerland*

[3]*Institute of Pharmaceutical Sciences of Western Switzerland, University of Geneva, CMU, Rue Michel Servet 1, 1211 Geneva, Switzerland*

[4]*Biomedical Research Center, Slovak Academy of Sciences, Dubravska cesta 9, 845 05 Bratislava, Slovakia*

[5]*Department of Animal Physiology and Ethology, Faculty of Natural Sciences, Comenius University, Ilkovicova 6, 841 04 Bratislava, Slovakia*

[6]*Institute for Stem Cell Biology and Regenerative Medicine, Stanford University School of Medicine, Stanford, CA 94305, USA*

[7]*Institute of Biomechanics, ETH Zurich, Balgrist Campus, Switzerland*

[8]*Pacific Academic Institute for Research, 822 Ernguul Rd., Koror, Palau*

[9]*Community Health Association – Geneva, Ch. Des Montaneyres 4, 1443 Champvent, Switzerland*

[10]*Nanyang Technical University (NTU), 50 Nanyang Avenue, 639798 Singapore*

$These authors contributed equally.

*Corresponding authohr

christian.wolfrum@ntu.edu.sg
jean-luc.wolfender@unige.ch

Supplementary Fig. 1

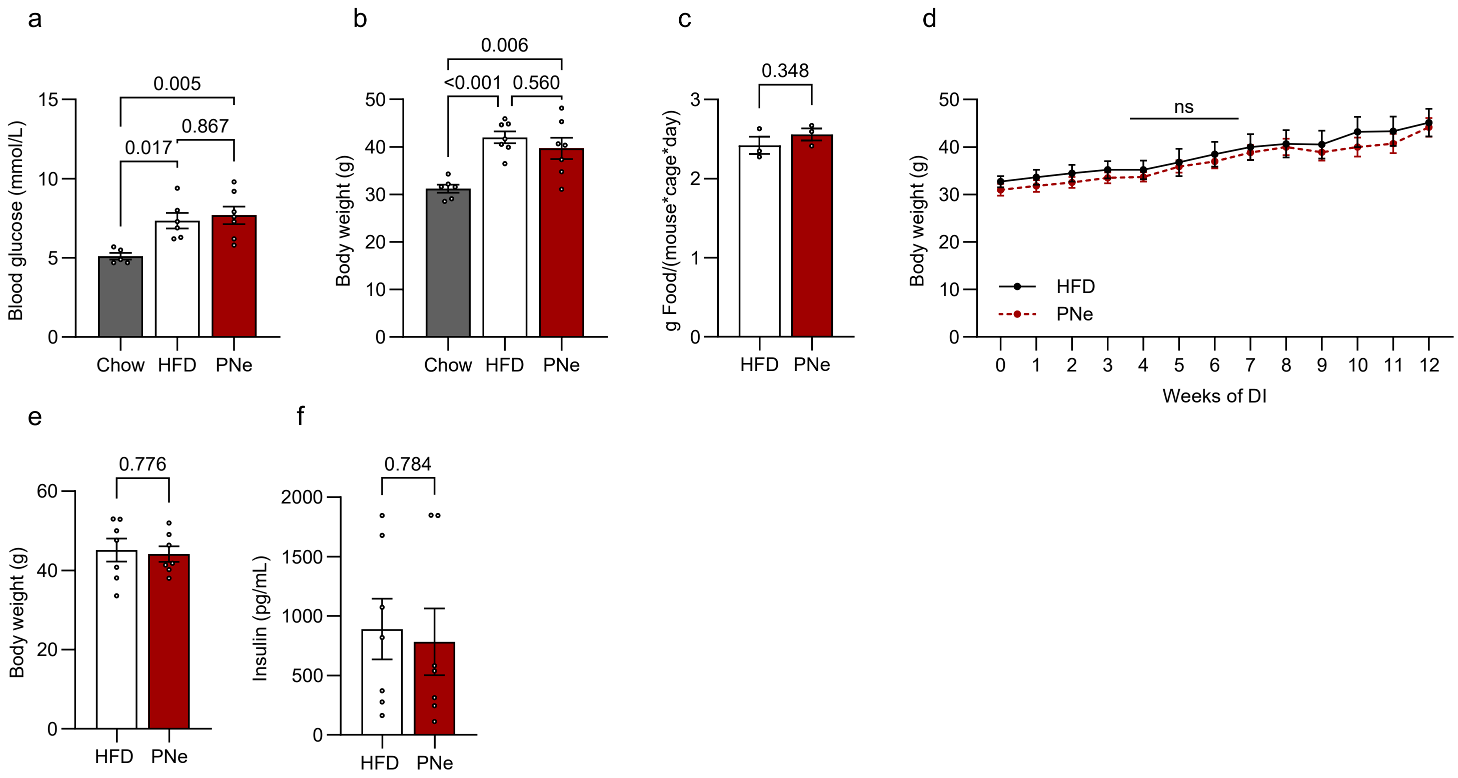

Supplementary Fig. 1 (related to Fig. 1): Validation of HFD model and food intake measurements. (a) Fasting blood glucose levels (chow n=5, HFD n=6, PNe n=7) and (b) body weight (chow n=6, HFD n=7, PNe n=7) after chow=17 weeks, HFD=17 weeks or DI=5 weeks for PNe. (c) Daily food intake calculated per mouse during the first week of the dietary intervention (n=3 cages per diet). (d-e) Body weight development and (e) final body weight (HFD n=7, PNe n=7) for study outlined in Fig. 1k. (f) Fasting insulin levels after HFD=6 weeks and DI=8 weeks (HFD n=7, PNe n=7). Data is presented as mean ± SEM. One-way ANOVA with Tukey´s post-hoc test between groups was applied in a, b. Student's t-test was applied in c, e, f. Repeated measures mixed effects analysis with Diet x Time interaction and Sidak´s post-hoc test for each time point was applied in d. Statistical test results are indicated as exact p-values with *p<0.05 considered significant. Source data are provided in Source Data 1. PNe, *Phaleria nisidai* extract; HFD, high fat diet.

Supplementary Fig. 2

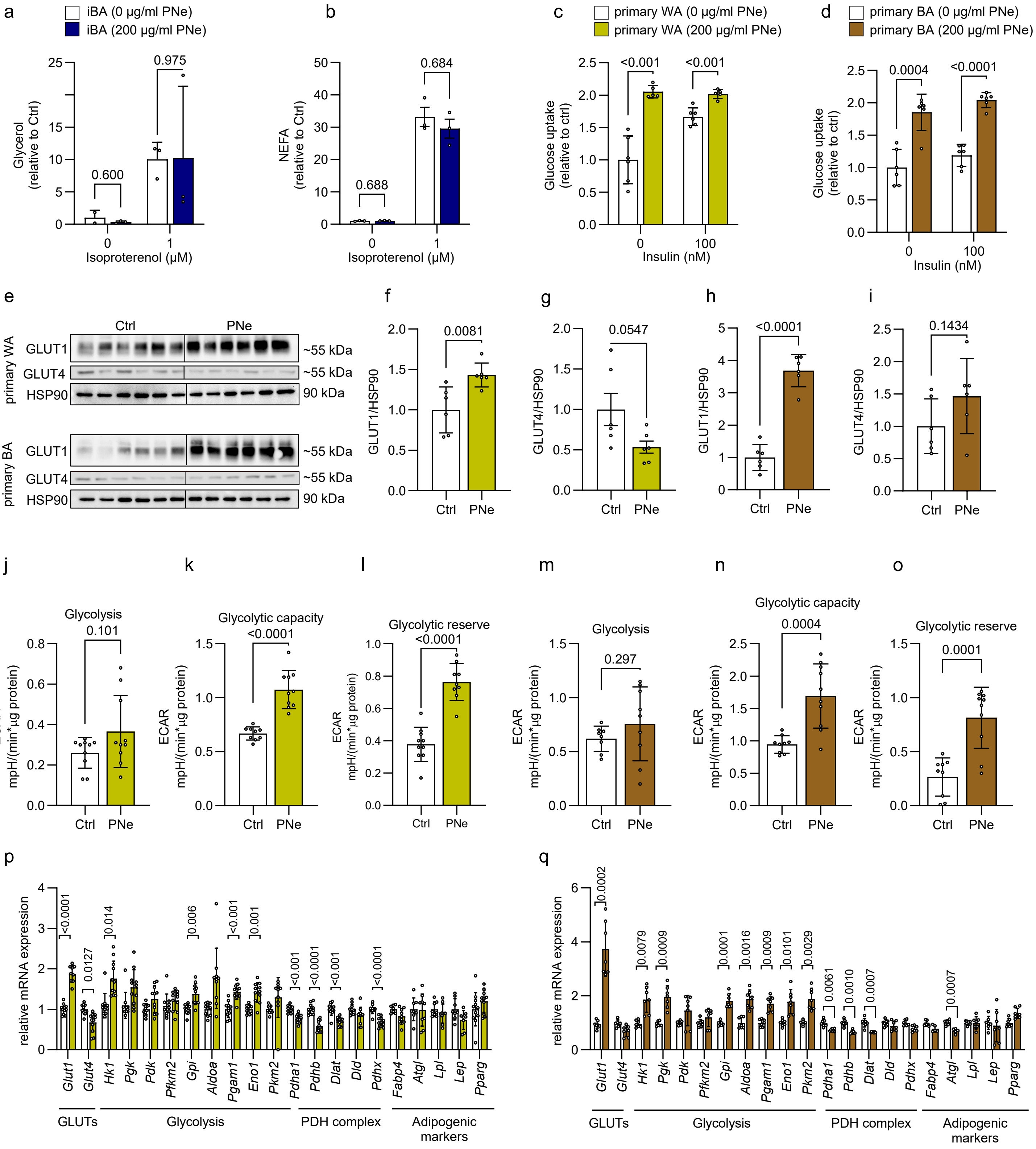

Supplementary Fig. 2 (related to Fig. 2): Validation of PNe effects in primary murine white and brownadipocytes. All readouts were performed after 3 days of PNe treatment (200 µg/mL). (a-b) Lipolysis measured as (a) glycerol (basal: 0 µg/mL n=2, 200 µg/mL n=3; stimulated: 0 µg/mL n=3, 200 µg/mL n=3) and (b) NEFA levels (0 µg/mL n=3 , 200 µg/mL n=3) in media from iBAs (±isoproterenol 2 hours). (c-d) Glucose uptake in primary murine (c) white (0 µg/mL n=6, 200 µg/mL n=5) and (d) brown adipocytes (0 µg/mL n=6, 200 µg/mL n=6). (e-i) GLUT1 and GLUT4 protein levels in primary murine (e) white (top panel) or brown (bottom panel) with (f-i) quantification (n=6/group). (j-o) Glycolytic stress test in primary murine white adipocytes (j) Glycolysis (0 µg/mL n=10, 200 µg/mL n=10); (i) Glycolytic capacity (0 µg/mL n=9, 200 µg/mL n=10) (l) Glycolytic reserve (0 µg/mL n=10, 200 µg/mL n=9) and (m-o) brown adipocytes (m) Glycolysis (0 µg/mL n=8, 200 µg/mL n=9), (n) Glycolytic capacity (0 µg/mL n=10, 200 µg/mL n=10), (o) Glycolytic reserve (0 µg/mL n=9, 200 µg/mL n=10). (p-q) Gene expression analysis of target genes regulating glucose metabolism and adipogenic markers in primary murine (p) white (*Glut1*: 0 µg/mL n=11, 200 µg/mL n=9; *Glut4*: n=11group; *Hk1*: 0µg/mL n=10, 200 µg/mL n=11; *Pgk*: 0µg/mL n=10,

200 µg/mL n=11; Pdk: n=11group; Pfkm2: 0µg/mL n=10, 200 µg/mL n=11; Pparg: n=11group; Gpi: n=11group; Aldoa: 0 µg/mL n=11, 200 µg/mL n=10; Pgam1: n=11group; Eno1: n=11group; Pkm2: 0 µg/mL n=11, 200 µg/mL n=10; Pdha1: n=11group; Pdhb: n=11group; *Dlat*: n=11group; *Dld:* n=11group; *Pdhx*: n=11group; *Fabp4*: 0 µg/mL n=8, 200 µg/mL n=9; *Atgl:* 0 µg/mL n=8, 200 µg/mL n=9; *Lpl*: 0 µg/mL n=8, 200 µg/mL n=9; *Lep*: n=8group) and (q) brown adipocytes. 0 µg/mL n=7, 200 µg/mL n=7 for all genes except: *Pdk*: 0 µg/mL n=6, 200 µg/mL n=7; *Atgl*: 0 µg/mL n=7, 200 µg/mL n=6. 1 independent experiment for a-b, c-q pool of 2 independent experiments. Data as mean ±SD. Student´s t-test was performed in f, g, h, I, j, k, l, m, n, o. Multiple t-test's with Holm-Sidak´s post-hoc test was performed in a, b, c, d. Two-tailed student´s t-test with Sidak's multiple comparison correction was applied in p, q. Statistical test results are indicated as exact p-values with *p<0.05 considered significant. Source data are provided in Source Data 1. PNe, *Phaleria nisidai* extract; Ctrl, control; GLUT, glucose transporter; iBA, immortalized brown adipocyte; WA, white adipocyte; NEFA, non-esterified fatty acids; ECAR, extracellular acidification rate.

Supplementary Fig. 3

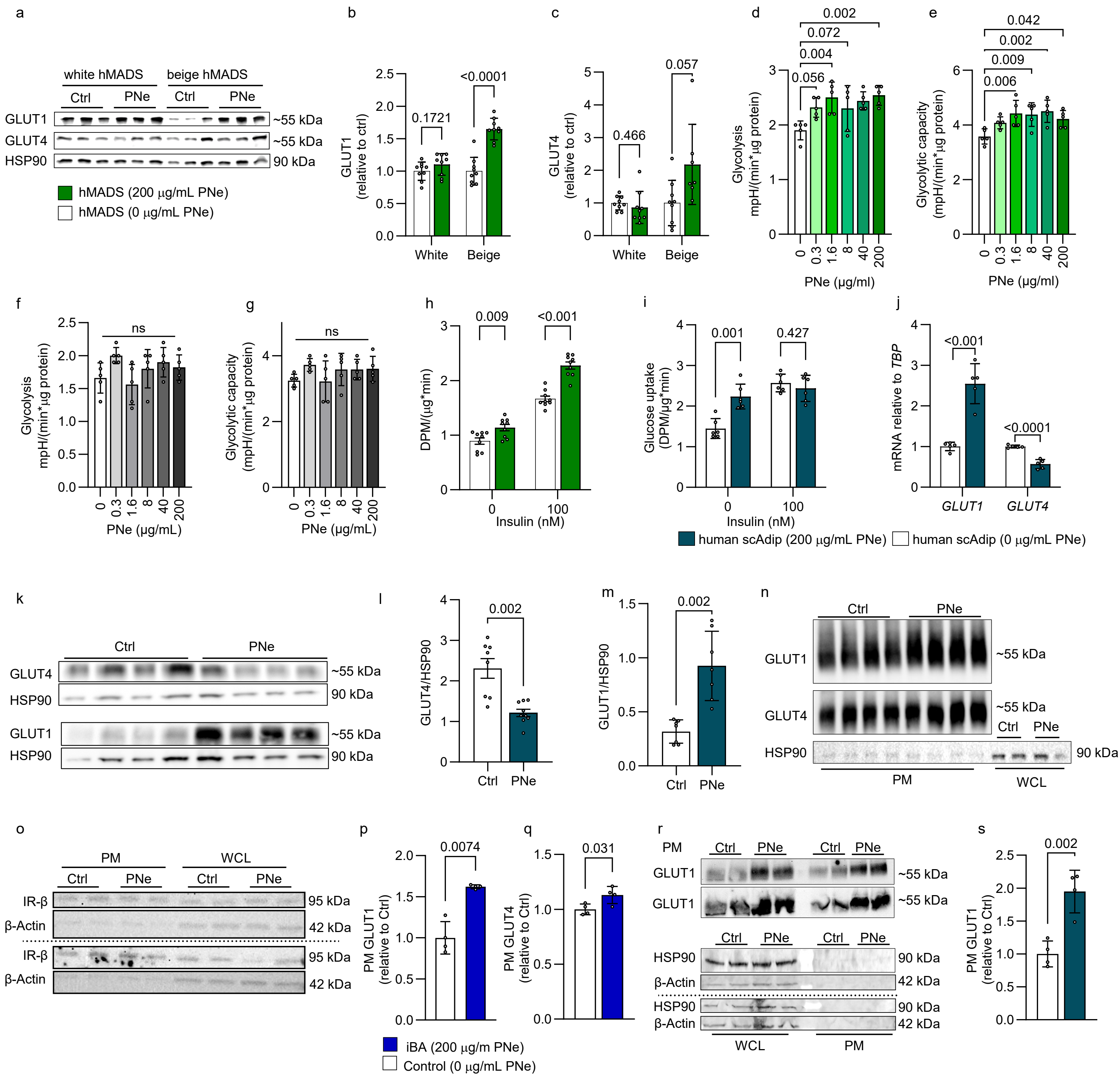

Supplementary Fig. 3 (related to Fig. 2): PNe enhances glucose metabolism in human adipocyte models by increasing GLUT1 plasma membrane levels. In all experiments, cells were treated with PNe for 3 days at the indicated concentrations. (a-c) Western blots of glucose transporter proteins in human beige and white hMADS and quantification of (b) GLUT1 (0 µg/mL n=9, 200 µg/mL n=9) and (c) GLUT4 (0 µg/mL n=9, 200 µg/mL n=7 for beige hMADS, n=9 for white hMADS). (d-g) Glycolytic flux in (d-e) beige (n=5 /condition) and (f-g) white (n=5 /condition) hMADS. (h) Glucose uptake in beige hMADS (0 µg/mL n=9, 200 µg/mL n=9). (i) Glucose uptake in primary human scAdip (0 µg/mL n=6, 200 µg/mL n=6). (j) Glucose transporter expression in human scAdip (0 µg/mL n=5, 200 µg/mL n=5). (k-m) GLUT4 (0 µg/mL n=8, 200 µg/mL n=9) and GLUT1 (0 µg/mL n=7, 200 µg/mL n=7) protein levels in human primary scAdip. (n-s) Subcellular fractionation showing (n-q) GLUT1 and GLUT4 (n=4 /condition) plasma membrane protein levels in iBAs and (r-s) GLUT1 (n=4/condition) PM levels in primary s.c. white adipocytes. 2 independent experiments for all graphs. Data are presented as mean ± SD. Two-tailed student's t-test was applied in l, m, p, q, s. Multiple t-test's with Holm-Sidak's post-hoc test was applied in b, c, h, i. Two-tailed student's t-test with Sidak multiple comparison adjustment was applied in j. ANOVA with Dunnett's post-hoc tests were applied in d, e, f, g to compare PNe doses against 0 µg/mL. Statistical test results are indicated as exact p-values with *p<0.05 considered significant. Source data are provided in Source Data 1. PNe, *Phaleria nisidai extract*; hMADS, human mesenchymal adipose derived stem cells; DPM, decays per minute; PM, plasma membrane; WCL, whole-cell lysate; scAdip, subcutaneous adipocytes; iBA, immortalized brown adipocytes, IR-β, Insulin receptor beta.

Supplementary Fig. 4

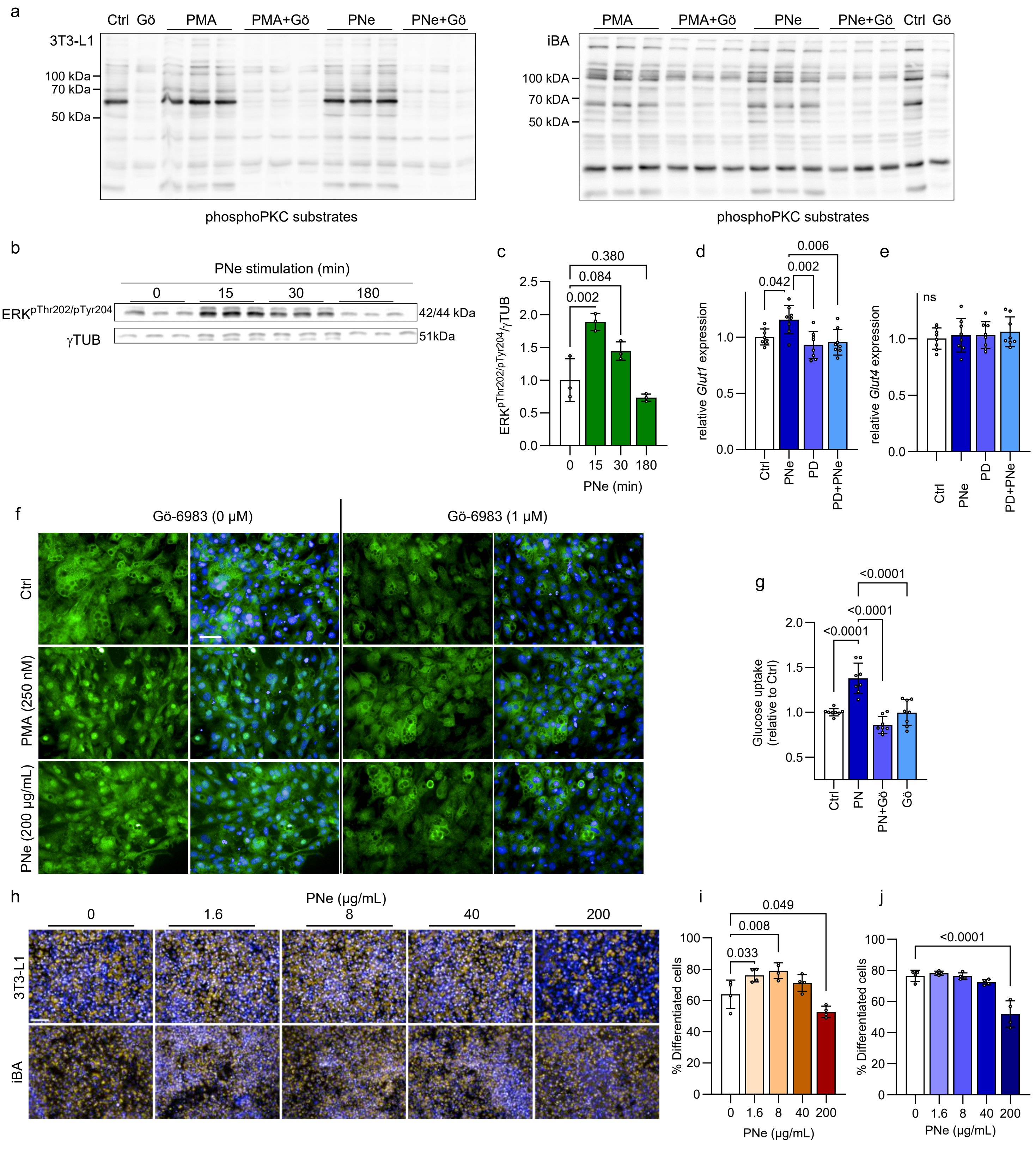

Supplementary Fig. 4 (related to Fig. 3): Acute effect of PNe on adipocytes is mediated via PKC-ERK1/2 signaling. (a) Effect of PKC-inhibitor Gö-6983 on phosphoPKC substrates after PNe stimulation in 3T3-L1 and iBAs (Ctrl n=1, Gö n=1, PMA n=3, PMA+Gö n=3, PNe n=3, PNe+Gö n=3). (b-c) Time course of PNe-induced ERK1/2 phosphorylation at Thr202 and Tyr204 in beige hMADS (n=3 /condition). (d-e) Effect of ERK-inhibitior (PD184352, 0.5 µM) on *Glut1* and *Glut4* mRNA expression after 180 min of PNe exposure in iBAs (n=8 /condition). (f) Representative images of ERK1/2 (green) immunofluorescence staining in iBAs (180 min PNe or PMA treatment ± Gö-6983). Nuclei blue. 40x objective, scale bar 100 µM. (g) Glucose uptake assay in iBAs (80 min PNe, 30 min pretreatment with Gö-6983 (n=8 /condition). (h) Representative pictures of 3T3-L1 and iBAs with PNe treatment during differentiation. (i-j) Lipid-dropblet based quantification of adipocyte differentiation in (i) 3T3-L1 (n=4 / condition) and (i) iBAs (n=4 /condition). Scale bar 100 µM. 20 images/condition.

1 independent experiment for b, i, j. 2 independent experiments for d-g. Data are presented as mean ± SD. One-way ANOVA with Dunnett´s post-hoc test compared to Ctrl was applied in i, j. One-way ANOVA with Tukey's post-hoc test comparing all groups was applied in c, d, e, g. Statistical test results are indicated as exact p-values with *p<0.05 considered significant. Source dataare provided in Source Data 1. PNe, *Phaleria nisidai* extract; Gö, Gö-6983; iBAs, immortalized brown adipocytes; PMA, phorbol 12-myristate 13-acetate.

Supplementary Fig. 5

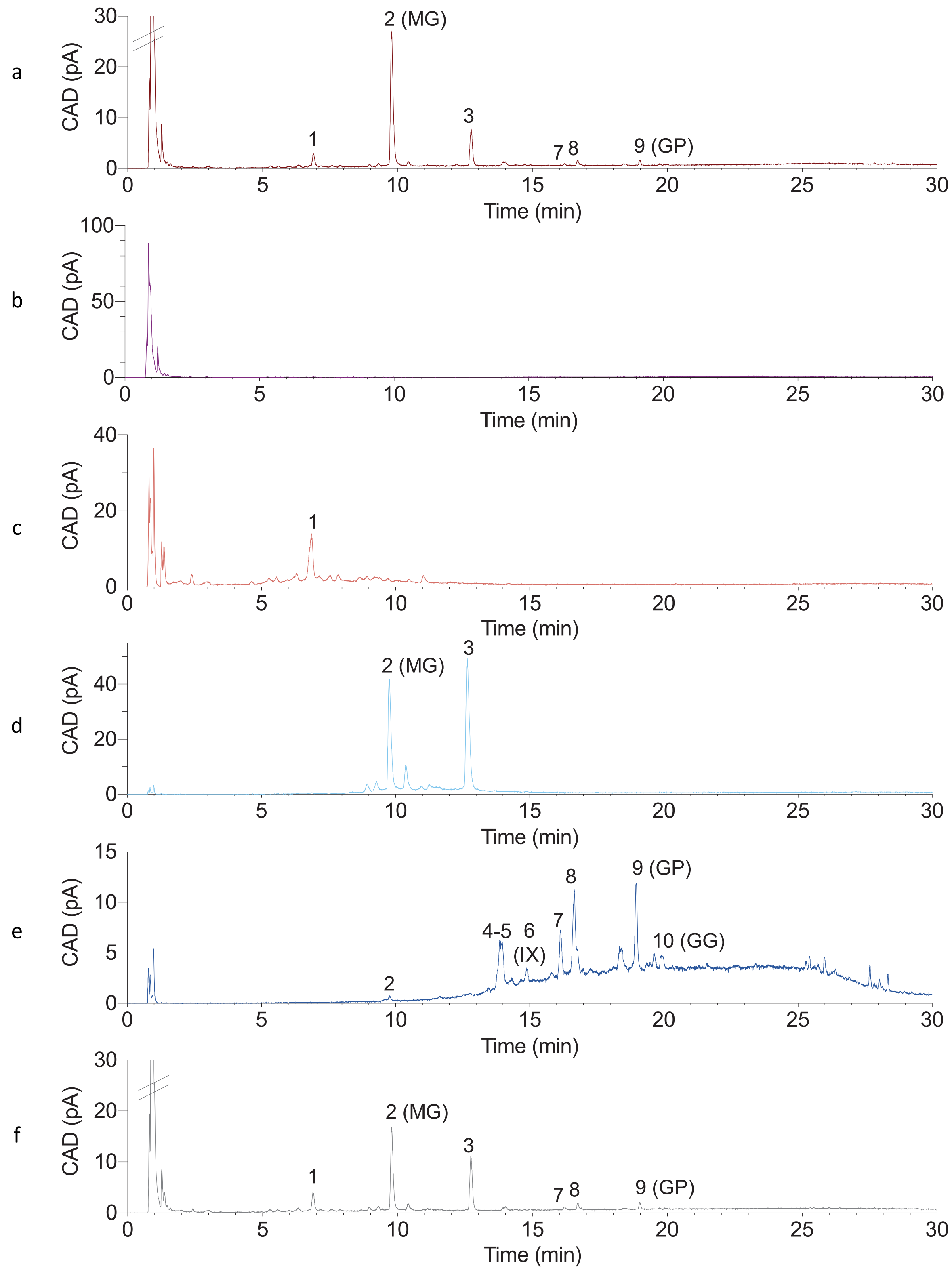

Supplementary Fig. 5 (related to Fig. 4 and 5): Semi-quantitative chemical profiling obtained by UHPLC-CAD. (a) PN extract, demonstrating the high proportion of 2. (b) Aqueous fraction (F1) rich in polar metabolites (saccharides). (c) Fraction F2 depleted in 2, grouping the compounds eluting before 2 in reverse phase chromatographic conditions. (d) Fraction F3 containing 2 and 3. (e) Fraction F4 depleted in 2, grouping the compounds eluting after 2. (f) Reconstituted extract prepared from the four fractions in proportion to the fractionation yields, demonstrating the similarity with (a). UHPLC-CAD, ultra-high pressure liquid chromatography - corona-charged aerosol detection; PN, *Phaleria nisidai*; 2, mangiferin (MG); 3, iriflophenone-2-*O*-α-rhamnoside. See method section “Description of the isolated compounds”. Source data are provided in Source Data 2.

Supplementary Fig. 6

a

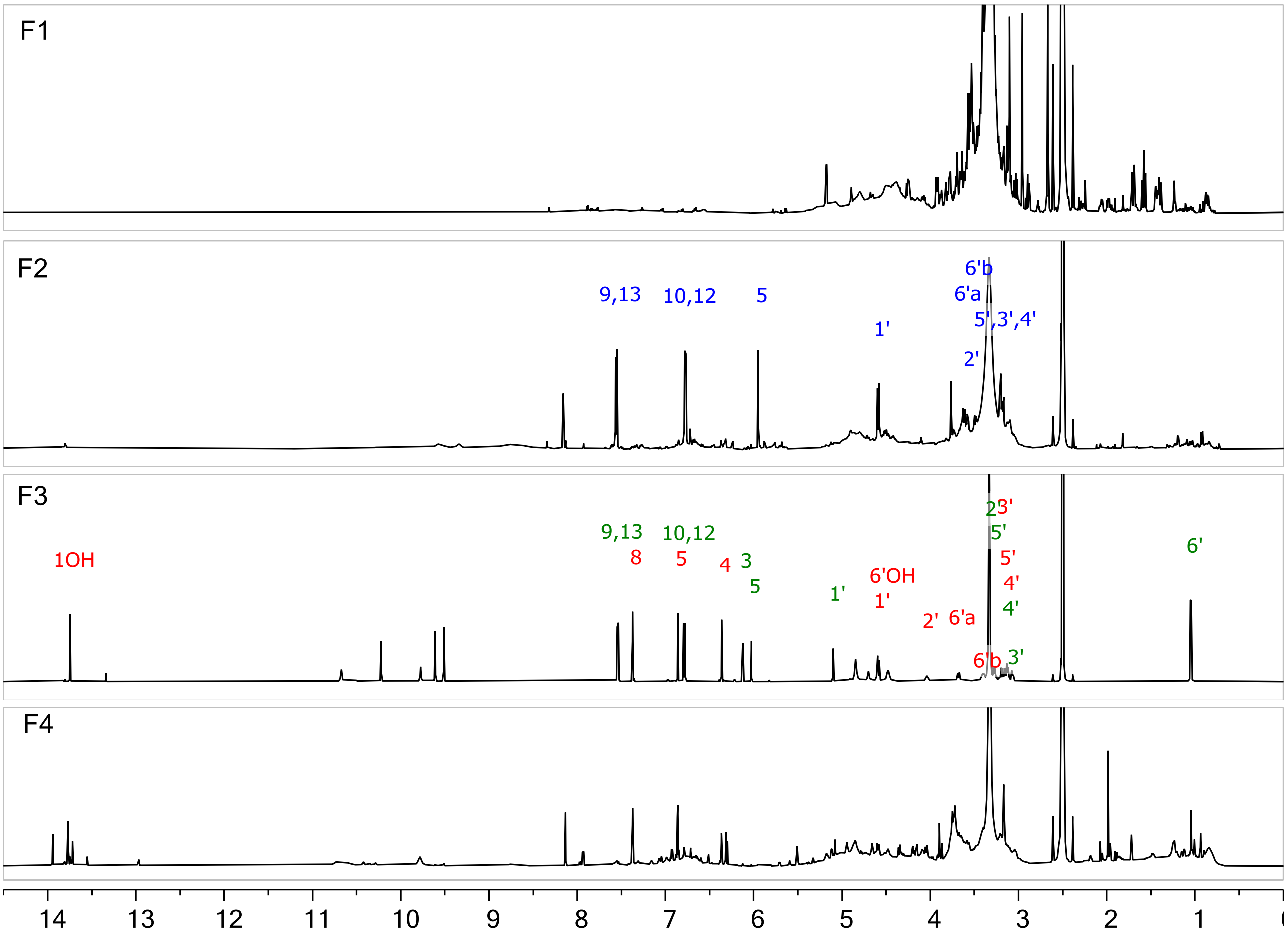

b

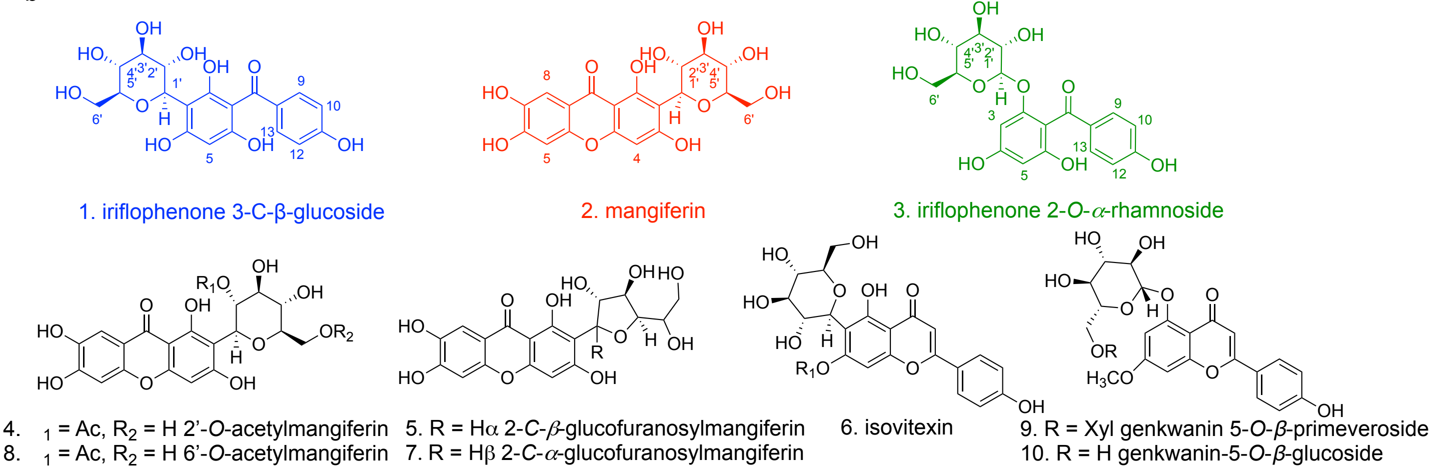

Supplementary Fig. 6 (related to Fig. 4 and 5): Complementary chemical composition of the PN fractions. (a) 1H-NMR spectra of the four fractions of PN extract demonstrating their complementary chemical composition. F1 contained polar constituents (mainly saccharides), F2 contained mainly constituent 1, F3 mainly constituents 2 and 3, and F4 constituents 4 to 10. F1, F2 and F4 were depleted in 2. (b) Chemical structures of the compounds identified by NMR. Source data are provided in Source data 2. PN, *Phaleria nisidai*.

Supplementary Fig. 7

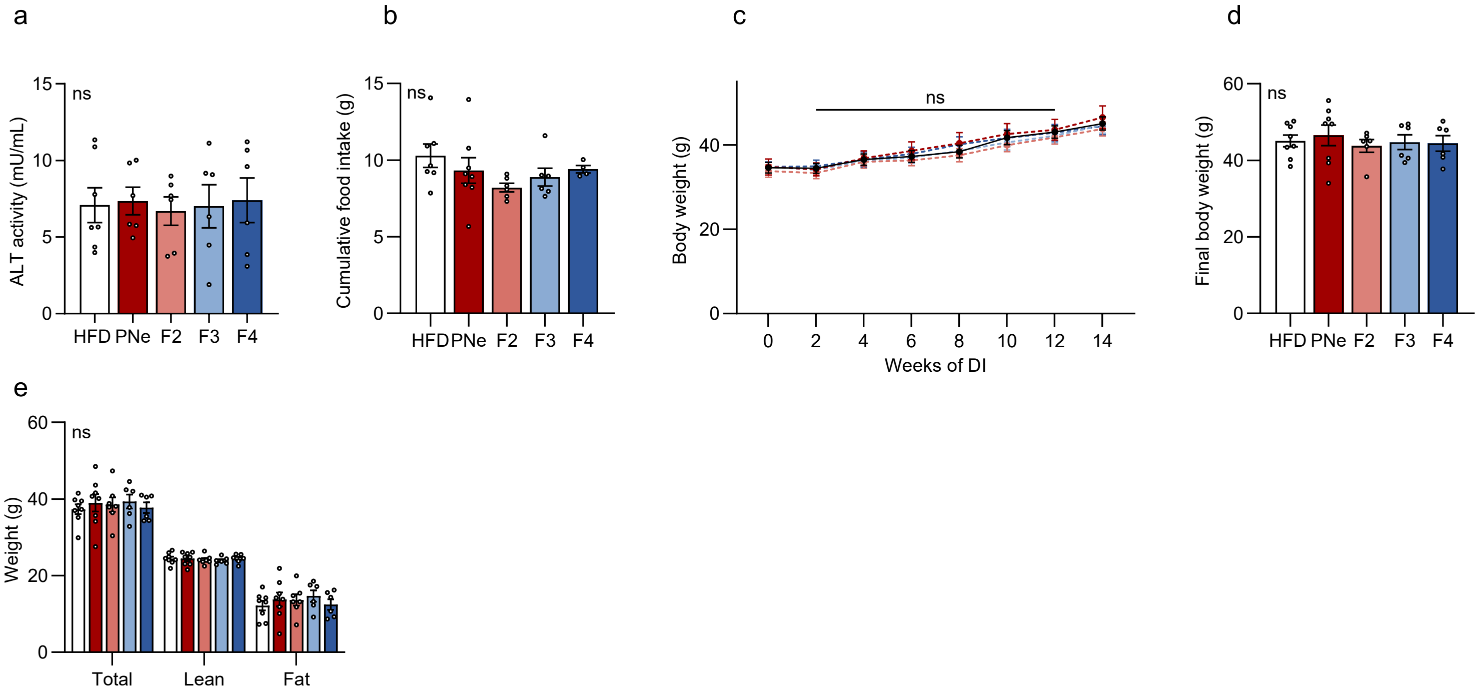

Supplementary Fig. 7 (related to Fig. 4): PNe and PNe fractions do not affect ALT levels or body weight. (a) Plasma ALT levels after 14 weeks of DI (HFD n=7, PNe n=6, F2 n=6, F3 n=6, F4 n=6). (b) Cumulative food intake after 6 weeks of DI (HFD n=7, PNe n=8, F2 n=6, F3 n=6, F4 n=4). (c) Body weight development and (d) terminal body weight (HFD n=8, PNe n=8, F2 n=6, F3 n=6, F4 n=6). (e) Body composition after 6 weeks of DI (HFD n=8, PNe n=8, F2 n=7, F3 n=6, F4 n=6). Data are presented as mean ± SEM. One-way ANOVA with Dunnett´s post hoc test against HFD control was applied in a, b, d. Repeated measures two-way ANOVA with a diet x time interaction and Sidak´s post-hoc test was applied in c to compare each treatment to HFD for each time point. Comparisons were grouped into a single family to control FWER, α=0.05. Two-way ANOVA with Sidak´s post hoc test against HFD control was applied in e. Comparisons were grouped into into one family to control FWER, α=0.05. Statistical test results are indicated as exact p-values with *p<0.05 considered significant. Source data are provided in Source Data 1. ALT, alanine transaminase; DI, dietary intervention; iWAT, inguinal white adipose tissue; eWAT, epididymal white adipose tissue; HFD, high fat diet; PNe, *Phaleria nisidai* extract; F2-4, fractions 2-4.

Supplementary Fig. 8

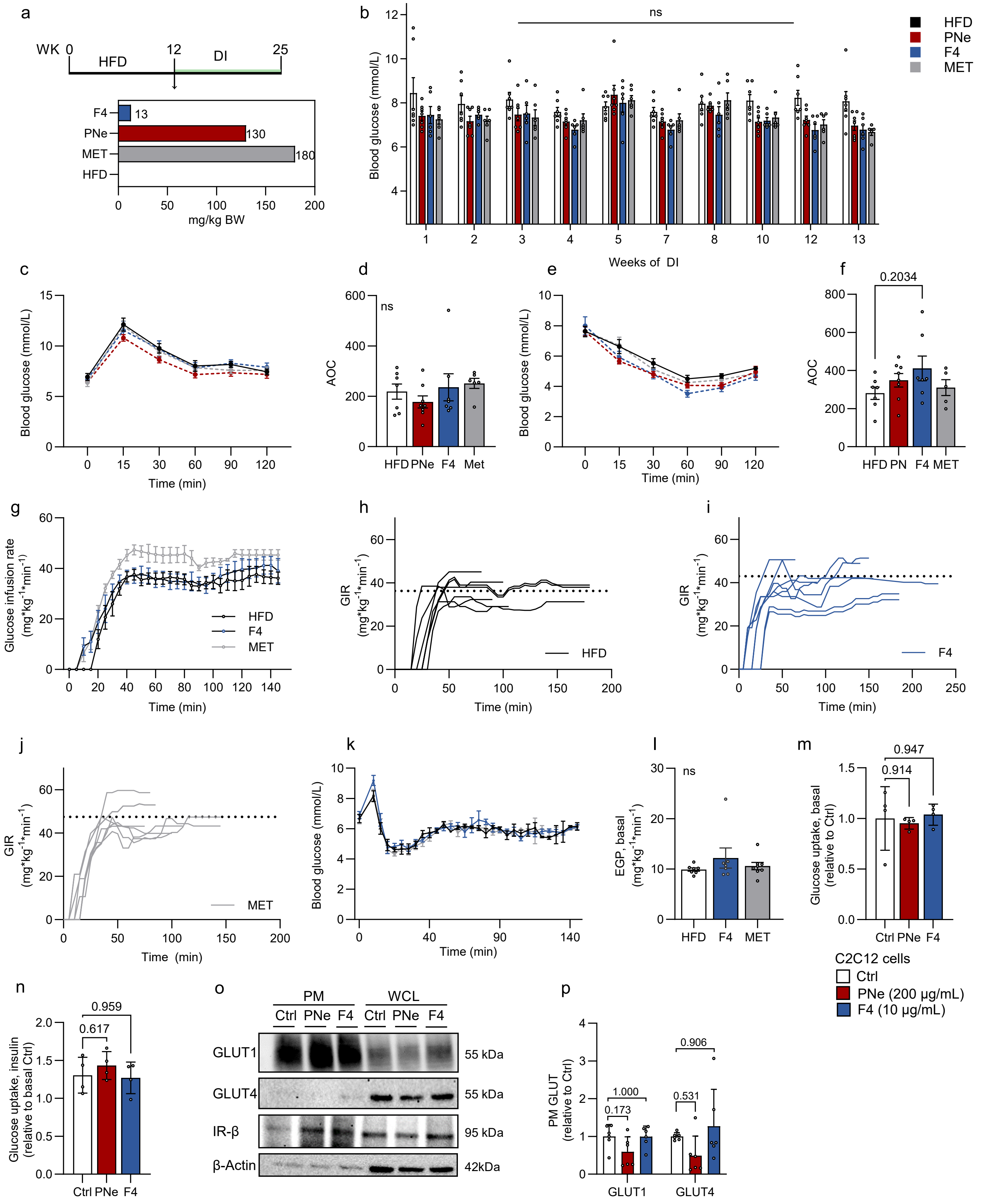

Supplementary Fig. 8 (related to Fig. 4): Effects of F4 and metformin on glucose homeostasis in DIO mice. (a) Experimental outline for panels b-f. (b) Random fed blood glucose (HFD n=7, PNe n=7, F4 n=6, MET n=6). (c-d) (c) Oral sucrose tolerance test (2 mg/g BW, DI=10 weeks) and (d) AOC (HFD n=7, PNe n=8, F4 n=7, MET n=6). (e-f) Insulin tolerance test (DI=9 weeks) and (f) AOC (HFD n=7, PNe n=8, F4 n=7, MET n=5). (g-j) GIR as (g) mean per condition until 145 min or individual mice for (h) HFD n=7, (i) F4 n=8 or (j) MET n=8. (k) Blood glucose during clamp procedure (HFD n=6, F4 n=8, MET n=8). (l) Basal endogenous glucose production (HDF n=7, F4 n=7, MET n=8). (m) Basal and (n) insulin-stimulated glucose uptake in C2C12 cells after 3 days of treatment (n=4/condition). 1 experiment.

(o-p) Plasma membrane GLUT1 and GLUT4 protein levels in C2C12 cells after 3 days of treatment (n=6/ condition). 2 independent experiments. Data are presented as mean ± SEM or mean ± SD for in vitro data. Repeated measures mixed-effects analysis with Diet x time interaction and Sidak's multiple comparison test was applied in b to compare all treatments for each time point. Comparisons were grouped into one family to control FWER , α=0.05. One-way ANOVA with uncorrected Fisher's LSD post-hoc test between all groups was applied in d, f, l. One-Way ANOVA with Dunnett´s post-hoc test was applied in m-n against Ctrl cells. Two-Way ANOVA with Dunnett's post-hoc test against Ctrl cells was applied with Sidak correction across all genes. Statistical test results are indicated as exact p-values with *$p<0.05$ considered significant. Source data are provided in Source Data 1. DI, dietary intervention; F4, fraction 4; PNe, *Phaleria nisidai* extract; HFD, high fat diet; GIR, glucose infusion rate, FWER, family-wise error rate.

Supplementary Fig. 9

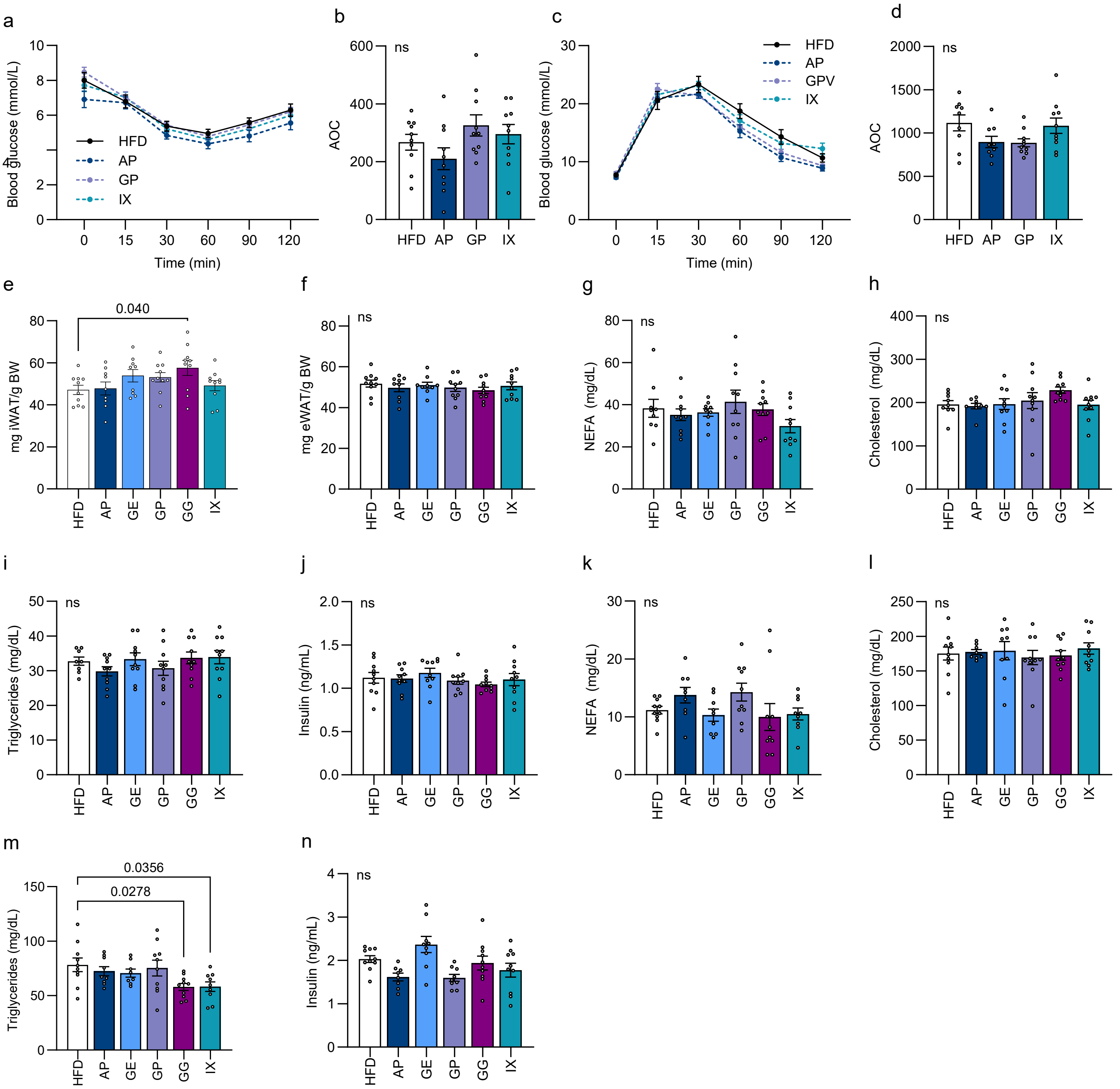

Supplementary Fig. 9 (related to Fig. 5): Effects of F4 metabolites on metabolic health. (a-b) (a) Insulin tolerance test (HFD 6 weeks, DI=6 weeks) and (b) AOC. HFD n=10, AP n=10, GP n=10, IX n=10. (c-d) (d) Glucose tolerance test (i.p, DI=7 weeks) and (d) AOC. HFD n=9, AP n=9, GP n=10, IX n=10. (e-f) (e) iWAT and (f) eWAT mass normalized to body weight (HFD=6 weeks, DI=9 weeks). HFD n=10, AP n=9, GE n=9, GP n=10, GG n=10, IX n=10. (g-h) Fasting blood profiles for (g) NEFA (HFD n=9, AP n=10, GE n=9, GP n=10, GG n=10, IX n=10), (h) cholesterol (HFD n=9, AP n=9, GE n=10, GP n=10, GG n=10, IX n=10), (i) triglycerides (HFD n=10, AP n=10, GE n=9, GP n=10, GG n=10, IX n=10) and (j) insulin concentrations (HFD n=10, AP n=10, GE n=9, GP n=10, GG n=10, IX n=10) after HFD=6 weeks, DI=4 weeks. (k-n) Random fed plasma profiles for (k) NEFA (HFD n=10, AP n=9, GE n=9, GP n=10, GG n=10, IX n=9), (l) cholesterol (HFD n=10, AP n=8, GE n=9, GP n=10, GG n=10, IX n=10), (m) triglycerides (HFD n=10, AP n=9, GE n=8, GP n=10, GG n=10, IX n=9) and (n) insulin levels (HFD n=10, AP n=8, GE n=9, GP n=9, GG n=10, IX n=10) after HFD=6 weeks, DI=9 weeks. Data are presented as mean ± SEM. All comparisons against HFD. One-way ANOVA with Dunnett´s post-hoc test was applied in b, d, e, f, g, h, i, j, k, l, m, n. Statistical test results are indicated as exact p-values with *p<0.05 considered significant. Source data are provided in Source Data 1. DI, Dietary intervention; NEFA, non-esterified fatty acids; iWAT, inguinal white adipose tissue; eWAT, epididymal white adipose tissue; HFD, high fat diet; IX, isovitexin; AP, apigenin; GP, Genkwanin 5-*O*-primeveroside; GE, Genkwanin; GG, Genkwanin 5-*O*-glucoside.

Supplementary Fig. 10

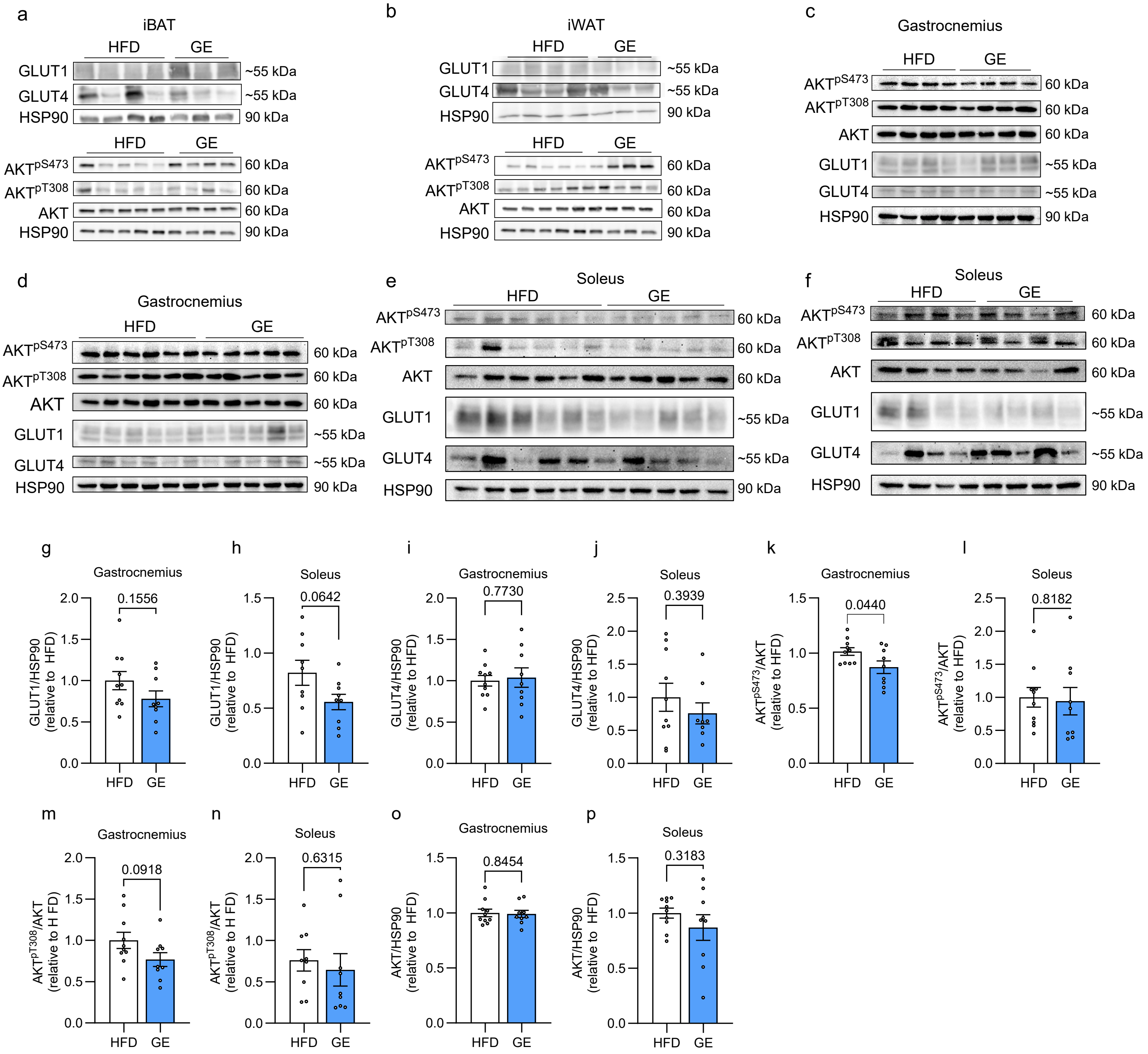

Supplementary Fig. 10 (related to Fig 6): Insulin signaling pathways and glucose transporter levels in adipose depots and skeletal muscles from GE-treated mice. (a-b) Additional western blots contributing to datapoints displayed in Fig. 5 j-k and r-s. (c-f) AKT phosphorylation and GLUT levels in (c-d) gastrocnemius muscle and (e-f) soleus muscles in HFD-control and GE-treated mice.(g-n) respective quantifications of GLUT1 in (g) gastrocnemius (HFD n=10, GE n=9) and (h) soleus (HFD n=10, GE n=9), GLUT4 in (i) gastrocnemius (HFD n=10, GE n=9) and (j) soleus (HFD n=10, GE n=8), AKT^{pS473} in (k) gastrocnemius (HFD n=10, GE n=9) and (l) soleus (HFD n=9, GE n=9), AKT^{pT308} in (m) gastrocnemius (HFD n=10, GE n=9) and (n) soleus (HFD n=9, GE n=9) and AKT in (o) gastrocnemius (HFD n=10, GE n=9) and (p) soleus (HFD n=10, GE n=9). Data are presented as mean ± SEM. Student´s t-test was applied in g, n, **l**, j, k, l, m, n, o, p. Statistical test results are indicated as exact p-values with *p<0.05 considered significant. Source data are provided in Source Data 1. GE, Genkwanin; HFD, high fat diet.

Supplementary Fig. 11

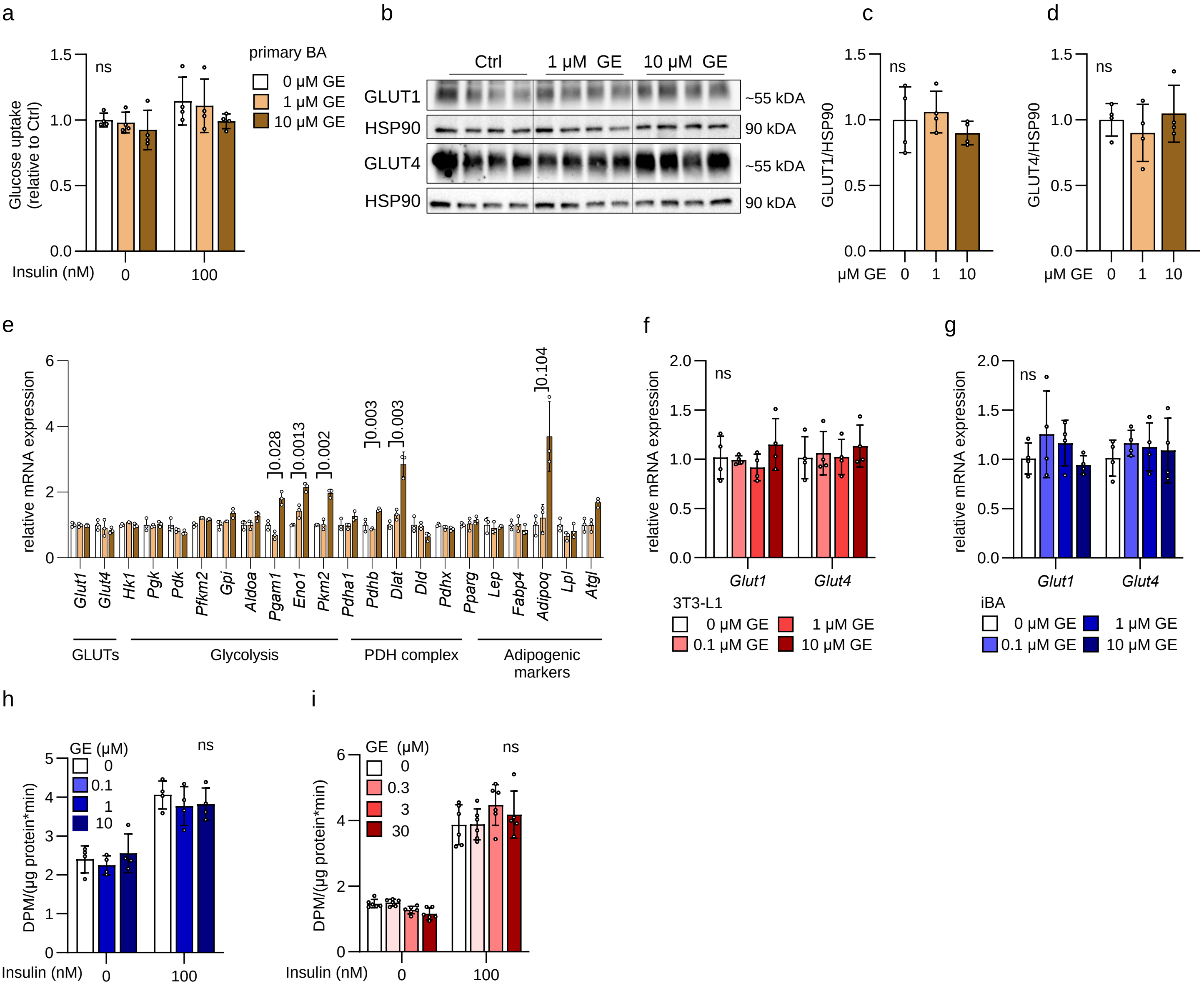

Supplementary Fig. 11 (related to Fig. 6): Effects of Genkwanin on glucose metabolism in adipocyte models. (a-b) Glucose uptake in primary murine BAs after 3 days of GE treatment (n=4/condition). (c-d) GLUT1 and GLUT4 protein levels in primary BAs after 3 days of GE treatment (n=4/condition). (e) Gene expression analysis of targets regulating glucose metabolism and adipogenic markers in primary BAs after 3 days of GE treatment (n=3/condition). (f-g) Glucose transporter mRNA expression in (f) iBAs (n=4/condition) and (g) 3T3-L1 adipocytes (n=4 /condition) after 3 days of GE treatment. (h) Glucose uptake in iBAs after 2 days of GE treatment (n=4/condition). (i) Glucose uptake in 3T3-L1 adipocytes after 16 hours of GE treatment (n=6/condition, except GE 30 uM, 100 nM insulin n=5). 1 independent experiment for all panels. Data are presented as mean ± SD. two-way ANOVA with Sidak´s post-hoc test was applied in a, h, i. Comparisons were grouped into one family to control FWER. One-way ANOVA with Dunnett´s post-hoc test was applied in c and d. One-way ANOVA with Dunnett´s post-hoc test was applied per gene in e-g, with Sidak correction across all genes. All comparisons were performed against untreated controls (0 μM). Statistical test results are indicated as exact p-values with *p<0.05 considered significant. Source data are provided in Source Data 1. GE, Genkwanin; iBA, immortalized brown adipocytes; BA, brown adipocytes, GLUTs, glucose transporters; DPM, decays per minute.

Supplementary Fig. 12

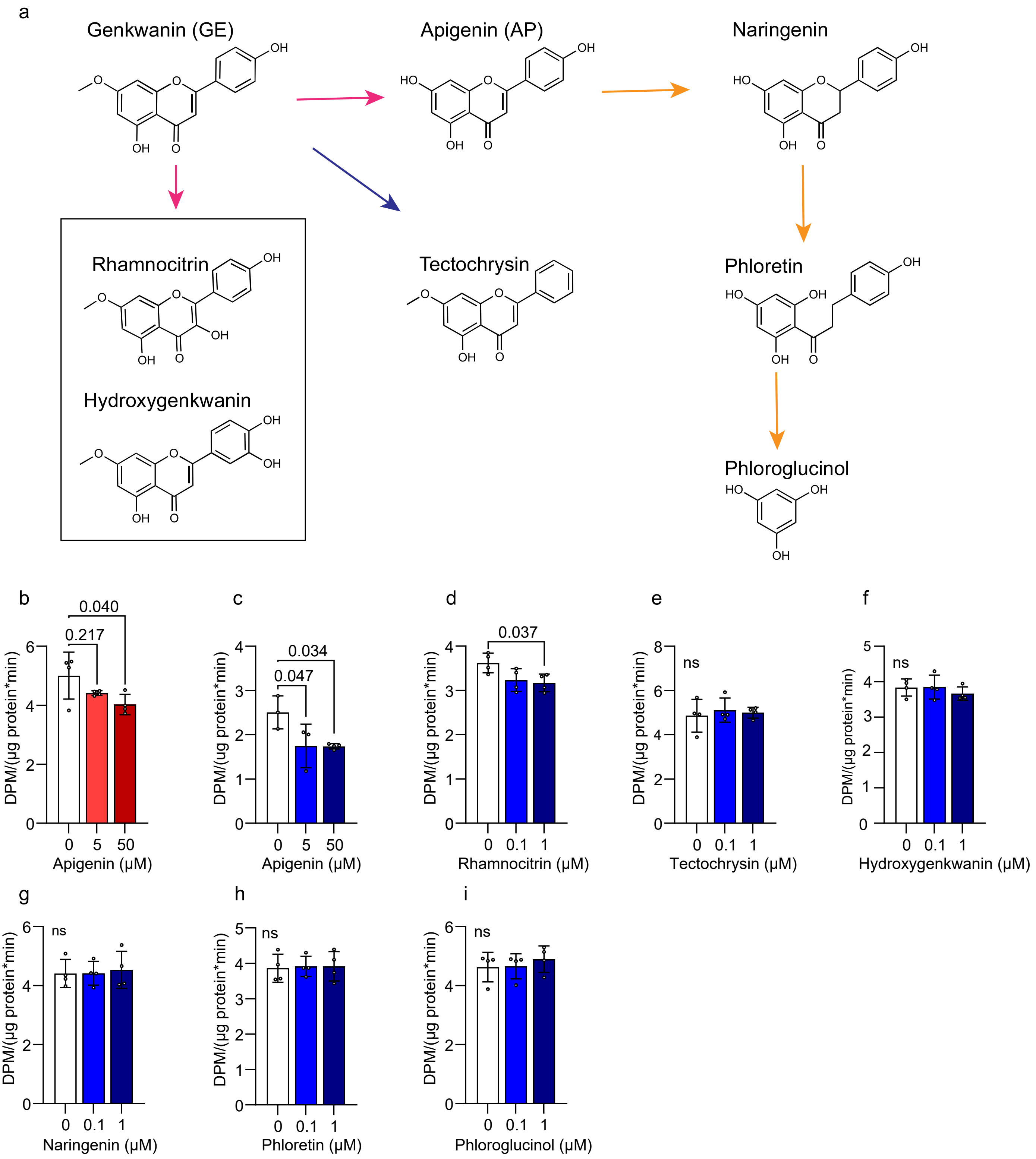

Supplementary Figure 12 (related to Fig. 6): Putative genkwanin biotransformation products and their in vitro efficacy. (a) Chemical structures of potential metabolites resulting from the biotransformation of the aglycone genkwanin (GE). Pink arrow represents a phase 1 biotransformation predicted by BioTransformer 3.0. Blue arrow represents a gut metabolism biotransformation predicted by BioTransformer 3.0. Orange arrow represents a gut metabolism biotransformation as described in[1] (Schoefer, Mohan et al. 2003). (b-i) Glucose uptake rates in (b) 3T3-L1 (n=4/condition) and (c-i) iBAs after treatment with the indicated biotransformation products for 3 days (Apigenin 0 µM n=3, 5 µM n=3, 50 µM n=4; Rhamnocitrin n=4/condition; Tectochrysin n=4 per condition; Hydroxygenkwanin: n=4/condition; Naringenin n=4/condition; Phloretin n=4/condition; Phloroglucinol n=4/condition). 1 independent experiment for all panels. One-way ANOVA with Dunnett´s post-hoc test was applied b, c, d, e, f, g, h, i. All comparisons against untreated controls (0 µM). Statistical test results are indicated as exact p-values with *p<0.05 considered significant. Source data are provided in Source Data 1. iBAs, immortalized brown adipocytes.

Supplementary Table 1.

Summary of metabolic effects of PNe, PNe fractions and isolated compounds in diet-induced obese mice, categorized by metabolic phenotyping, biochemical parameters and AKT-signaling in tissues.

Metabolic Phenotyping

**In vivo read-out**	**PNe**	**F4**	**F3**	**F2**	**MET**	**GE**	**GG**	**IX**	**AP**	**GP**
Insulin tolerance	Up: 0.007	Up: 0.045	Unchanged	Unchanged	Unchanged	Unchanged	Unchanged	Unchanged	Unchanged	Unchanged
Glucose tolerance	Unchanged	Down: 0.004	Down: 0.021	Down: 0.005	Unchanged	Down: 0.005	Down: 0.0002	Unchanged	Unchanged	Unchanged
Glucose uptake muscle	Unchanged	-	-	-	Unchanged	Unchanged	-	-	-	-
Glucose uptake iBAT	Unchanged	-	-	-	Unchanged	Up: 0.011	-	-	-	-
Glucose uptake iWAT	Up: 0.004	-	-	-	Up: 0.023	Up: 0.017	-	-	-	-
Glucose uptake eWAT	Unchanged	-	-	-	Up: 0.001	Up: 0.04	-	-	-	-
GLUT1 protein iBA/iWAT	Up: 0.035,0.032	-	-	-	-	Unchanged	-	-	-	-
GLUT4 protein iBAT/iWAT	Unchanged	-	-	-	-	Unchanged	-	-	-	-
Glucose infusion rate (GIR)	-	Up: 0.038	-	-	Up: 0.002	Up: 0.029	-	-	-	-
GUR eWAT	-	Unchanged	-	-	Unchanged	-	-	-	-	-
GUR iWAT	-	Up: 0.043	Unchanged	Unchanged	Up: 0.011	-	-	-	-	-
GUR iBAT	-	Up: 0.049	-	-	Up: 0.005	-	-	-	-	-
GUR soleus	-	Up: 0.017	-	-	Up: <0.001	-	-	-	-	-
GUR gastrocnemius	-	Up: 0.023	-	-	Unchanged	-	-	-	-	-
iWAT mass	-	-	-	-	-	Unchanged	Unchanged	Unchanged	Unchanged	Unchanged
eWAT mass	-	-	-	-	-	Unchanged	Unchanged	Unchanged	Unchanged	Unchanged
eWAT/iWAT ratio	-	-	-	-	-	Unchanged	Down: 0.012	Unchanged	Unchanged	Unchanged

Abbreviations: AP, apigenin; GE, Genkwanin; GG, genkwanin-5-*O*-β-D-glucoside; GP, genkwanin-5-*O*-β-primeveroside; IX, isovitexin; PNe, Phaleria nisidai extract; MET, metformin; GUR, glucose uptake rates (clamps); GSIS, glucose-stimulated insulin secretion; eWAT, epididymal white adipose tissue; iWAT, inguinal white adipose tissue; iBAT, interscapular brown adipose tissue; ALT, alanine transaminase; NEFA, non-esterified fatty acids.

Continued Supplementary Table 1.

Biochemical Parameters

In vivo read-out	PNe	F4	F3	F2	GE	GG	IX	AP	GP
Fasting blood glucose	Unchanged	Down: 0.0128	Down: 0.0377	Down: 0.0230	-	-	-	-	-
Random fed blood glucose	-	-	-	-	Down: 0.047	Unchanged	Unchanged	Unchanged	Unchanged
GLP-1 plasma levels	Unchanged	Unchanged	Up: 0.012	Unchanged	-	-	-	-	-
Fasted plasma NEFA	Unchanged	-	-	-	Unchanged	Unchanged	Unchanged	Unchanged	Unchanged
Fasted plasma cholesterol	Unchanged	-	-	-	Unchanged	Unchanged	Unchanged	Unchanged	Unchanged
Fasted plasma TAG	Unchanged	-	-	-	Unchanged	Unchanged	Unchanged	Unchanged	Unchanged
Fasted plasma insulin	Down: 0.0199	-	-	-	-	-	-	-	-
Fasted plasma ALT	Unchanged	Unchanged	Unchanged	Unchanged	-	-	-	-	-
GSIS	Down: 0.0036	-	-	-	-	-	-	-	-
Urinary glucose	Down: 0.0306	-	-	-	Up: 0.002	Unchanged	Unchanged	Unchanged	Unchanged
Random fed plasma NEFA	-	-	-	-	Unchanged	Unchanged	Unchanged	Unchanged	Unchanged
Random fed plasma cholesterol	-	-	-	-	Unchanged	Unchanged	Unchanged	Unchanged	Unchanged
Random fed plasma TAG	-	-	-	-	Unchanged	Down: 0.0278	Down: 0.0356	Unchanged	Unchanged
Random fed plasma insulin	-	-	-	-	Unchanged	Unchanged	Unchanged	Unchanged	Unchanged

AKT-Signaling

In vivo read-out	PNe	GE	In vivo read-out	PNe	GE	In vivo read-out	PNe	GE
pAKT(s473)/AKT iWAT	-	Up: 0.0068	pAKT(t308)/AKT soleus	-	Unchanged	GLUT4 iWAT	Unchanged	Unchanged
pAKT(s473)/AKT iBAT	-	Up: 0.0488	AKT gastrocnemius	-	Unchanged	GLUT4 iBAT	Unchanged	Unchanged
pAKT(s473)/AKT gastrocnemius	-	Down: 0.044	AKT soleus	-	Unchanged	GLUT4 gastrocnemius	-	Unchanged
pAKT(s473)/AKT soleus	-	Unchanged	GLUT1 iWAT	Up: 0.032	Unchanged	GLUT4 soleus	-	Unchanged
pAKT(t308)/AKT iWAT	-	Unchanged	GLUT1 iBAT	Up: 0.035	Unchanged	AKT iWAT	-	Unchanged
pAKT(t308)/AKT iBAT	-	Unchanged	GLUT1 gastrocnemius	-	Unchanged	AKT iBAT	-	Unchanged
pAKT(t308)/AKT gastrocnemius	-	Unchanged	GLUT1 soleus	-	Unchanged			

Supplementary Table 2.

Murine and human qPCR primer sequences obtained from Microsynth.

	Target gene	frd primer	rev primer
Mouse	*Gpi*	CTTCGACCAGTGGGGAGTG	CTAGTTTGGTGTCCCGCTGT
	Aldoa	CGCTCCTTAGTCCTTTCGCC	GACAGGCGGGTCATGTTGAA
	Pgam1	GGTGAGGCCCAGGTAAAGAT	CTGCGTACCTGCGATCCTTG
	Eno1	CTTCATGGGGAAGGGCGTCT	GCCGTCCATCTCGATCATCA
	Pkm2	GCGACTCGTCTTCACTTGAC	GTTCCTGAAGTCCTCGGGC
	Pdha1	AGATGCTTGCCGCTGTATCC	GCCGATGAAGGTCACATTTCTTAAT
	Pdhb	ACGGTGCATACAAGGTTAGCA	TCTGAGATGGGGGTGTCGAT
	Dlat	CCTGTAGCACCTACCCCCT	CTGGTCCCGTCCCTTTAACT
	Dld	GAGCTGGAGTCGTGTGTACC	TCACGTCAGCCTCAATTGGT
	Pdhx	CTATCCTCGGCCAATGACCC	TTCGGTGAATGTACCCGCTG
	Glut1	GGATCCCAGCAGCAAGAAGGT	CTCCGTAGCGGTGGTTCCAT
	Glut4	GACGGACACTCCATCTGTTG	GCCACGATGGAGACATAGC
	Hk1	ACCAAAGTGTACCGTGTCCT	TTAGGCGTTCGTAGGGTCTC
	Pgk	CCATAGCTCCATGGTGGGTG	TTAGCGCCTCCCAAGATAGC
	Pdk	GCCAGGTGGACTTCTATGCG	ACGGATGGGGTCCTGAGAAG
	Pfkm2	AGATCGTAGACGCCATCACC	GGCCCATCACTTCTAACACAA
	Pparg	AGGCGAGGGCGATCTTGACA	CGGATGGCCACCTCTTTGGTCT
	Fabp4	AAGGTGAAGAGCATCATAACCCT	TCACGCCTTTCATAACACATTCC
	Adipoq	GGAGAGAAAGGAGATGCAGGT	CTTTCCTGCCAGGGGTTC
	Atgl	CTGAGAATCACCATTCCCACATC	CACAGCATGTAAGGGGGAGA
	Lpl	GAAAGGGCTCTGCCTGAGTT	TAGGGCATCTGAGAGCGAGT
	Lep	CAGGATCAATGACATTTCACACA	GCTGGTGAGGACCTGTTGAT
	36B4	GCCGTGATGCCCAGGGAAGA	CATCTGCTTGGAGCCCACGTT
	Tbp	GAAGCTGCGGTACAATTCCAG	CCCCTTGTACCCTTCACCAAT

	Target gene	frd primer	rev primer
Human	*GLUT1*	GGATCCCAGCAGCAAGAAGGT	CTCCGTAGCGGTGGTTCCAT
	GLUT4	CTTCATCATTGGCATGGGTTT	AGGACCGCAAATAGAAGGAAGA
	TBP	CACGAACCACGGCACTGATT	TTTTCTTGCTGCCAGTCTGGAC

Supplementary References

1. Schoefer, L., Mohan, R., Schwiertz, A., Braune, A. & Blaut, M. Anaerobic degradation of flavonoids by Clostridium orbiscindens. *Appl Environ Microbiol* **69**, 5849–5854 (2003).
