# Supplementary material for: Genkwanin glycosides are major active compounds in Phaleria nisidai extract mediating improved glucose homeostasis by stimulating glucose uptake into adipose tissues
Source: Nat Commun. 2025 Aug 16;16:7648. doi: 10.1038/s41467-025-62689-8 (PMC12357923; doi:10.1038/s41467-025-62689-8)
Supplement: Supplementary file 2 — Reporting Summary [file 41467_2025_62689_MOESM2_ESM.pdf]

nature portfolio

Corresponding author(s): Christian Wolfrum
Jean-Luc Wolfender

Last updated by author(s): 2025.07.09

Reporting Summary

Nature Portfolio wishes to improve the reproducibility of the work that we publish. This form provides structure for consistency and transparency in reporting. For further information on Nature Portfolio policies, see our Editorial Policies and the Editorial Policy Checklist.

Statistics

For all statistical analyses, confirm that the following items are present in the figure legend, table legend, main text, or Methods section.

n/a	Confirmed	
☐	☒	The exact sample size (*n*) for each experimental group/condition, given as a discrete number and unit of measurement
☐	☒	A statement on whether measurements were taken from distinct samples or whether the same sample was measured repeatedly
☐	☒	The statistical test(s) used AND whether they are one- or two-sided<br>*Only common tests should be described solely by name; describe more complex techniques in the Methods section.*
☒	☐	A description of all covariates tested
☐	☒	A description of any assumptions or corrections, such as tests of normality and adjustment for multiple comparisons
☐	☒	A full description of the statistical parameters including central tendency (e.g. means) or other basic estimates (e.g. regression coefficient) AND variation (e.g. standard deviation) or associated estimates of uncertainty (e.g. confidence intervals)
☐	☒	For null hypothesis testing, the test statistic (e.g. *F*, *t*, *r*) with confidence intervals, effect sizes, degrees of freedom and *P* value noted<br>*Give P values as exact values whenever suitable.*
☒	☐	For Bayesian analysis, information on the choice of priors and Markov chain Monte Carlo settings
☒	☐	For hierarchical and complex designs, identification of the appropriate level for tests and full reporting of outcomes
☒	☐	Estimates of effect sizes (e.g. Cohen's *d*, Pearson's *r*), indicating how they were calculated

Our web collection on statistics for biologists contains articles on many of the points above.

Software and code

Policy information about availability of computer code

Data collection

Western blots were acquired with the Image Quant (GE Healthscience). Real time qPCRs were performed with the applied biosystem Viia 7 machine. Fluorescence microscope images were aquired with the Operetta microscope (Perkin Elmer). Protein concentrations and colorimetric assays were measured with the SynergyMx plate reader (BioTek). Cellular respiration was monitored by XF96 Extracellular Flux Analyzer (Agilent Seahorse). Cumulative food intake measurements were performed using the automated Phenomaster metabolic cage system (TSE-systems). Body composition of alive mice was measured with a magnetic resonance imaging technique (EchoMRI 30, Echo Medical Systems). Harvard appartus was used as infusion pump during the clamp experiments. Chromeleon 7.2.9 (UHPLC-PDA-CAD) and Xcalibur 2.1 (UHPLC-PDA-HRMS/MS) softwares were used for the chemical profile aquisition: MassLynx V4.2 (UHPLC-PDA-QMS-ELSD) Software was used to acquire the fractions. Radioactive counts were measured with a TriCarb Beta-Counter (Perkin Elmer).

Data analysis

Densimetry of Western blots was done by ImageJ/Fiji (Image J1.54F). For qPCR analysis, the data was exported to excel from the ViiA7 and analysed using the delta Ct method. Immunofluorescence image analysis was performed by Harmony v3.5 (Perkin Elmer) and Phyton (3.12.7) for quantification of nuclear localized alexa488 staining. Oxygen consumption rates and ECAR data were collected with the Wave 2.6.0 (Agilent Seahorse) software and analysed using Microsoft Excel. Cumulatitve food intake was assessed by Phenomaster software v5.6.5 (TSE-systems). Fatatistical analysis was performed by GraphPad Prism versions 6-10.
Software used for the chemical profile analysis: Chromeleon 7.2.9 (UHPLC-PDA-CAD), Xcalibur 2.1 (UHPLC-PDA-HRMS/MS).
Software used to analyze the fractions: MassLynx V4.2 (UHPLC-PDA-QMS-ELSD), MNOVA 14 (NMR). Software used to present the UHPLC-PDA-CAD metabolite profiling: GraphPad Prism version 6. Software used to drawn the chemical structures: ChemDraw 22.2.0. Software used for HRMS spectra presentation: MZmine 2.53.

For manuscripts utilizing custom algorithms or software that are central to the research but not yet described in published literature, software must be made available to editors and reviewers. We strongly encourage code deposition in a community repository (e.g. GitHub). See the Nature Portfolio guidelines for submitting code & software for further information.

Data

Policy information about availability of data

All manuscripts must include a data availability statement. This statement should provide the following information, where applicable:

- Accession codes, unique identifiers, or web links for publicly available datasets
- A description of any restrictions on data availability
- For clinical datasets or third party data, please ensure that the statement adheres to our policy

Bio guided fractionation data generated in this study have been deposited in the YARETA repository under DOI: https://doi.org/10.26037/yareta:2j2b4d5ivjeudbcsbxmvxcpgiq. This data includes the chemical profiling of PNe and its four fractions obtained by UHPLC-PDA-HRMS/MS (raw data), the chemical profiling of fractions F2 and F4 obtained by UHPLC-PDA-QDA-MS-ELSD, and NMR spectra of the four fractions and all isolated constituents. UHPLC-HRMS/MS mzML data have been deposited in MassIve under https://doi.org/doi:10.25345/C51G0J70D. The fragmentation spectra of all isolated compounds have been deposited in the GNPS library. The direct links to the spectra are provided in the method section "Description of the isolated compounds". NMR, HRMS and UV data generated in this study are also provided in the Source Data file 2.
The code for image analysis is deposited on Github (DOI: 10.5281/zenodo.15767222).
Raw images are deposited on Zenodo (https://zenodo.org/records/15231500)
The source data for each panel is available in the paper in Source Data 1.

Research involving human participants, their data, or biological material

Policy information about studies with human participants or human data. See also policy information about sex, gender (identity/presentation), and sexual orientation and race, ethnicity and racism.

Reporting on sex and gender	Strom vascular fraction was isolated from abdominal subcutaneous adipose tissue biopsy from one male subject.
Reporting on race, ethnicity, or other socially relevant groupings	Sex of human participants is not relevant for this study.<br>The manuscript does not include any data or analyses where socially relevant groupings or ethnicity could be of relevance.
Population characteristics	No human samples from a larger population were characterized or analyzed.
Recruitment	No human subjects were specifically recruited for the study. Biospy was taken as part of routine surgery procedure.
Ethics oversight	The study protocol was approved by the Ethical committee of the University Hospital in Bratislava and it conforms to the ethical guidelines of the 2000 Helsinki declaration.

Note that full information on the approval of the study protocol must also be provided in the manuscript.

Field-specific reporting

Please select the one below that is the best fit for your research. If you are not sure, read the appropriate sections before making your selection.

☒ Life sciences ☐ Behavioural & social sciences ☐ Ecological, evolutionary & environmental sciences

For a reference copy of the document with all sections, see nature.com/documents/nr-reporting-summary-flat.pdf

Life sciences study design

All studies must disclose on these points even when the disclosure is negative.

Sample size	The sample size was determined based on previous experience and publications in our group (Balaz et al., 2019 Cell Metab, Sun et al., 2020 Nature, Sun et al., 2018 Nature Medicine). For the glucose clamp studies, we assumed an effect size of 25% (incrase/decrease in GIR) and an estimated power of 80% with alpha=0.05, which requires an n=6 per condition. Including an estimated maximum drop-out rate of 30%, n=10 animals were included per group to obtain relevant results.
Data exclusions	Exclusion of animals during clamp study: Animals loosing >10% BW compared to pre-operative weight were excluded (n=2). During the clamp procedures, some animals were excluded to due technical problems (dissconected from tubing during SS, no stable SS, infusion pump not properly, cathether/connecting tubing leaking) or due to macroscopically unhealthy internal appearance, n=1). This resulted in unequal n-numbers for different readouts from the clamps. For all other experiments, datasets with suspected outliers were tested with Grubb's test (Graphpad prism) or removed due to known technical issues affecting sample availability, quality or data aquisition (urine collection, Hemolysis in blood plasma, failed injection seahorse).
Replication	Cell culture experiments were repeated 2-3 times depending on the experiment with at least 3 biological replicates per condition. Glucose uptake assay in C2C12 muscle cells , ERK1/2 signaling in hMADS and lipolysis assays were performed once. Glucose uptake assays with compounds from biotransformation predictions were performed once. The exact number of independen repetitions is stated in the figure legends. In vivo results from PNe-fed animals were collected from two independent cohorts as outlined in the results sections. Results form the PNe fractionation were generated from one cohort. Comparison of F4 against metformin was performed in two independent cohorts. The second cohort was used for clamp studies. The effects of genkwanin and glucogenkwanin on insulin sensitivity was assessed in two

independent cohorts. The ITT results are displayed as pooled data. Glucose clamps with GE-fed animals were performed in an independent cohort. Replication attempts were generally successful, with the following exceptions: In one trial involving PNe stimulation with a PKC inhibitor, the expected inhibitory effect was absent. We suspect this was due to the use of degraded reagents resulting from repeated freeze-thaw cycles. Additionally, one glucose uptake experiment in primary brown adipocytes failed to show an effect, likely due to poor cell differentiation.

Randomization

Animals were fed for HFD for the indicated periods of time with n=4-5 animals per cage. When allocating the mice to the different dietary interventions we recorded the body weight from each animal and allocated randomly into weight matched group. To achieve this, cages were split up to contain 2 or 3 animals. Animals were not mixed between cages. Animals which did not respond to the HFD were excluded. Animals were further randomly allocated to a box in the metabolic cage system or assigned to a pump during the clamps (3 animals per day). For in vitro experiments, culture wells were randomly assigned to treatments in each experiment to avoid any plate effect.

Blinding

The investigators were not blinded in the dietary intervention study as they were responsible for both preparing of the special diet and feeding the animals. Blinding was not feasible because accurately assigning the appropriate diet to each group required knowledge of the specific dietary allocations. For metabolic tests, animals from different groups were tested in random order and in alternating order between different treatment groups.

Reporting for specific materials, systems and methods

We require information from authors about some types of materials, experimental systems and methods used in many studies. Here, indicate whether each material, system or method listed is relevant to your study. If you are not sure if a list item applies to your research, read the appropriate section before selecting a response.

Materials & experimental systems

n/a	Involved in the study	
☐	☒	Antibodies
☐	☒	Eukaryotic cell lines
☒	☐	Palaeontology and archaeology
☐	☒	Animals and other organisms
☒	☐	Clinical data
☒	☐	Dual use research of concern
☐	☒	Plants

Methods

n/a	Involved in the study	
☒	☐	ChIP-seq
☒	☐	Flow cytometry
☒	☐	MRI-based neuroimaging

Antibodies

Antibodies used

AKT Cell Signaling #9272; pAKT S473 Cell Signaling #4060, pAKT T308 Cell Signaling #13038, HSP90 Cell Signaling #4877, phospho-p42/44 MAPK Thr202/Tyr204 Cell Signaling #4370S, p42/p44 MAPK Cell Signaling #4695, Glut1 Merck #07-1401, GLUT4 Merck #07-1404, phospho-PKC Substrate Cell Signaling #2261S, yTublin Sigma-Aldrich #T-5326, GADPH Cell Signaling #2118S, Anti-rabbit HRP-conjugated secondary Merck Milipore #401393, Anti-mouse HRP-conjugated primary Merck Millipore #401253, IDonkey anti-rabbit Alexa Fluor 488 Invitrogen #A21206

Validation

Many of the applied primary antibodies (AKT, AKT S473, pAKT T308, HSP90, yTUBULIN, p42/44 MAPK, phospho-p42/44 MAPK Thr202/Tyr204, Glut1, GLUT4, GADPH) and the conjugated-secondary antibodies are validated for their application (WB or IF) by the manufacturer and are routinely used as well as cited in the literature.
Manufacturer validation
AKT: https://www.cellsignal.com/products/primary-antibodies/akt-antibody/9272?srsltid=AfmBOorSl_F2hO71rggAb-8ImR8OmdZx8RijgXgBVR8_CithpmpbZQJM
pAKT T308: https://www.cellsignal.com/products/primary-antibodies/phospho-akt-thr308-d25e6-xp-rabbit-mab/13038?srsltid=AfmBOoqR6lgG6__iUXtklzY4Djz8ceggroBb_Yexc3-gFMxdawLFJz_c
pAKT S473: https://www.cellsignal.com/products/primary-antibodies/phospho-akt-ser473-d9e-xp-rabbit-mab/4060
HSP90: https://www.cellsignal.com/products/antibody-conjugates/hsp90-c45g5-rabbit-mab-hrp-conjugate/79641
p42/p44 MAPK: https://www.cellsignal.com/products/primary-antibodies/p44-42-mapk-erk1-2-137f5-rabbit-mab/4695
Glut1: https://www.merckmillipore.com/CH/de/product/Anti-GLUT-1-Antibody-CT,MM_NF-07-1401?ReferrerURL=https%3A%2F%2Fwww.google.com%2F
Glut4: https://www.sigmaaldrich.com/CH/fr/product/mm/071404?mmredirect=1&srsltid=AfmBOopGB28GYguBIEZ2sIwjMG7BViuYhXfhTK5noIk9jSUaXleiM6RA
phosphoPKC: https://www.cellsignal.com/products/primary-antibodies/phospho-ser-pkc-substrate-antibody/2261

The phospho-PKC Substrate antibody was validated using the PKC agonist PMA for WB application by Cell Signaling and in our Figure 3.
yTubulin: https://www.sigmaaldrich.com/CH/fr/product/sigma/t5326
GADPH:https://www.cellsignal.com/products/primary-antibodies/gapdh-14c10-rabbit-mab/2118

Eukaryotic cell lines

Policy information about cell lines and Sex and Gender in Research

Cell line source(s)	Murine immortalized brown adipocytes (iBAs) - prof. Ronald C. Kahn, Harvard University, Boston, USA (Klein et al., 2002)<br>hMADS cells - Dr. Ez-Zoubir Amri, University of Nice, France (Elabd et al., 2009)<br>3T3-L1 fibroblasts (CL-173) and C2C12 (CRL-1772) were obtained from ATCC.<br>Primary subcutaneous human stromal vascular fraction was obtained in collbaroation with Dr. Miroslav Balaz and Dr. Jozef Ukropec, Biomedical. Research Center of the Slovak Academy of Sciences, Slovaka (Balaz et al., 2024, Malisova et al., 2014).<br>Stormal vascular fraction isolated from murine inguinal white adipose tissue or interscapular brown adipose tissue was obtained from in house bred or commerically available (Charles River Laboratories) C57B6/N mice.
Authentication	Immortalized brown adipocytes and hMADS are a commonly used model to study the thermogenic function of brown adipocytes after differentiation. Their authenticy was confirmed by UCP1 expression via qPCR and increased oxygen consumption in the seahorse extracellular flux analysis after isoporterenol or cAMP administration. 3T3-L1 cells origin from Swiss Albino mice and are a long established model to investigated white adipocytes, they express common adipogenic markers such as GLUT4, PARRg or FABP4.
Mycoplasma contamination	The lines were routinely tested for mycoplasma contamination and tested negative.
Commonly misidentified lines<br>(See ICLAC register)	No commonly misidentified cell lines were used.

Animals and other research organisms

Policy information about studies involving animals; ARRIVE guidelines recommended for reporting animal research, and Sex and Gender in Research

Laboratory animals	Mus musculus, strain C57BL6/N (Charles River or in house breeding)
Wild animals	No wild animals were used as part of the study.
Reporting on sex	Male mice were used in study because female mice do not respond well to high-fat diet feeding.
Field-collected samples	The leaves of the tree Phaleria nishidai Kaneh. (Thymelaeaceae) were collected in 2017 on the island of the Republic of Palau. Reference specimens were deposited at the Belau National Museum Herbarium.
Ethics oversight	All animal experiments were approved by the Cantonal Veterinary Office of the Canton of Zurich, Switzerland (ZH220/2020, ZH221/2020). Health status of all mouse lines was regularly monitored according to FELASA guidelines.

Note that full information on the approval of the study protocol must also be provided in the manuscript.

Dual use research of concern

Policy information about dual use research of concern

Hazards

Could the accidental, deliberate or reckless misuse of agents or technologies generated in the work, or the application of information presented in the manuscript, pose a threat to:

No	Yes	
☒	☐	Public health
☒	☐	National security
☒	☐	Crops and/or livestock
☒	☐	Ecosystems
☒	☐	Any other significant area

Experiments of concern

Does the work involve any of these experiments of concern:

No	Yes	
☒	☐	Demonstrate how to render a vaccine ineffective
☒	☐	Confer resistance to therapeutically useful antibiotics or antiviral agents
☒	☐	Enhance the virulence of a pathogen or render a nonpathogen virulent
☒	☐	Increase transmissibility of a pathogen
☒	☐	Alter the host range of a pathogen
☒	☐	Enable evasion of diagnostic/detection modalities
☒	☐	Enable the weaponization of a biological agent or toxin
☒	☐	Any other potentially harmful combination of experiments and agents

Plants

Seed stocks	The leaves of the tree Phaleria nishidai Kaneh. (Thymelaeaceae) were collected in 2017 on the island of the Republic of Palau. Reference specimens were deposited at the Belau National Museum Herbarium.
Novel plant genotypes	Does not apply.
Authentication	Does not apply.
