# Supplementary material for: Genkwanin glycosides are major active compounds in Phaleria nisidai extract mediating improved glucose homeostasis by stimulating glucose uptake into adipose tissues
Source: Nat Commun. 2025 Aug 16;16:7648. doi: 10.1038/s41467-025-62689-8 (PMC12357923; doi:10.1038/s41467-025-62689-8)
Supplement: Supplementary file 4 — Source Data [file 41467_2025_62689_MOESM4_ESM.zip › SourceData_2.pdf]

Source Data 2

Genkwanin glycosides are major active compounds in *Phaleria nisidai* extract mediating improved glucose homeostasis by stimulating glucose uptake into adipose tissues

Carla Horvath[1$], Joëlle Houriet[2,3$], Alexandra Kellenberger[1$], Caroline Moser[1], Lucia Balazova[1,4], Miroslav Balaz[1,4], Hua Dong[6], Aron Horvath[7], Isabel Reinisch[1], Vissarion Efthymiou[1], Adriano Rutz[2,3], Laurence Marcourt[2,3], Christopher Kitalong[7], Bertrand Graz[3,8,9], Victor Yano[8], Emerson Ferreira Queiroz[2,3], Jean-Luc Wolfender[2,3] and Christian Wolfrum[1,10*]*

[1]*Institute of Food, Nutrition and Health, ETH Zurich, Schorenstr. 16, 8603 Schwerzenbach, Switzerland*

[2]*School of Pharmaceutical Sciences, University of Geneva, CMU, Rue Michel Servet 1, 1211 Geneva, Switzerland*

[3]*Institute of Pharmaceutical Sciences of Western Switzerland, University of Geneva, CMU, Rue Michel Servet 1, 1211 Geneva, Switzerland*

[4]*Biomedical Research Center, Slovak Academy of Sciences, Dubravska cesta 9, 845 05 Bratislava, Slovakia.*

[5]*Department of Animal Physiology and Ethology, Faculty of Natural Sciences, Comenius University, Ilkovicova 6, 841 04 Bratislava, Slovakia*

[6]*Institute for Stem Cell Biology and Regenerative Medicine, Stanford University School of Medicine, Stanford, CA 94305, USA*

[7]*Institute of Biomechanics, ETH Zurich, Balgrist Campus, Switzerland*

[8]*Pacific Academic Institute for Research, 822 Ernguul Rd., Koror, Palau*

[9]*Community Health Association – Geneva, Ch. Des Montaneyres 4, 1443 Champvent, Switzerland*

[10]*Nanyang Technical University (NTU), 50 Nanyang Avenue, 639798 Singapore*

[$]These authors contributed equally.

*Corresponding authors:

christian-wolfrum@ethz.ch, current christian.wolfrum@ntu.edu.sg

jean-luc.wolfender@unige.ch

Source Data 2

This document contains NMR and HRMS spectra of constituents isolated in *Phaleria nisidai* extract, and complements the method details section. NMR spectra figures were prepared with the software. MNOVA 14. ThermoRAW HRMS data were converted to .mzXML using ProteoWizzard and loaded to MZmine 2.53 for figure preparation.

Contents

1. Iriflophenone 3-*C*-β-glucoside (1): 3
2. Mangiferin (2) 8
3. Iriflophenone-2-*O*-α-rhamnoside (3) 13
4. 2'-*O*-acetylmangiferin (4) 17
5. 2-*C*-β-glucofuranosylmangiferin (5) 22
6. Isovitexin (6) 27
7. 2-*C*-α-glucofuranosylmangiferin (7) 32
8. 6'-*O*-acetylmangiferin (8) 38
9. Genkwanin 5-*O*-β-primeveroside (9) 43
10. Genkwanin 5-*O*-β-glucoside (10) 48

1. Iriflophenone 3-*C*-β-glucoside (1):

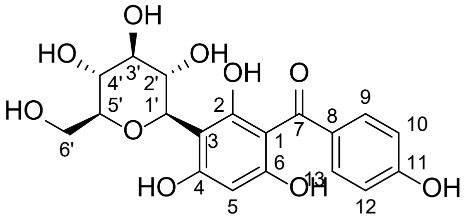

Chemical Formula: $C_{19}H_{20}O_{10}$

Exact Mass: 408.11

Supplementary Figure 2.1. Structure of iriflophenone 3-*C*-β-glucoside (**1**)

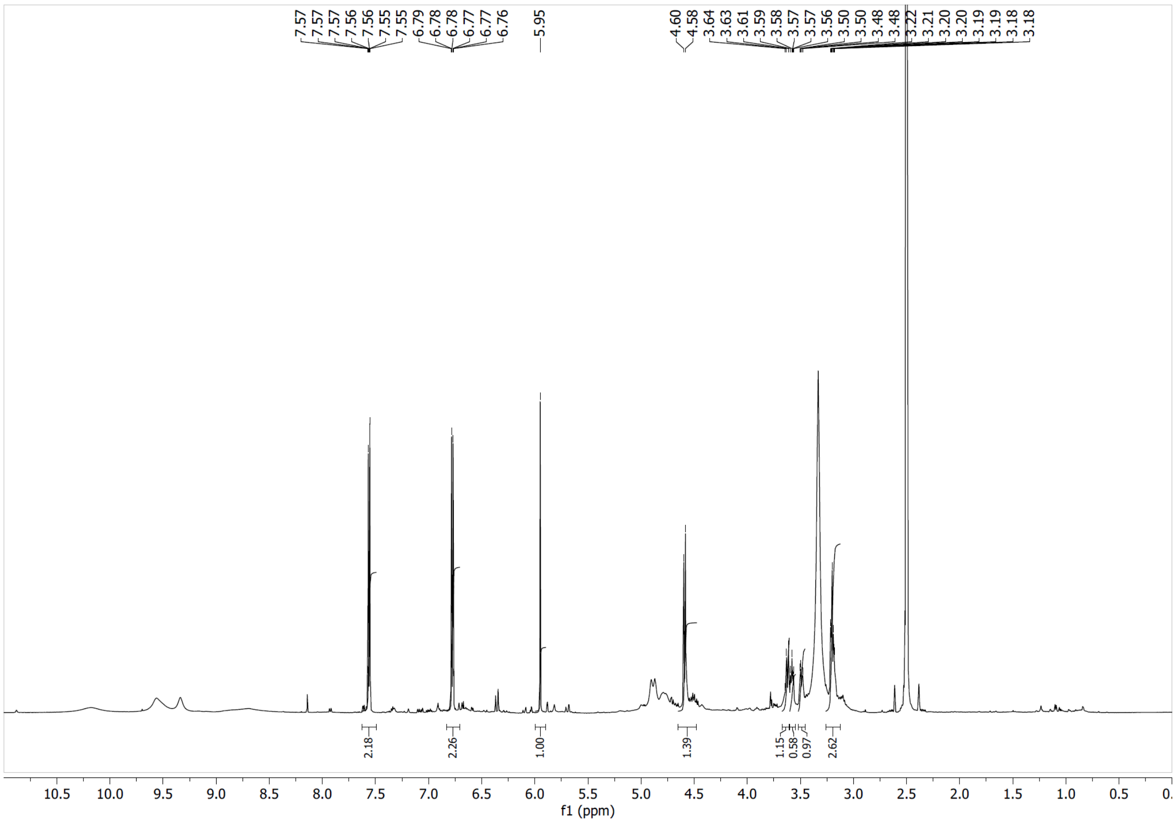

Supplementary Figure 2.2. ^{1}H NMR spectrum of iriflophenone 3-*C*-β-glucoside (**1**) in DMSO-d_6 at 600 MHz

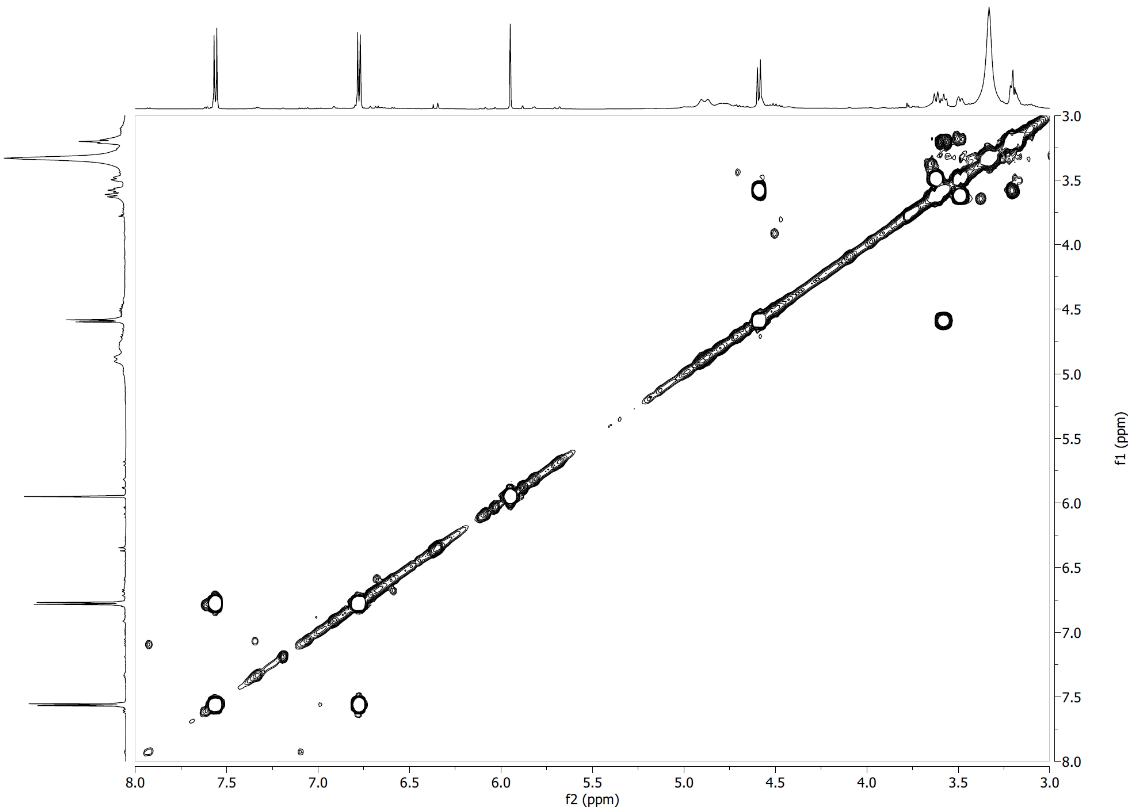

Supplementary Figure 2.3. COSY NMR spectrum of iriflophenone 3-*C*-β-glucoside (**1**) in DMSO-d_6

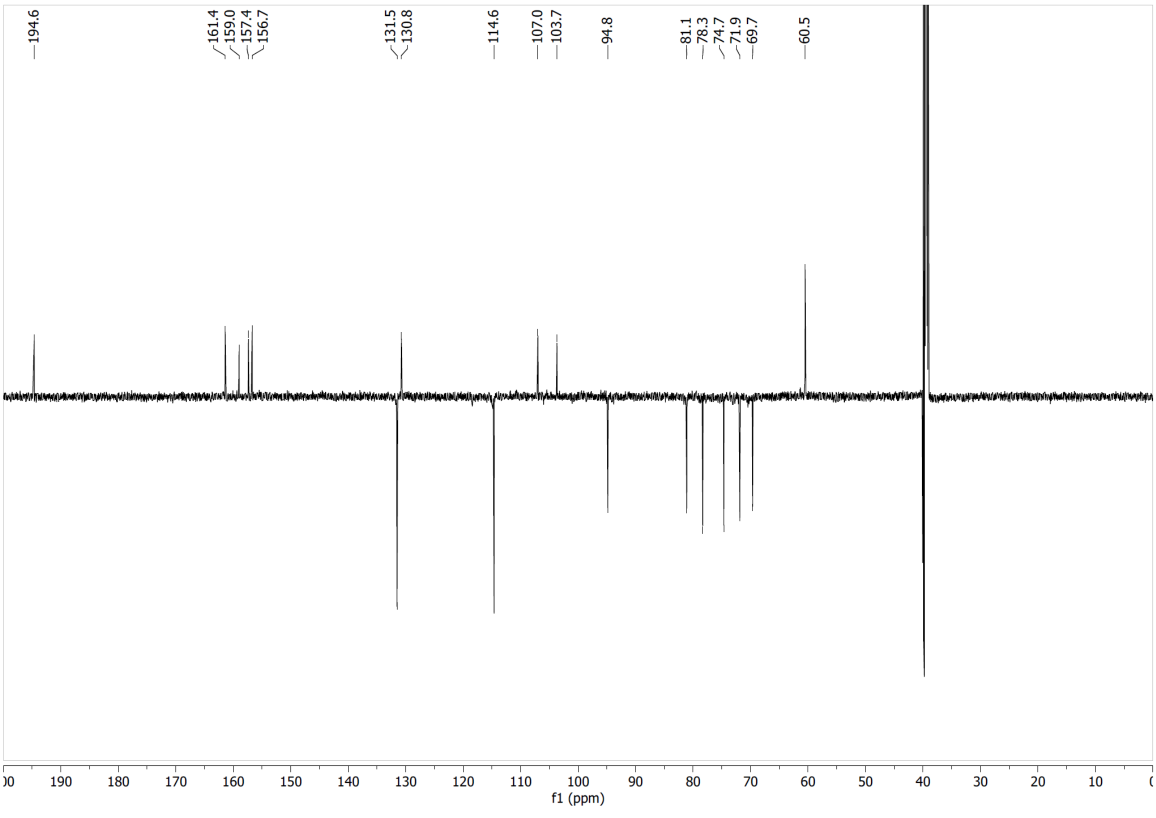

Supplementary Figure 2.4. ^{13}C-DEPTQ NMR spectrum of iriflophenone 3-*C*-β-glucoside (**1**) in DMSO-d_6 at 151 MHz

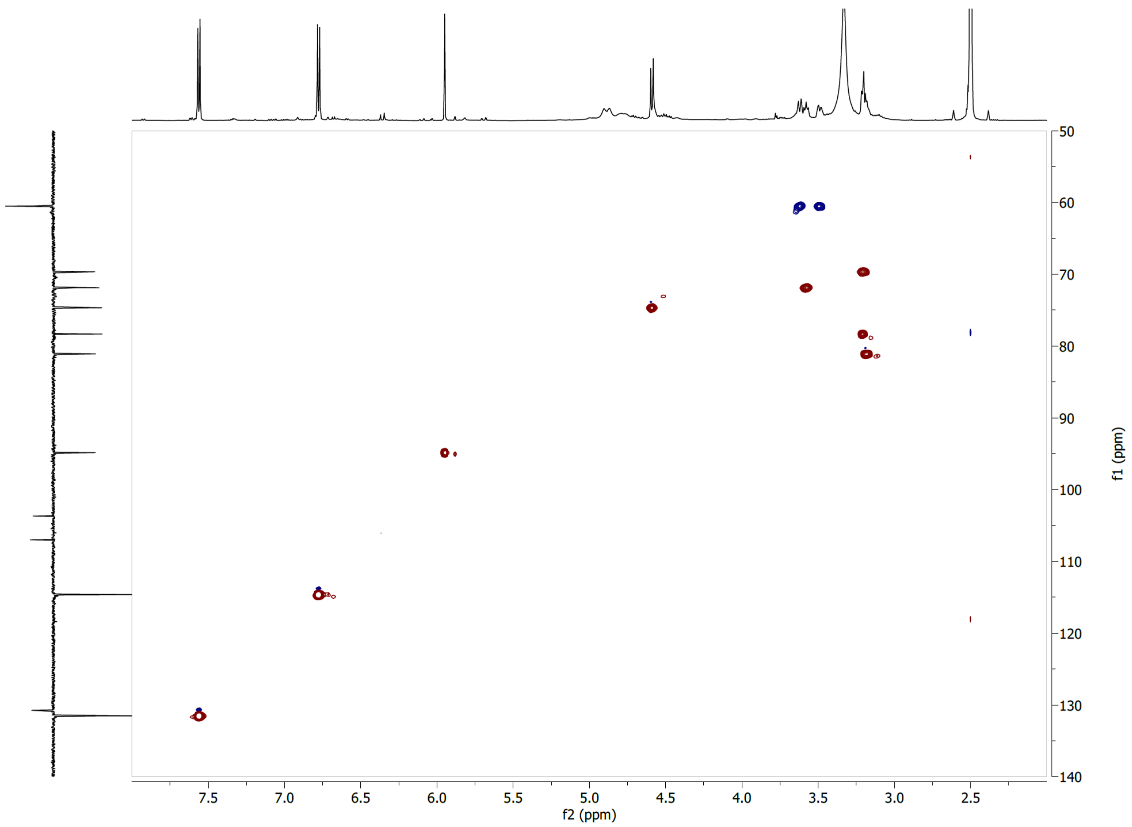

Supplementary Figure 2.5. Edited-HSQC NMR spectrum of iriflophenone 3-*C*-β-glucoside (**1**) in DMSO-d_6

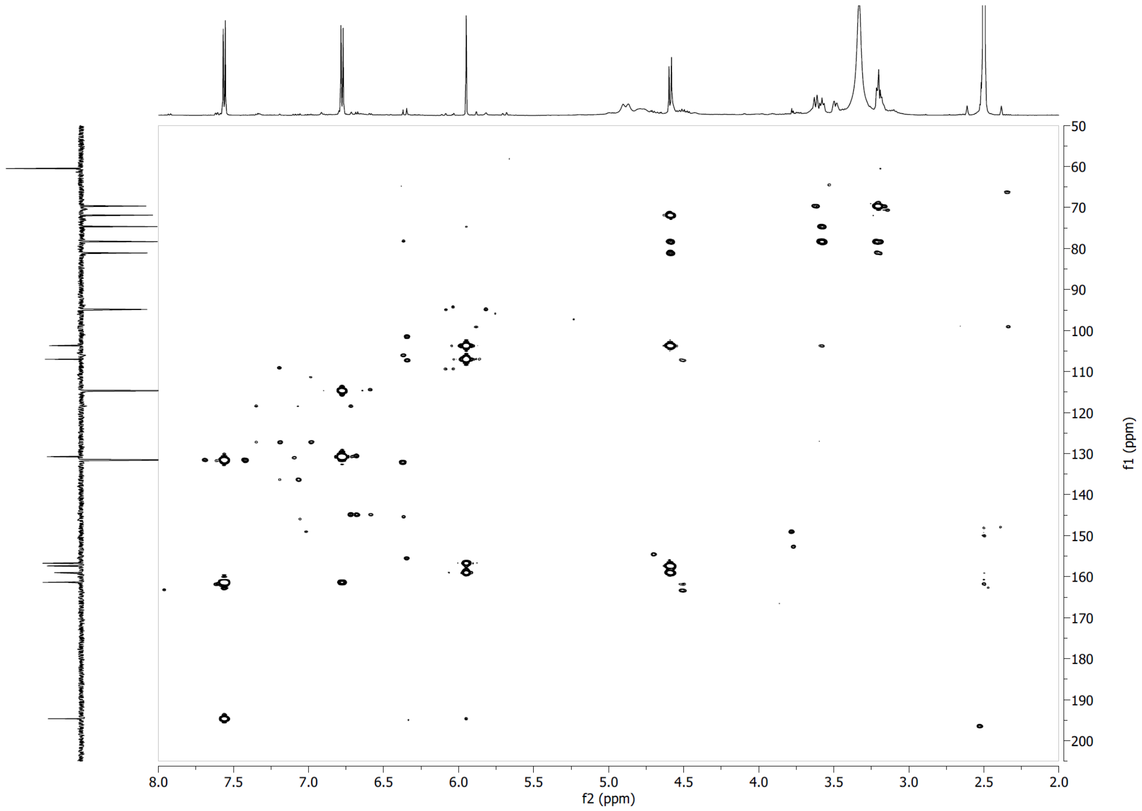

Supplementary Figure 2.6. HMBC NMR spectrum of iriflophenone 3-*C*-β-glucoside (**1**) in DMSO-d_6

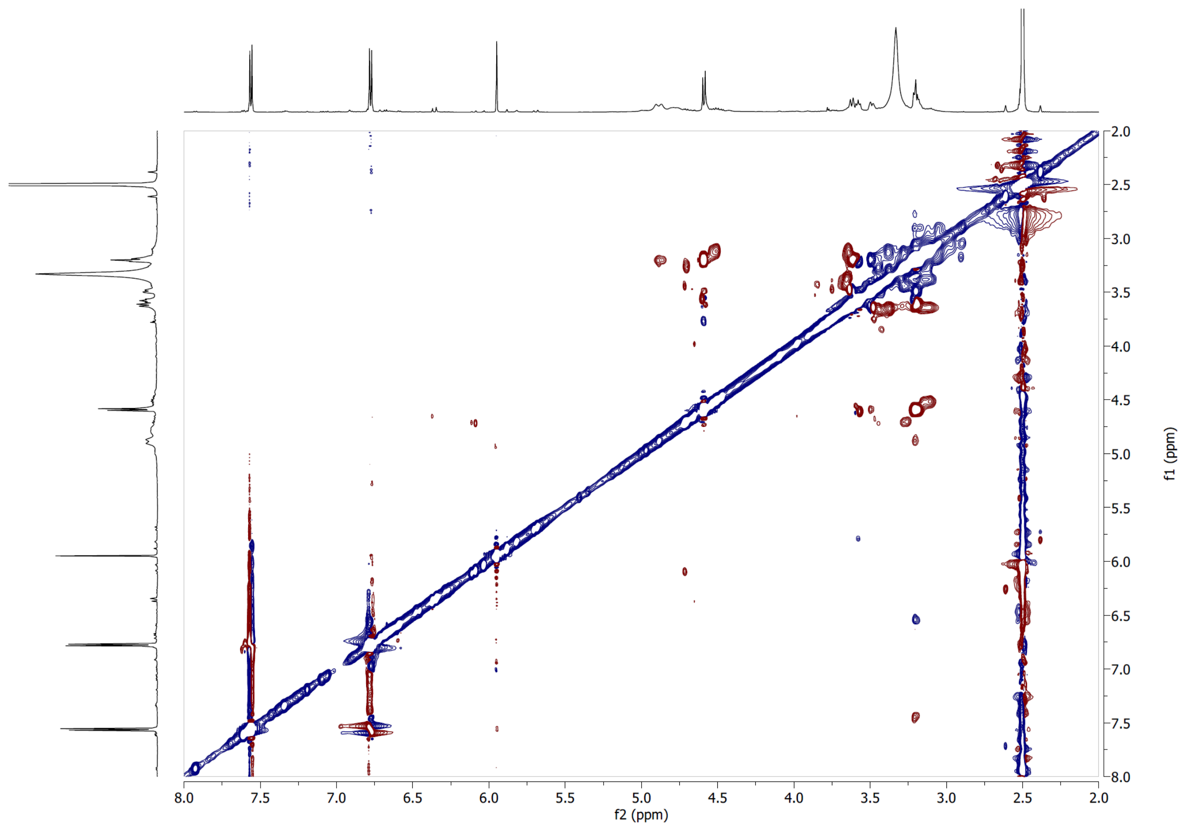

Supplementary Figure 2.7. ROESY NMR spectrum of iriflophenone 3-*C*-β-glucoside (**1**) in DMSO-d_6

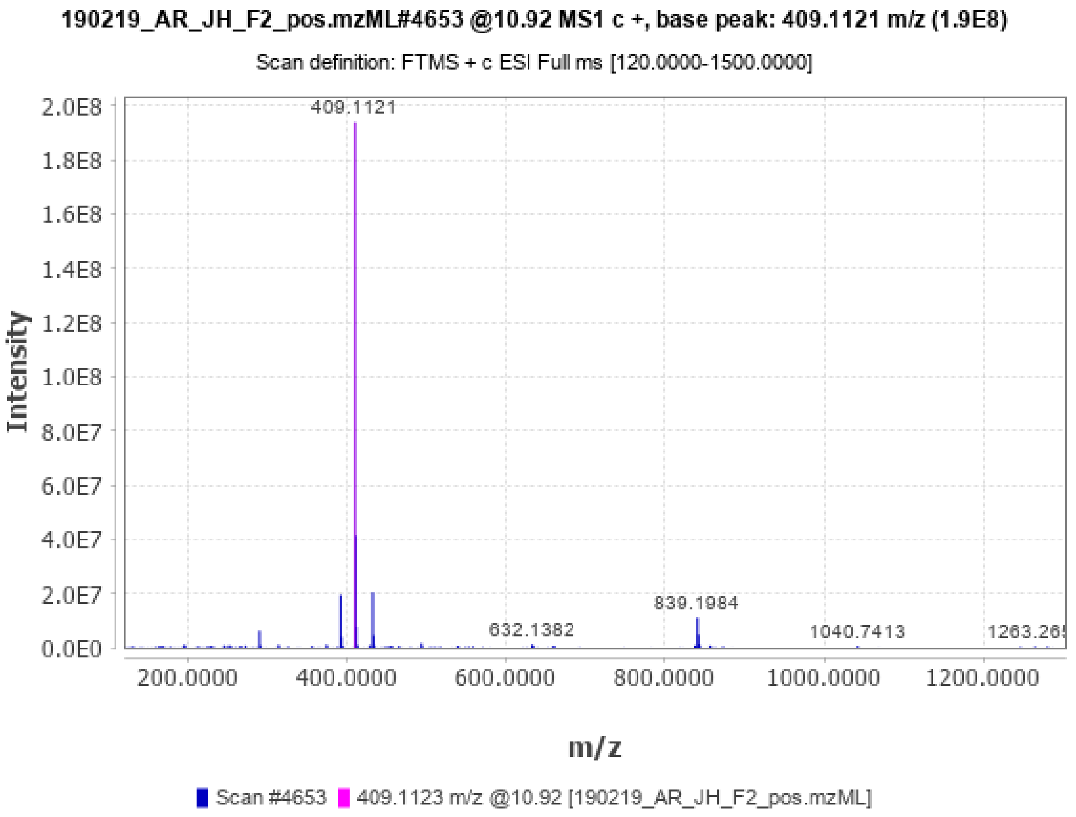

Supplementary Figure 2.8. HRMS spectrum of iriflophenone 3-*C*-β-glucoside (**1**) ([M+H]$^+$) in Fraction 2 obtained by UHPLC-HRMS in positive ionization.

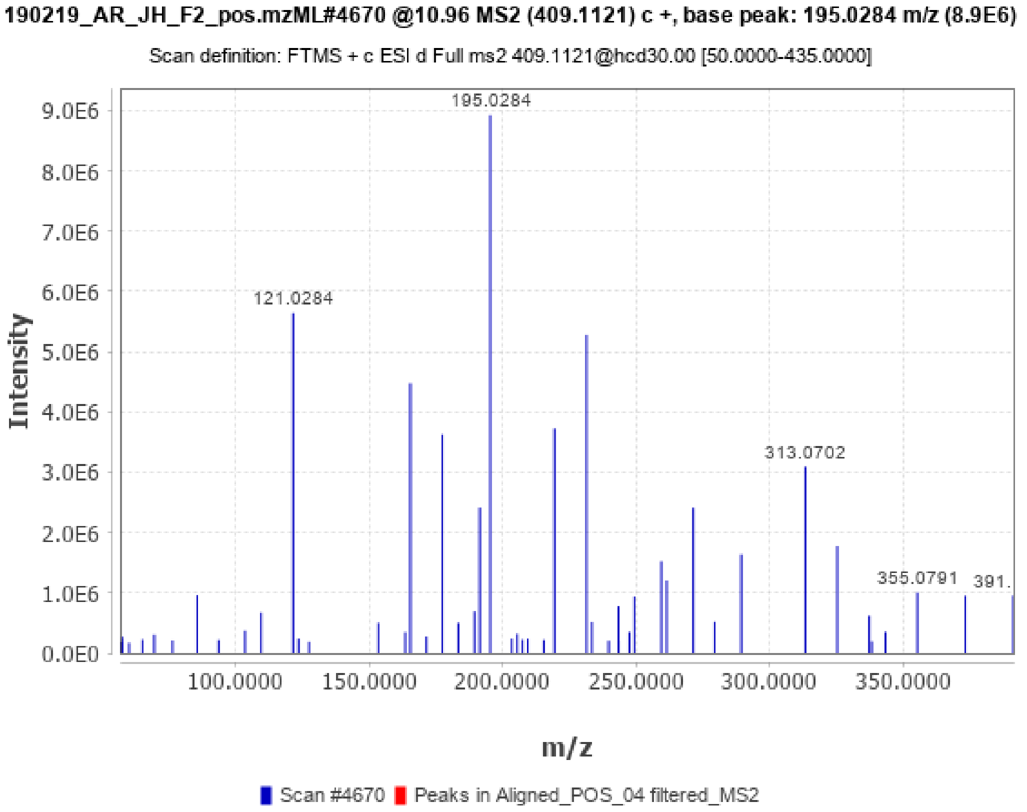

Supplementary Figure 2.9. Fragmentation spectrum of iriflophenone 3-*C*-β-glucoside (**1**) ([M+H]$^+$) in Fraction 2 obtained by UHPLC-HRMS in positive ionization.

2. Mangiferin (2)

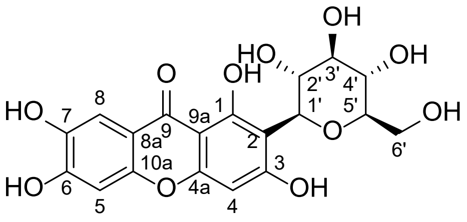

Chemical Formula: $C_{19}H_{18}O_{11}$
Exact Mass: 422.08

Supplementary Figure 2.10. Structure of mangiferin (**2**)

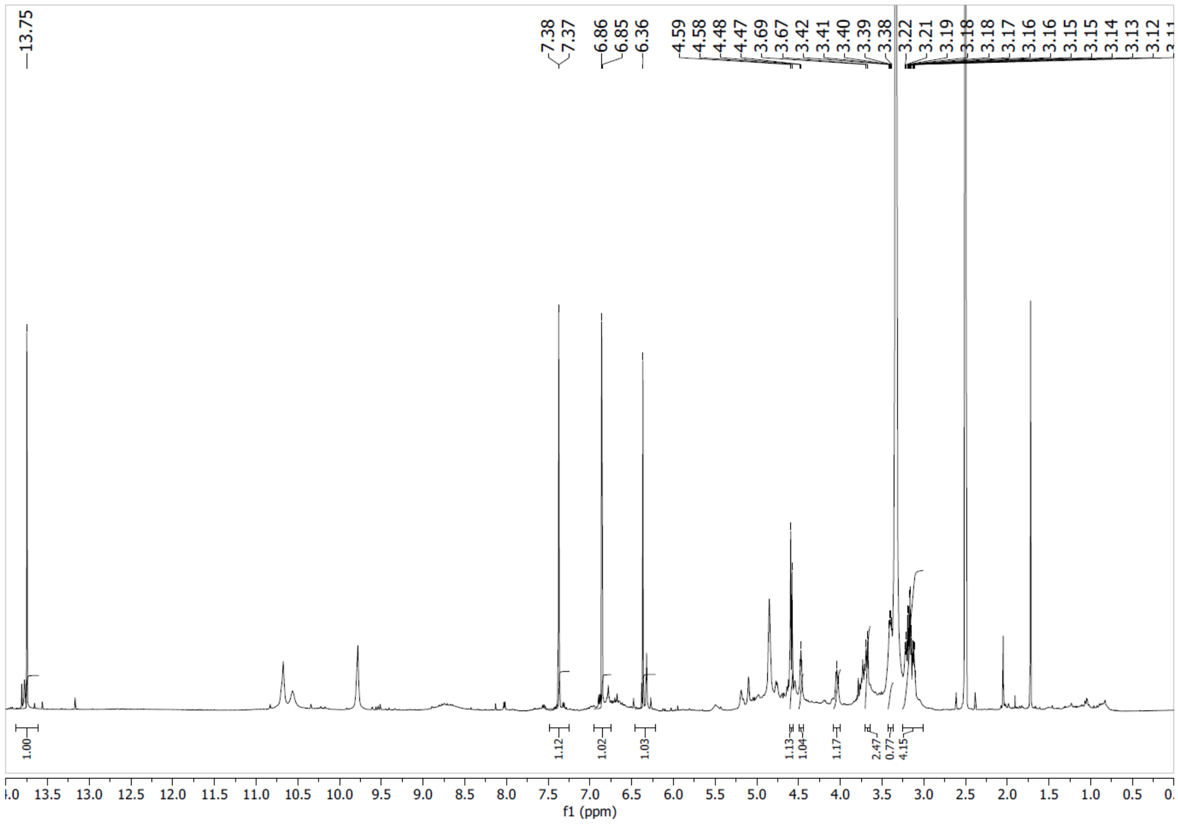

Supplementary Figure 2.11. ^{1}H NMR spectrum of mangiferin (**2**) in DMSO-d_6 at 600 MHz

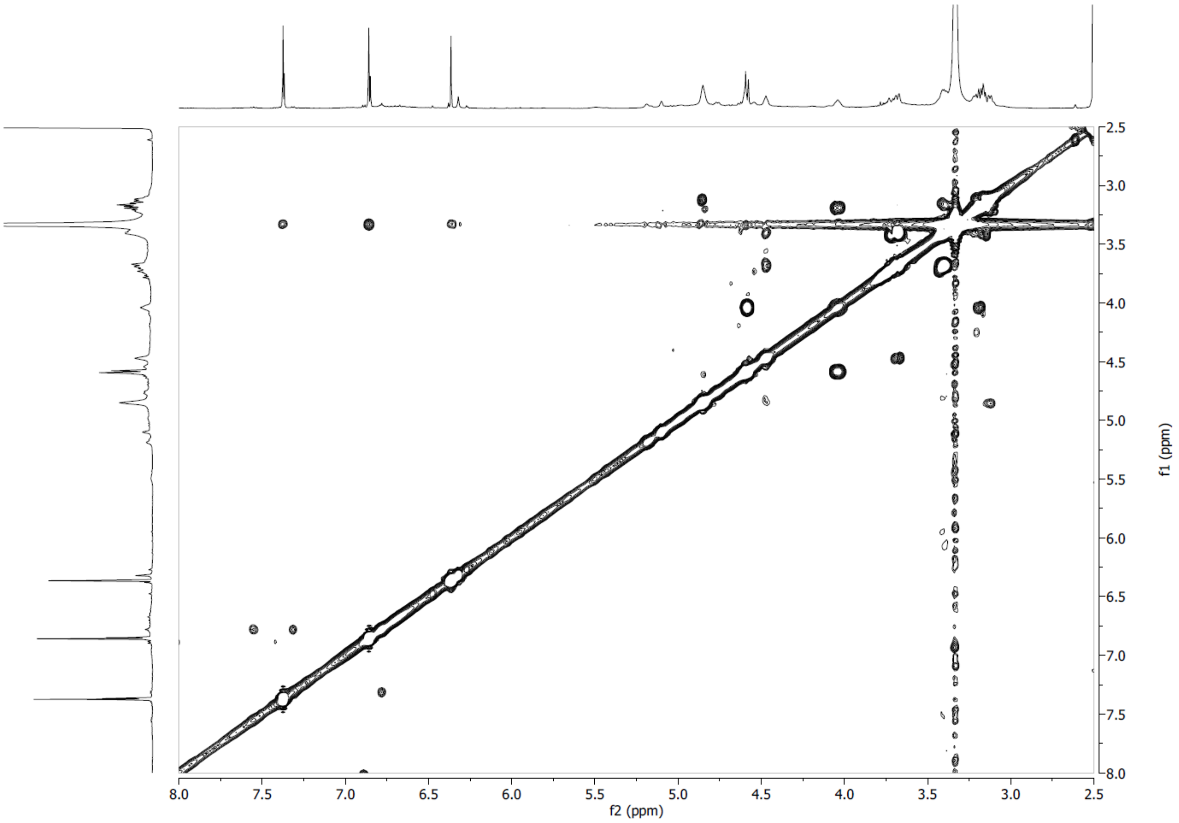

Supplementary Figure 2.12. COSY NMR spectrum of mangiferin (**2**) in DMSO-*d*6

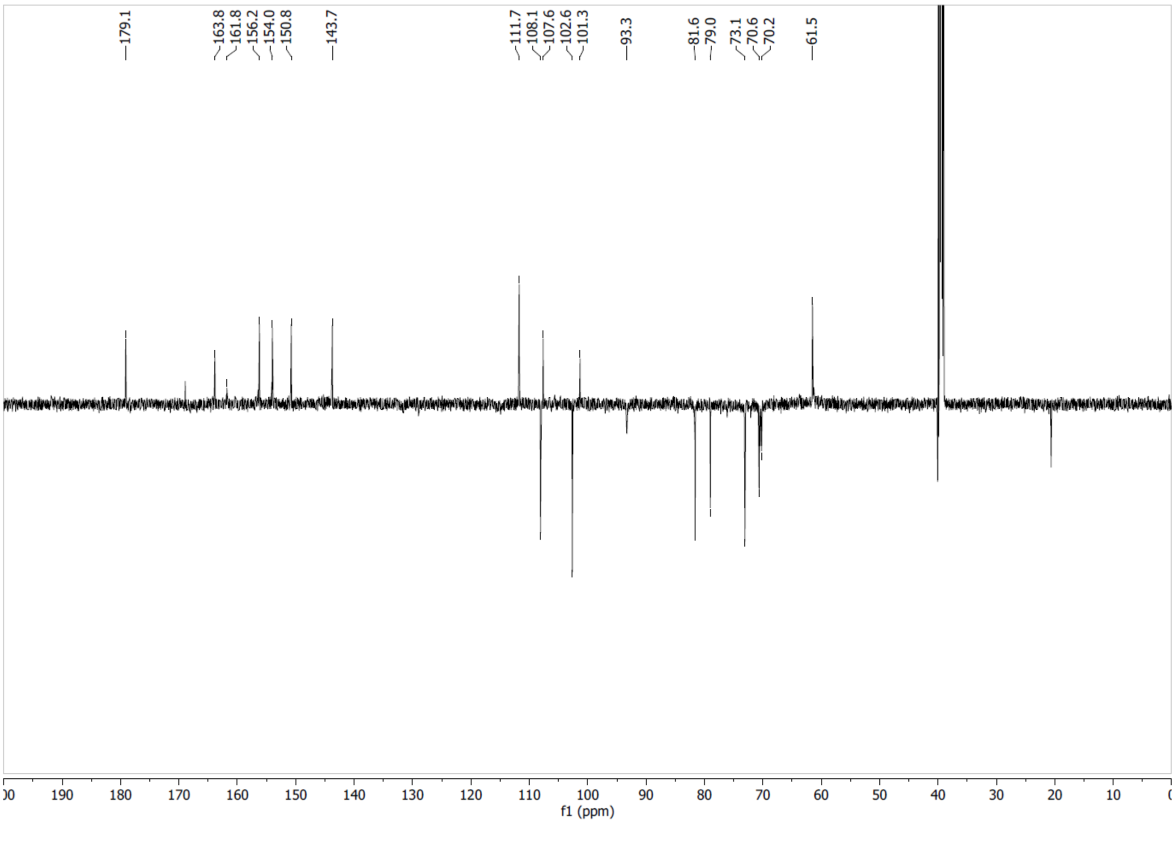

Supplementary Figure 2.13. ^{13}C-DEPTQ NMR spectrum of mangiferin (**2**) in DMSO-*d*6 at 151 MHz

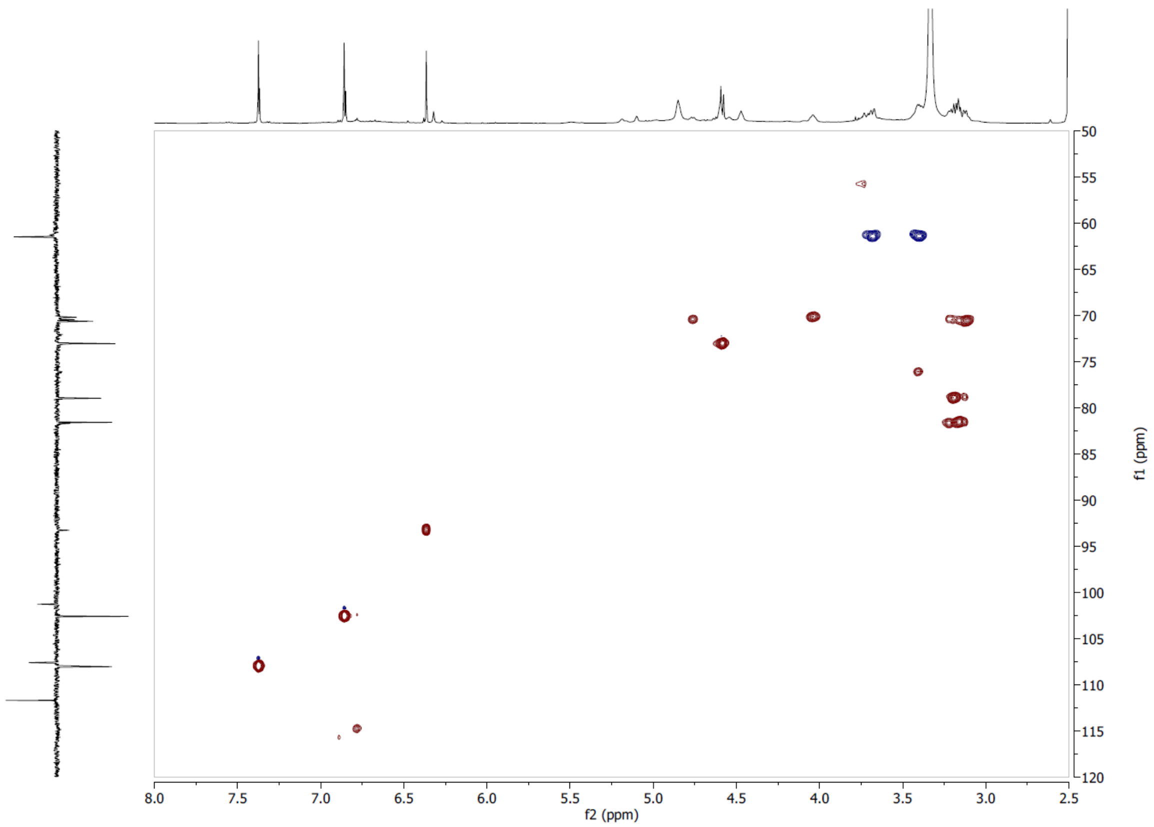

Supplementary Figure 2.14. Edited-HSQC NMR spectrum of mangiferin (**2**) in DMSO-*d*6

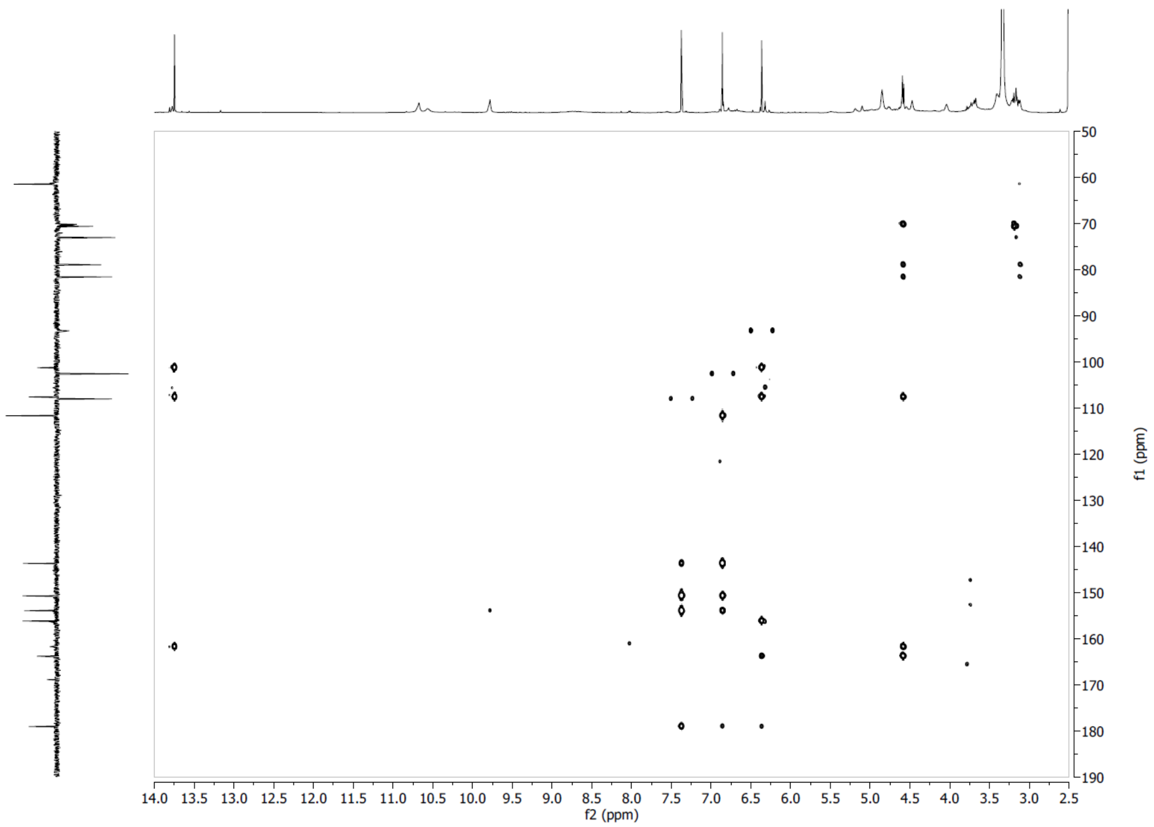

Supplementary Figure 2.15. HMBC NMR spectrum of mangiferin (**2**) in DMSO-*d*6

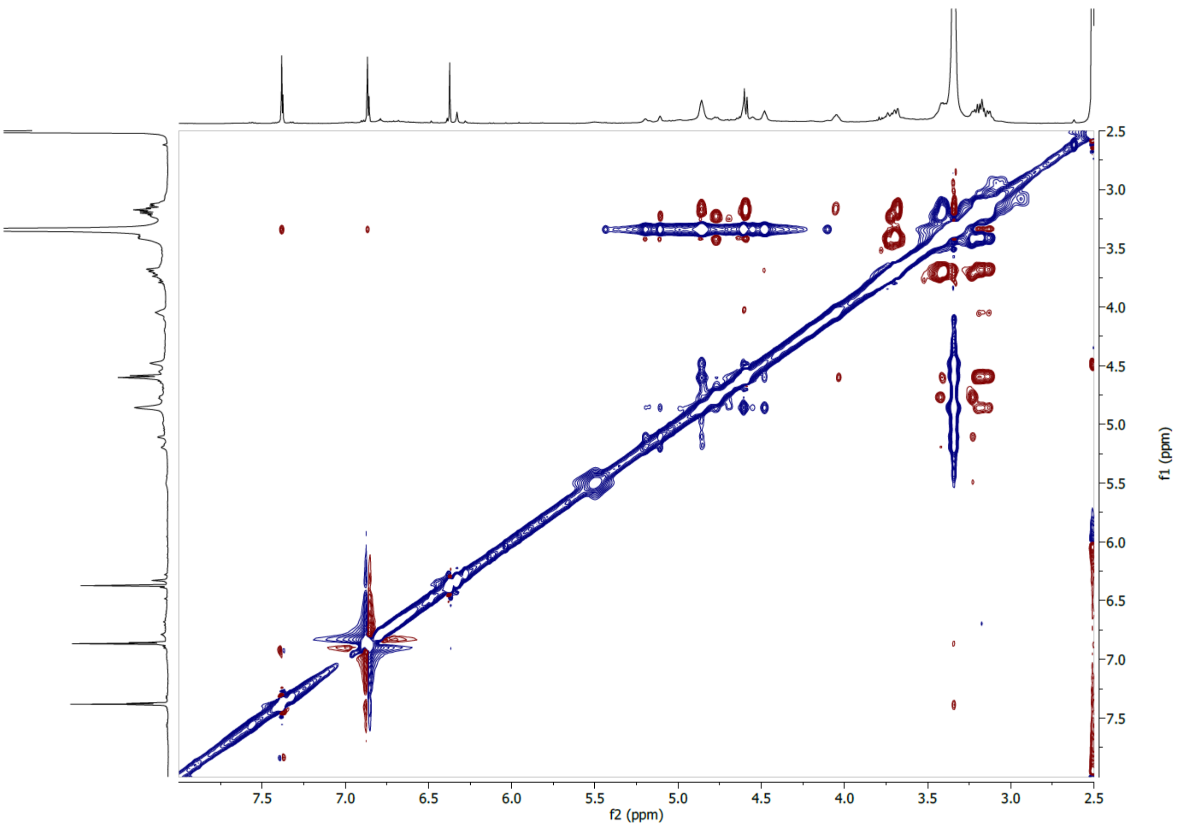

Supplementary Figure 2.16. ROESY NMR spectrum of mangiferin (**2**) in DMSO-d_6

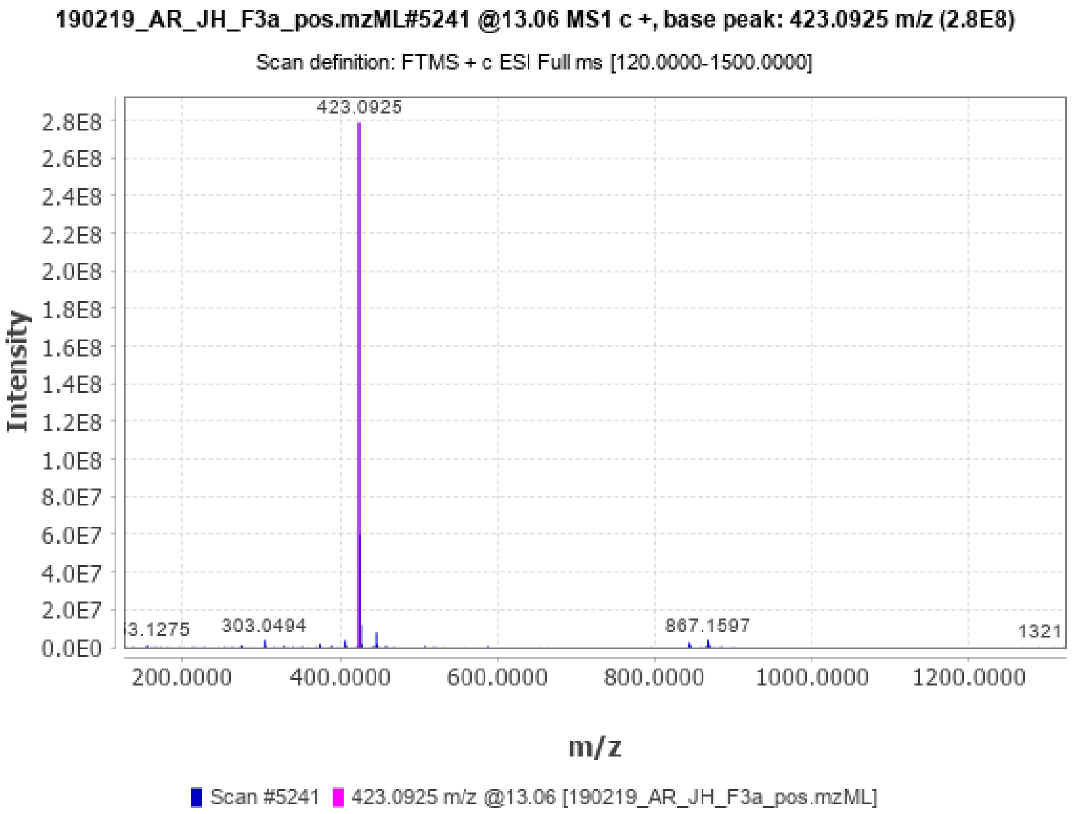

Supplementary Figure 2.17. HRMS spectrum of mangiferin (2) ($[M+H]^+$) in Fraction 3 obtained by UHPLC-HRMS in positive ionization.

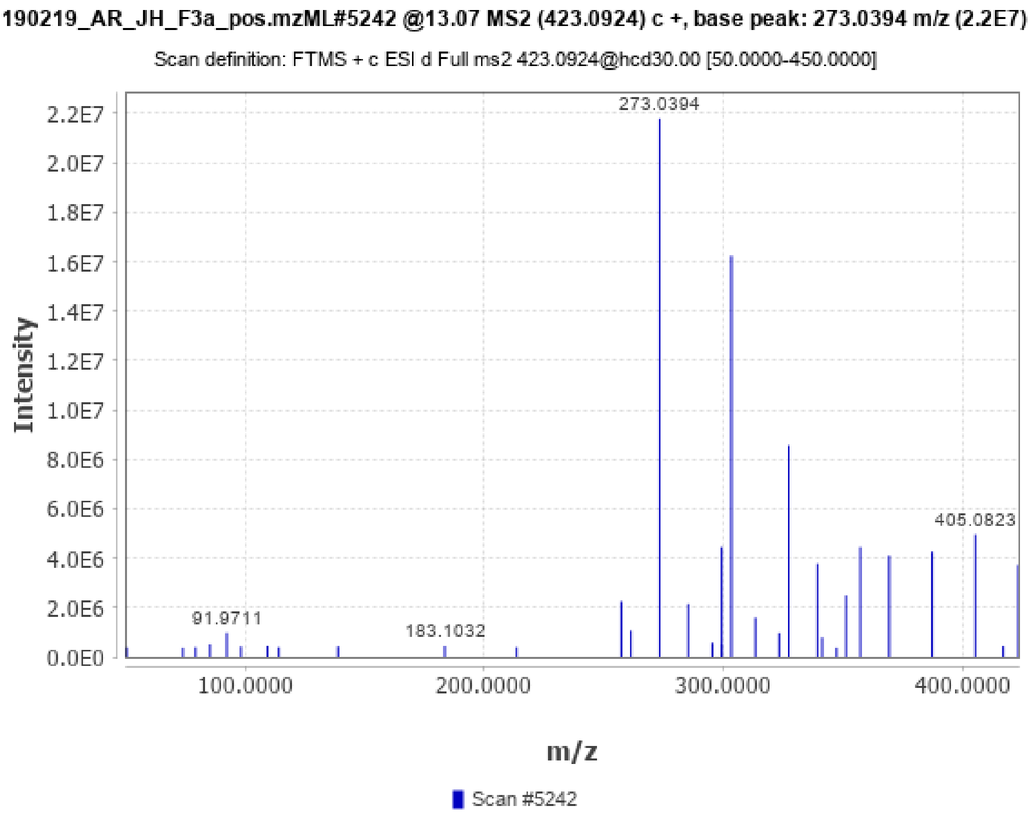

Supplementary Figure 2.18. Fragmentation spectrum of mangiferin (2) ($[M+H]^+$) in Fraction 3 obtained by UHPLC-HRMS in positive ionization.

3. Iriflophenone-2-*O*-α-rhamnoside (3)

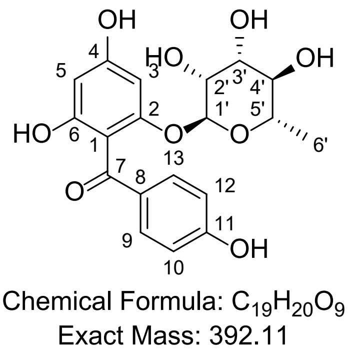

Supplementary Figure 2.19. Structure of iriflophenone-2-*O*-α-rhamnoside (**3**)

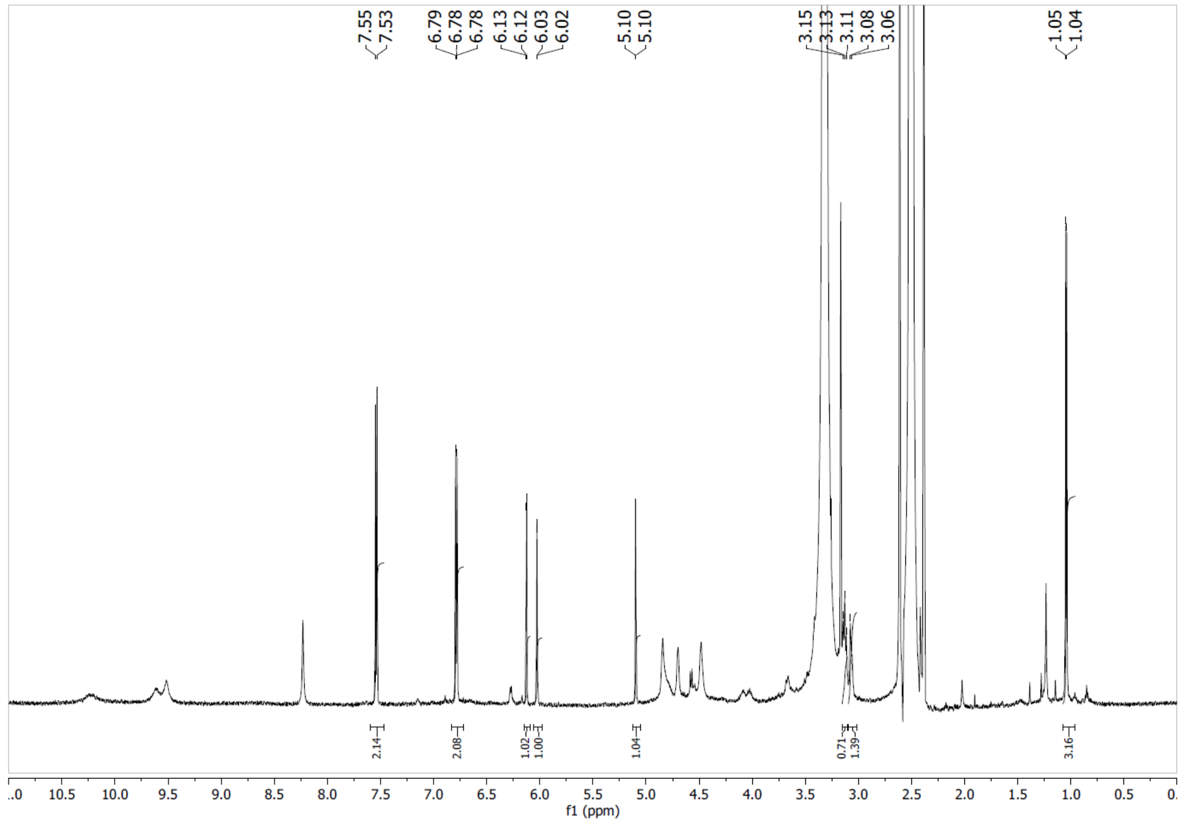

Supplementary Figure 2.20. ^{1}H NMR spectrum of iriflophenone-2-*O*-α-rhamnoside (**3**) in DMSO-d_6 at 600 MHz

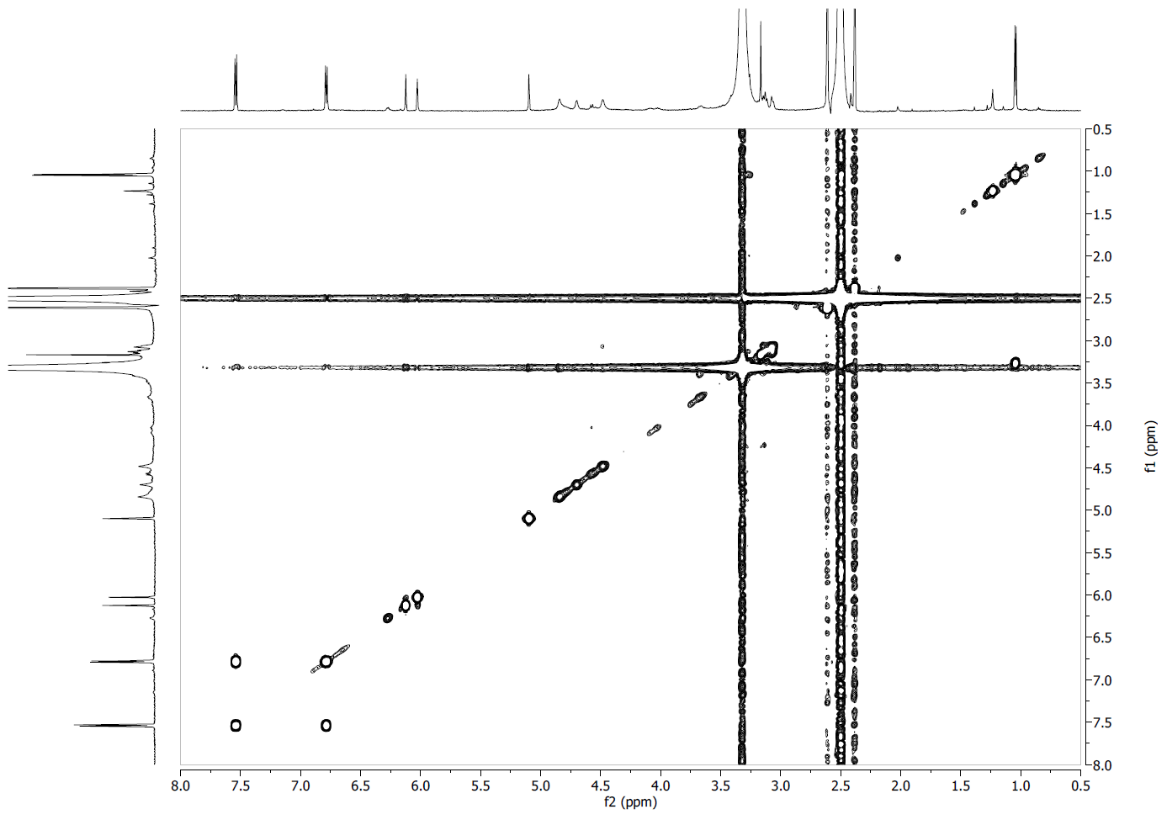

Supplementary Figure 2.21. COSY NMR spectrum of iriflophenone-2-*O*-α-rhamnoside (**3**) in DMSO-d_6

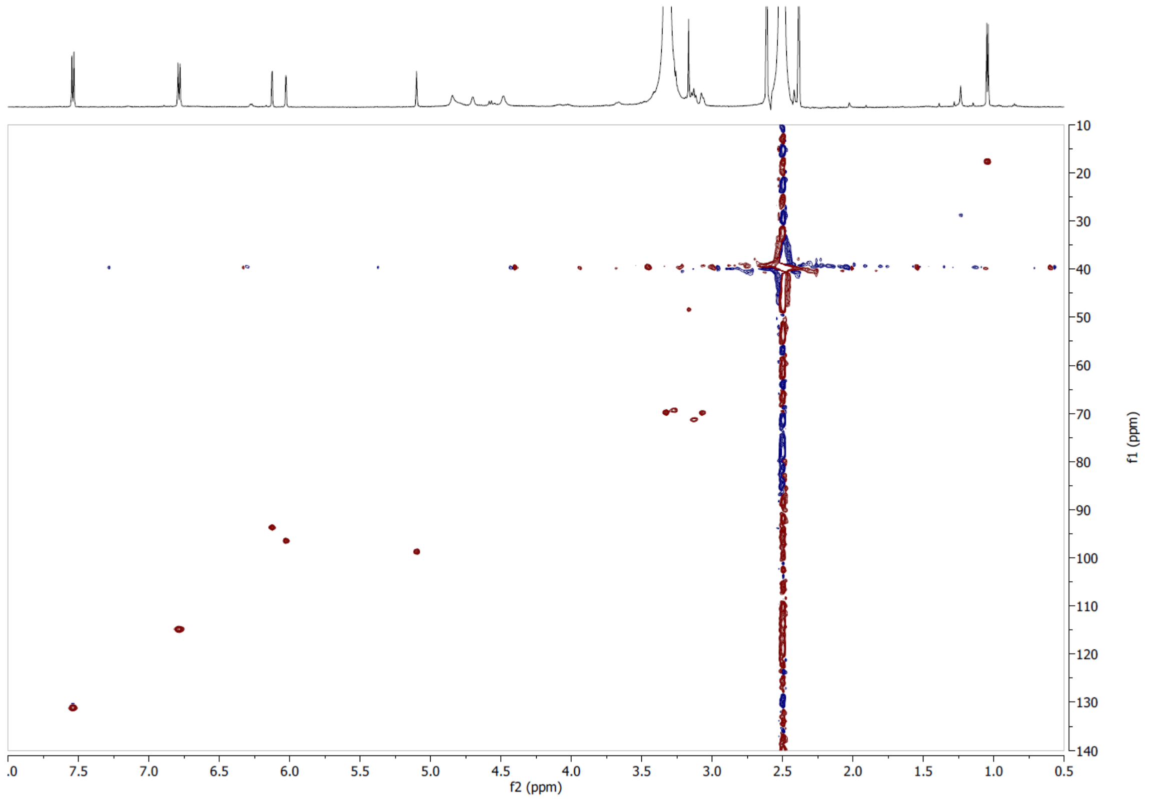

Supplementary Figure 2.22. Edited-HSQC NMR spectrum of iriflophenone-2-*O*-α-rhamnoside (**3**) in DMSO-d6

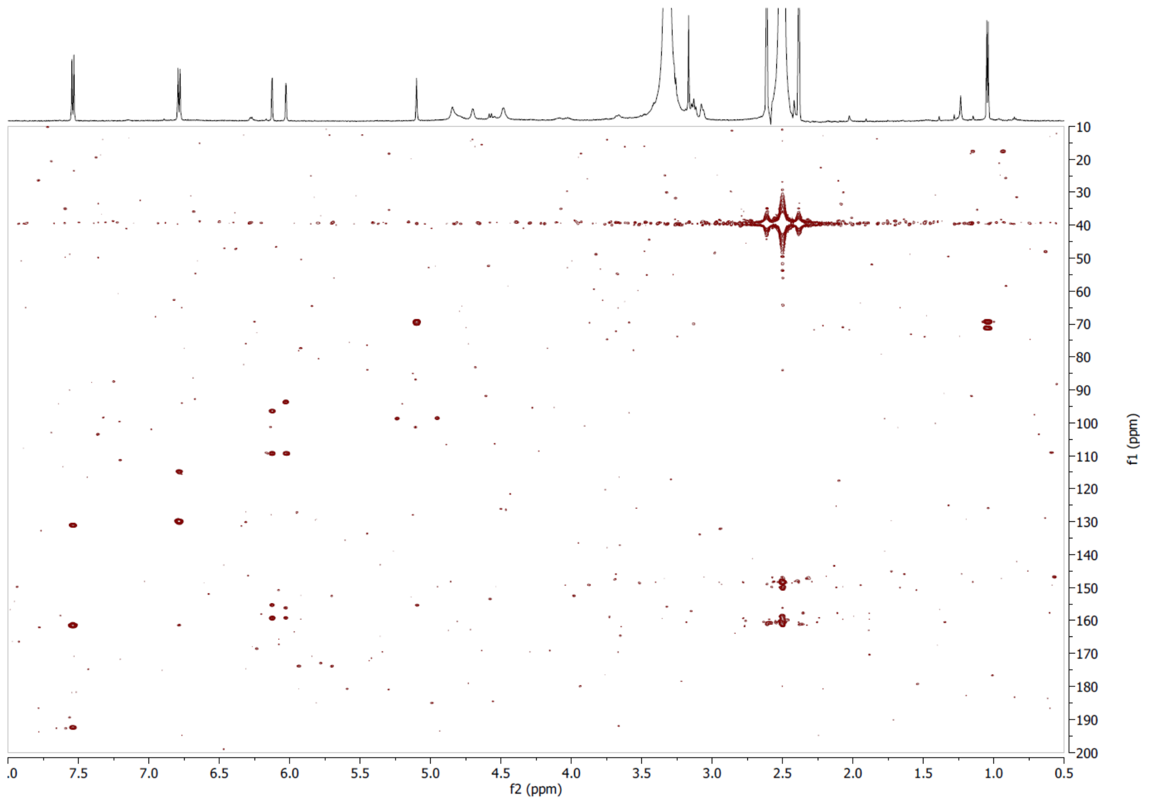

Supplementary Figure 2.23. HMBC NMR spectrum of iriflophenone-2-*O*-α-rhamnoside (**3**) in DMSO-d_6

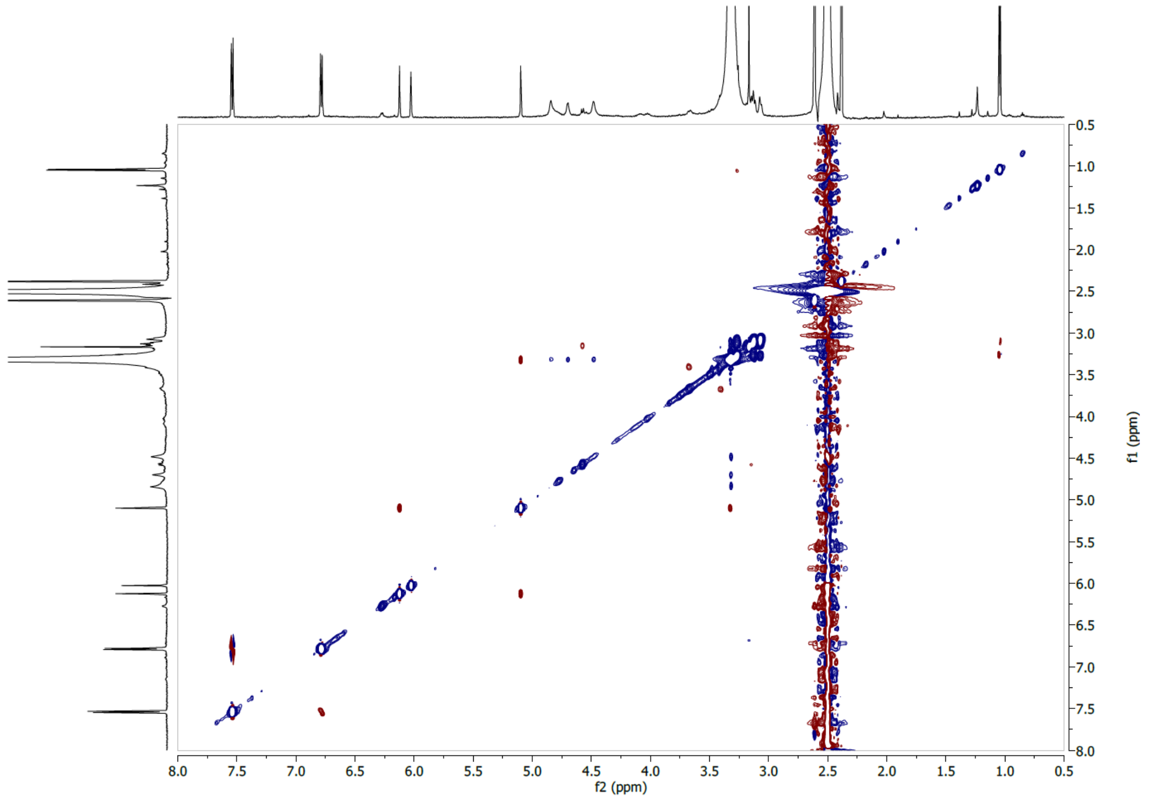

Supplementary Figure 2.24. ROESY NMR spectrum of iriflophenone-2-*O*-α-rhamnoside (**3**) in DMSO-d_6

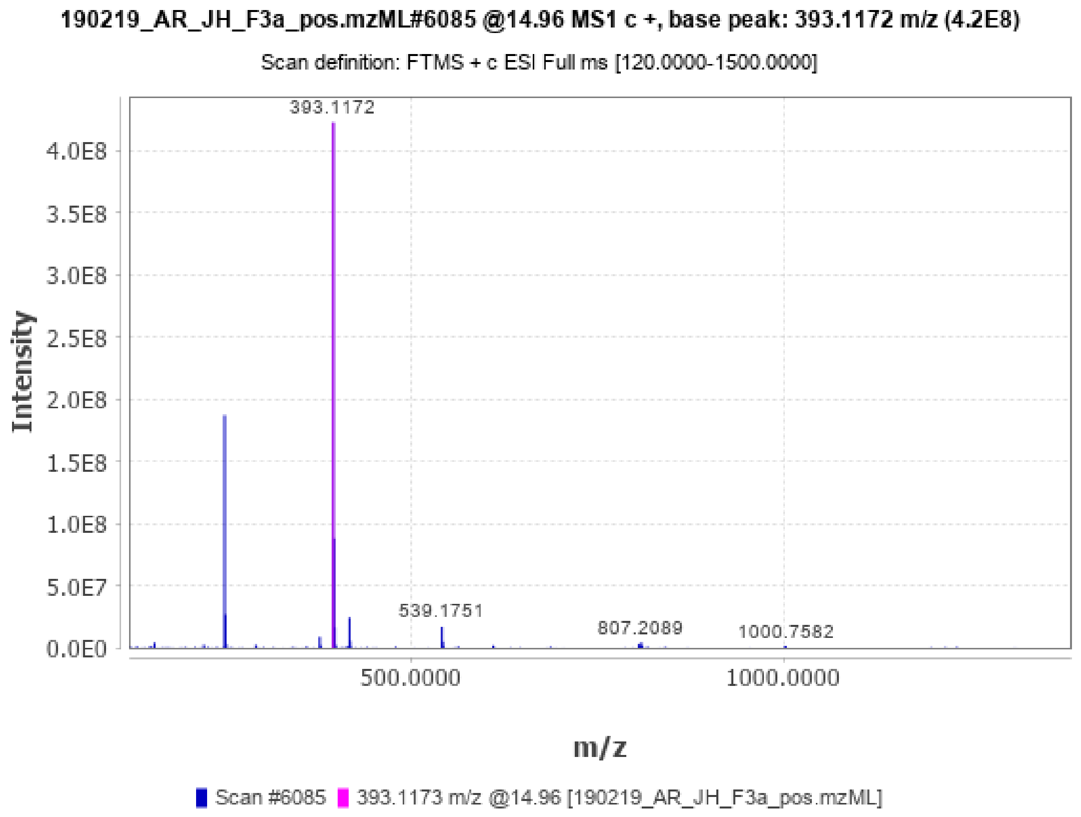

Supplementary Figure 2.25. HRMS spectrum of iriflophenone-2-*O*-α-rhamnoside (**3**) ([M+H]$^+$) in Fraction 3 obtained by UHPLC-HRMS in positive ionization.

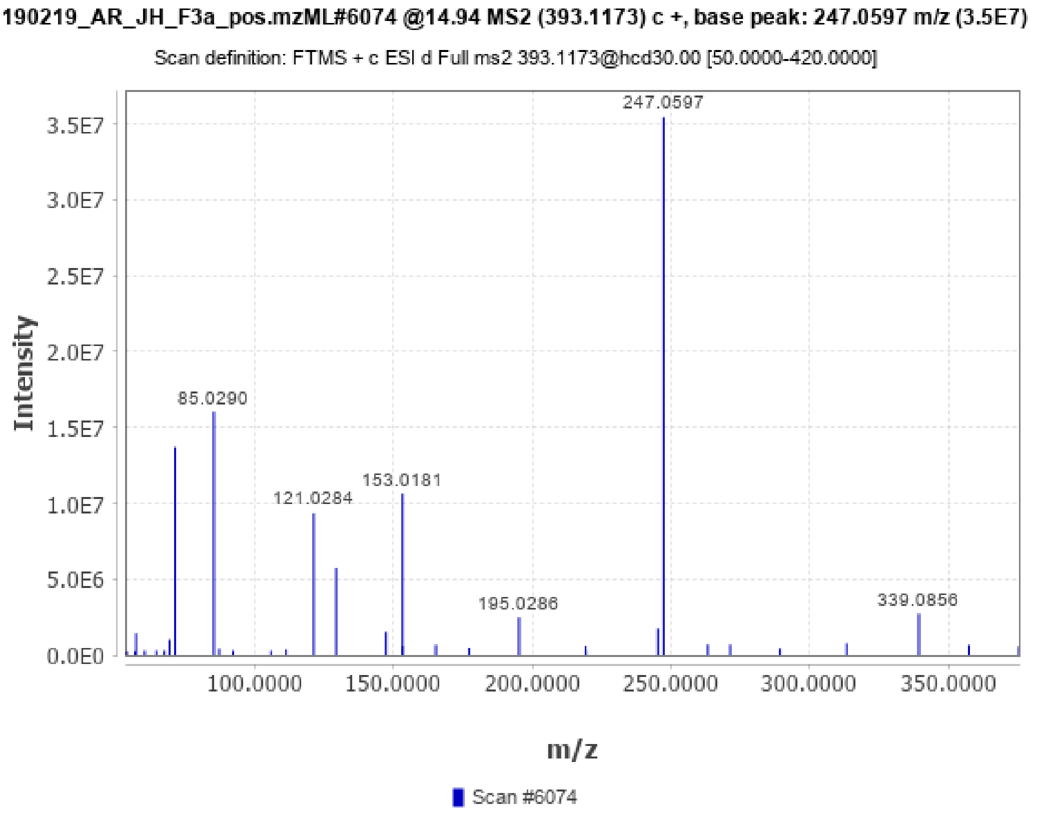

Supplementary Figure 2.26. Fragmentation spectrum of iriflophenone-2-*O*-α-rhamnoside (**3**) ([M+H]$^+$) in Fraction 3 obtained by UHPLC-HRMS in positive ionization.

4. 2'-*O*-acetylmangiferin (4)

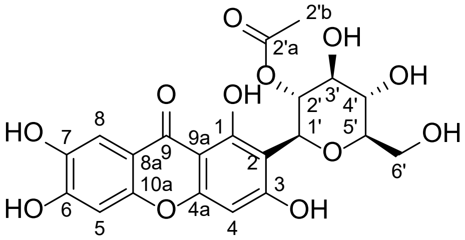

Chemical Formula: $C_{21}H_{20}O_{12}$
Exact Mass: 464.10

Supplementary Figure 2.27. Structure of 2'-*O*-acetylmangiferin (**4**)

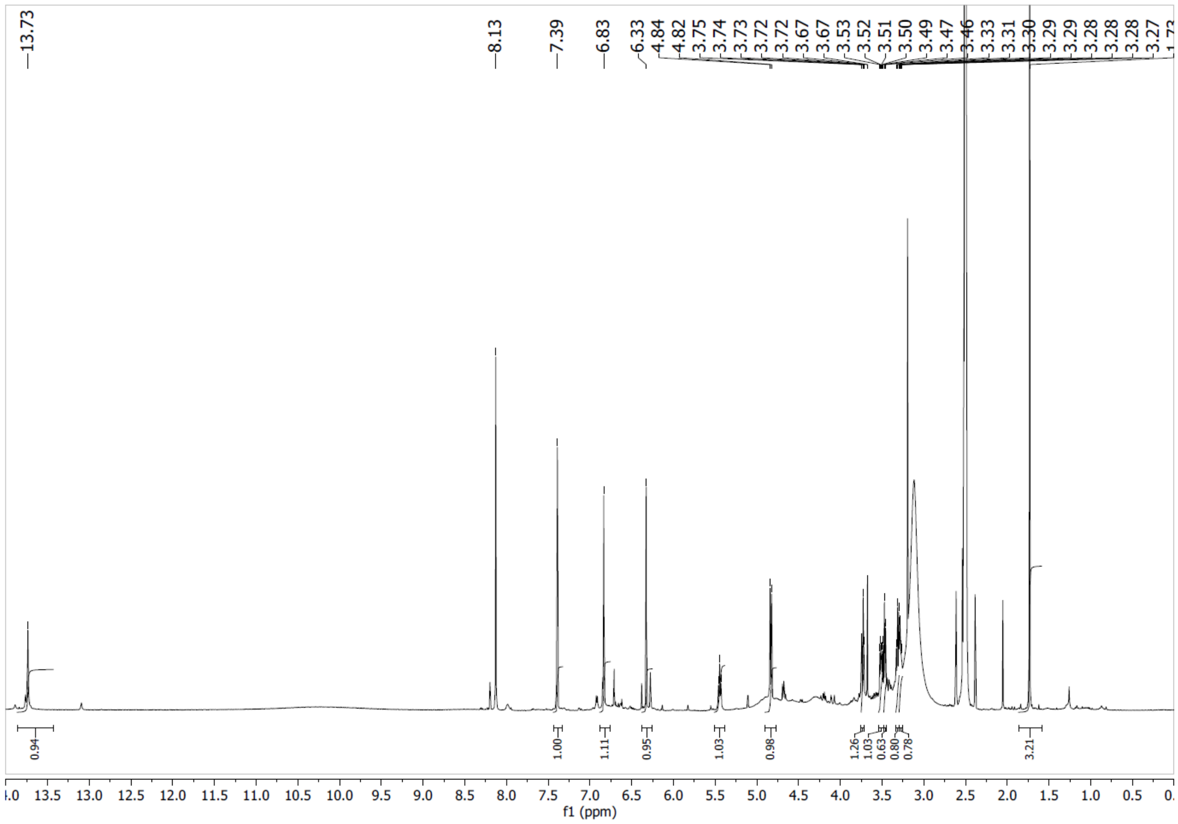

Supplementary Figure 2.28. ¹H NMR spectrum of 2'-*O*-acetylmangiferin (**4**) in DMSO-d_6 at 600 MHz and T=343K

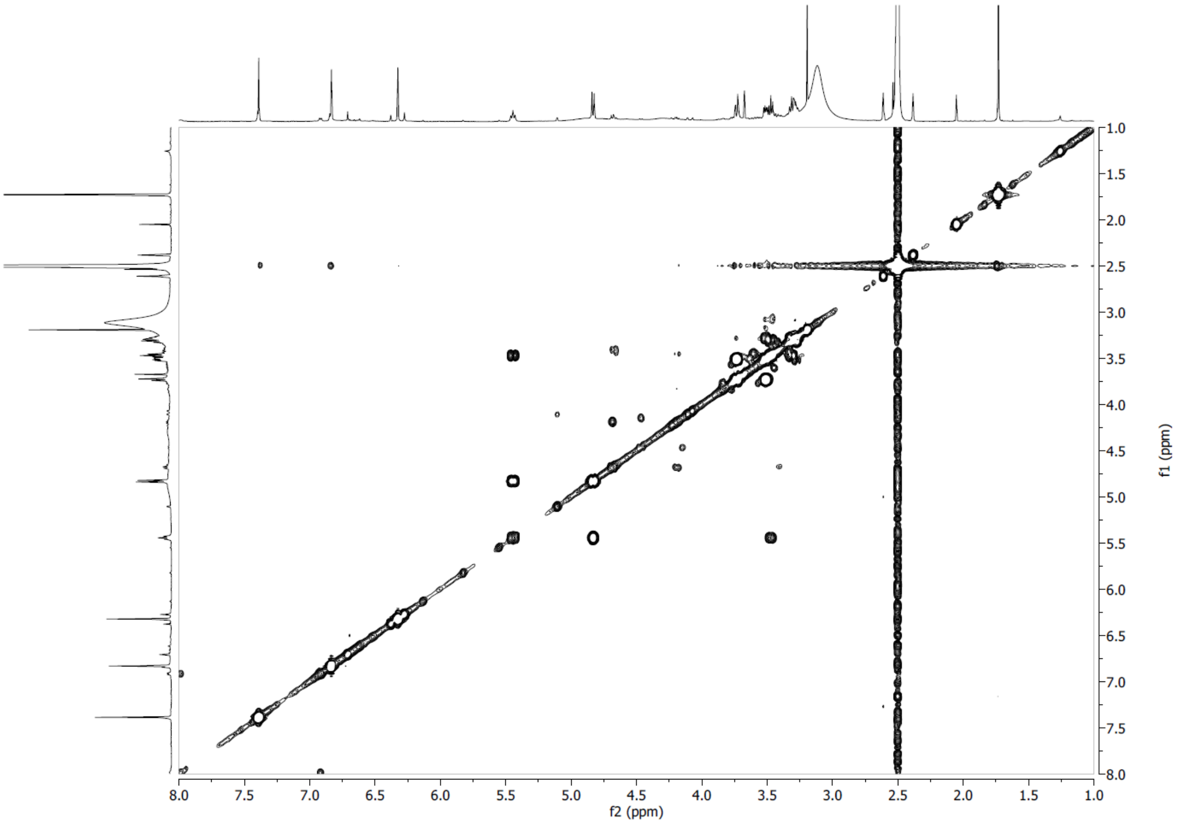

Supplementary Figure 2.29. COSY NMR spectrum of 2'-*O*-acetylmangiferin (**4**) in DMSO-d_6 and T=343K

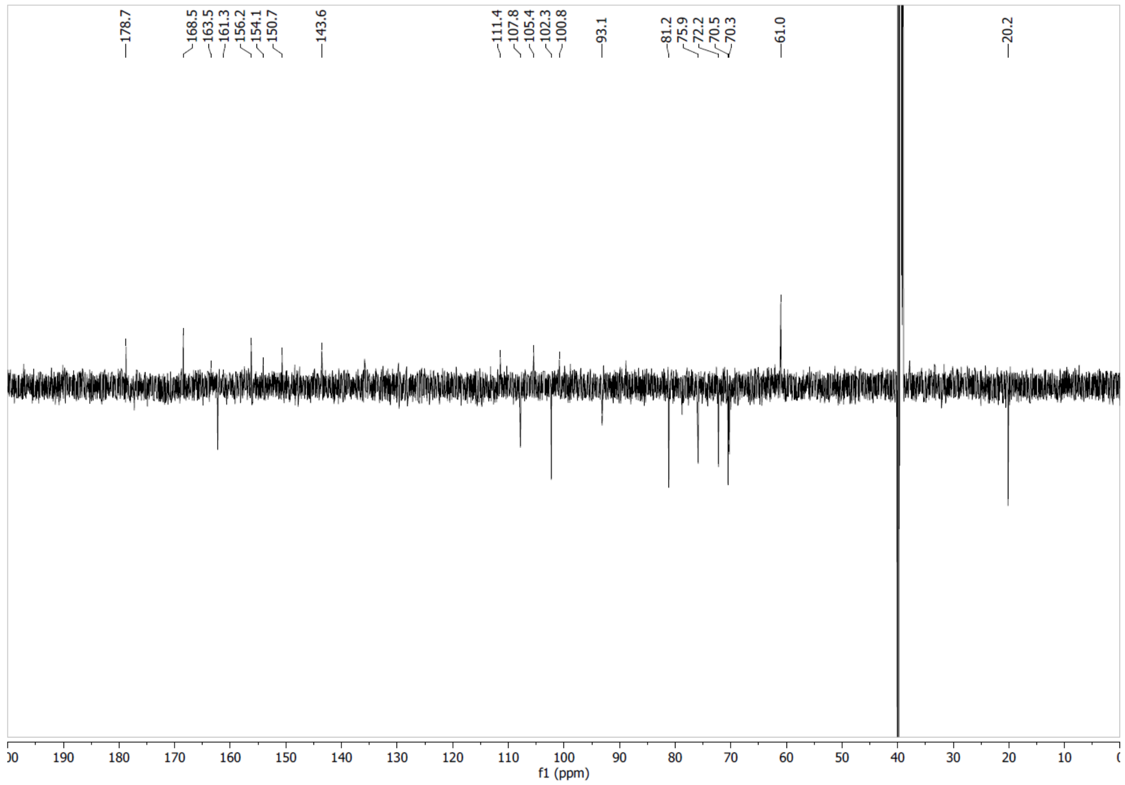

Supplementary Figure 2.30. ^{13}C-DEPTQ NMR spectrum of 2'-*O*-acetylmangiferin (**4**) in DMSO-d_6 at 151 MHz and T=343K

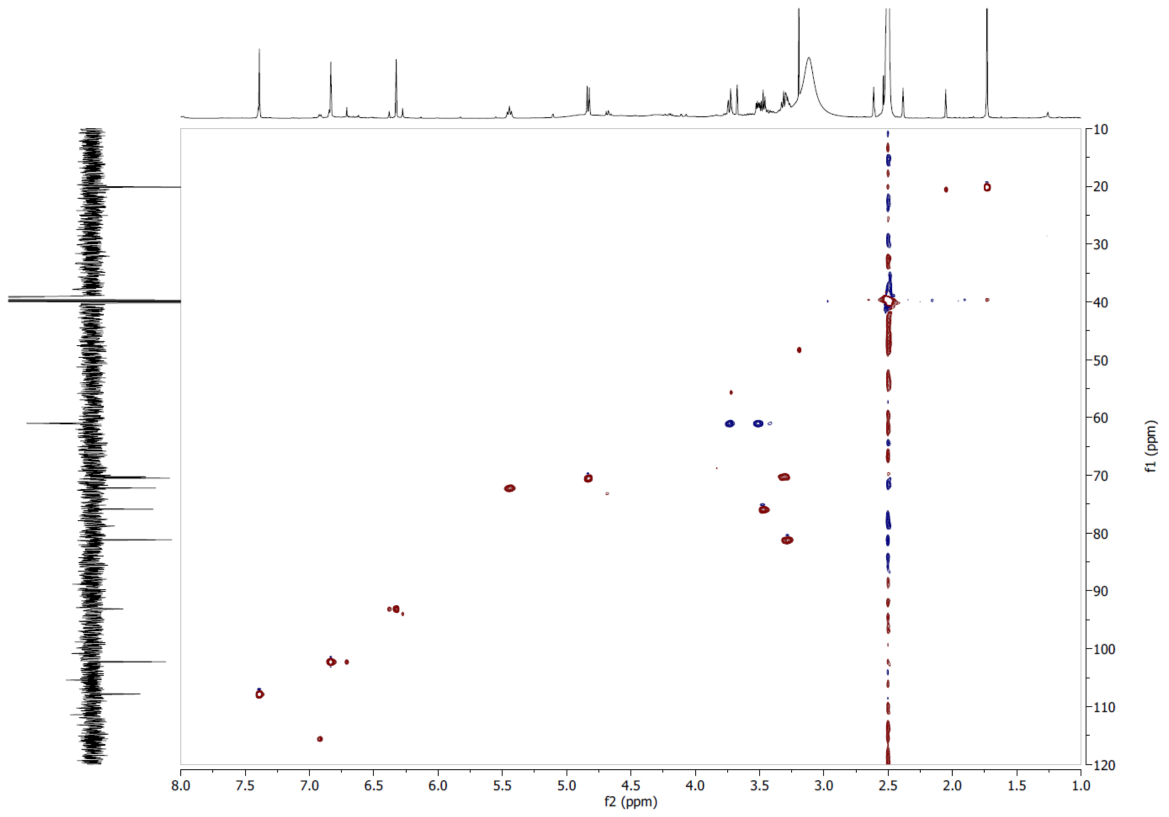

Supplementary Figure 2.31. Edited-HSQC NMR spectrum of 2'-*O*-acetylmangiferin (**4**) in DMSO-d_6 and T=343K

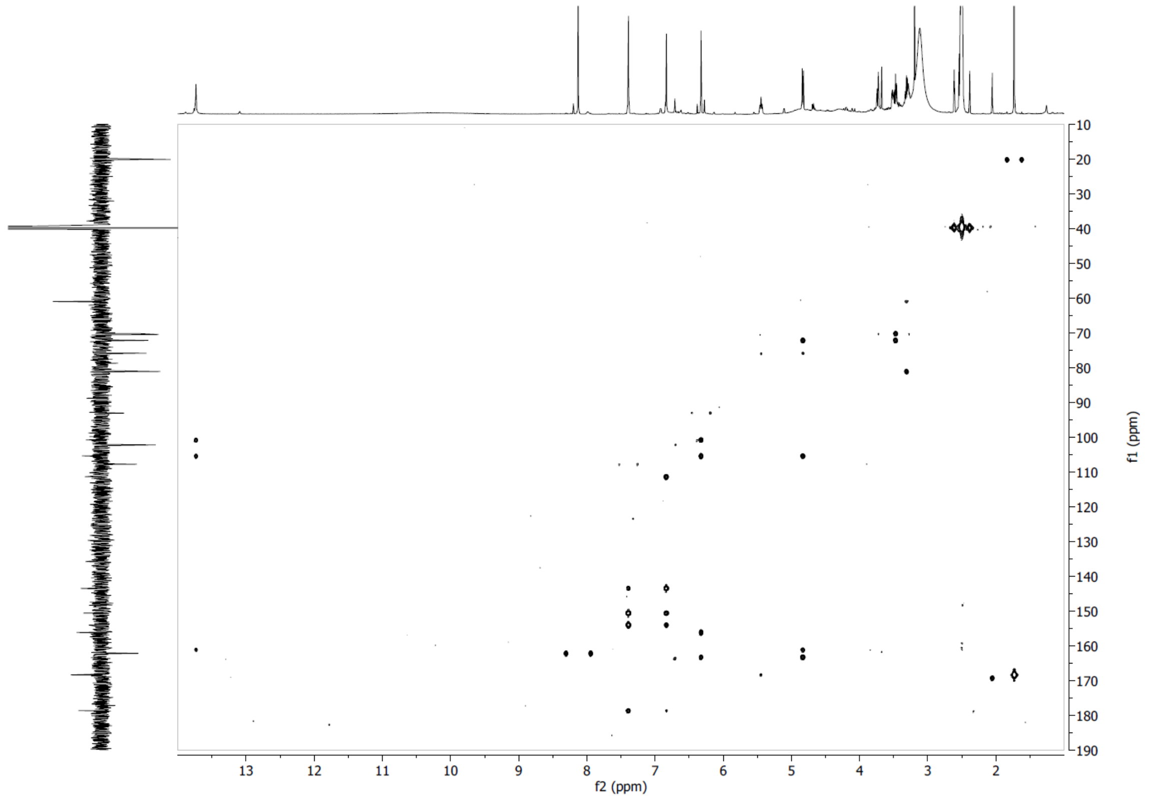

Supplementary Figure 2.32. HMBC NMR spectrum of 2'-*O*-acetylmangiferin (**4**) in DMSO-d_6 and T=343K

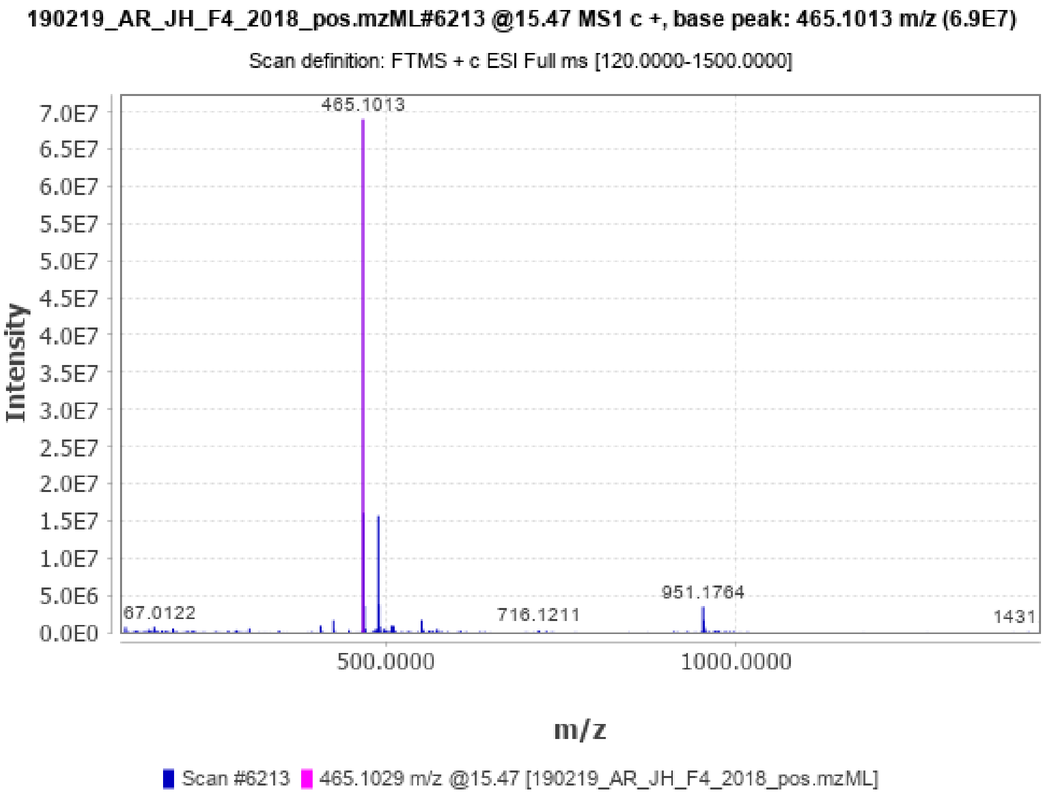

Supplementary Figure 2.33. HRMS spectrum of 2'-*O*-acetylmangiferin (**4**) ([M+H]$^+$) in Fraction 4 obtained by UHPLC-HRMS in positive ionization.

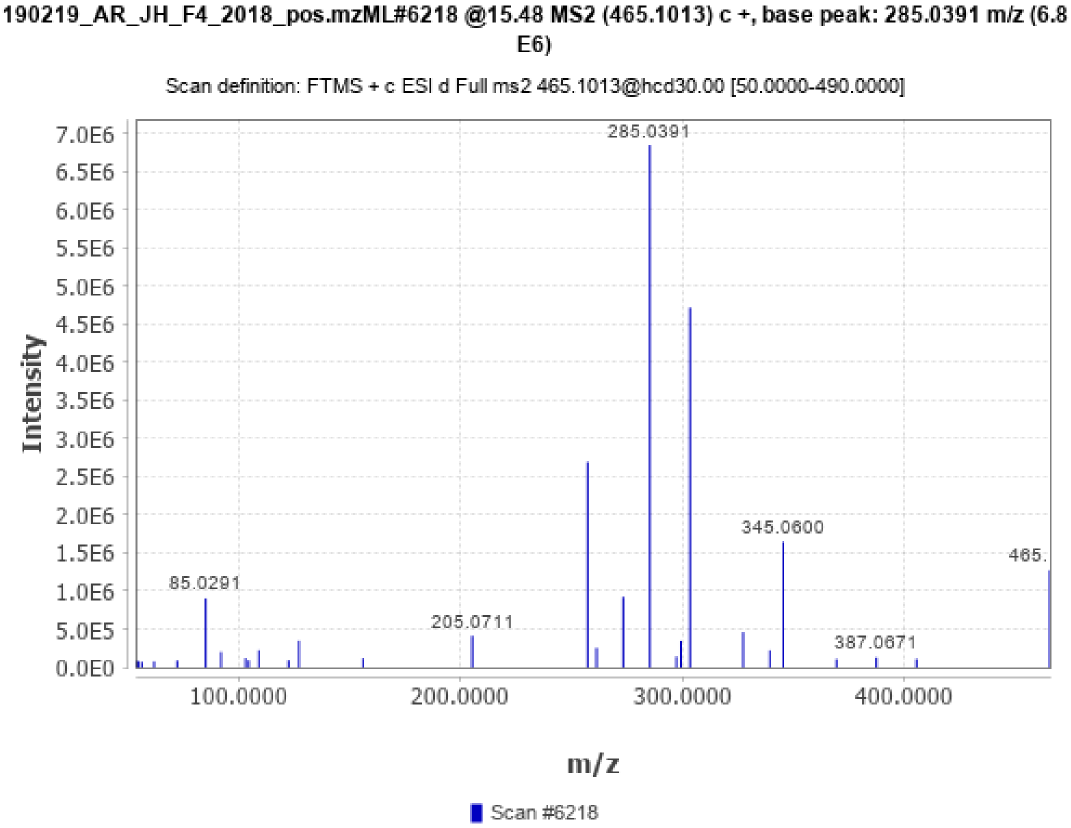

Supplementary Figure 2.34. Fragmentation spectrum of 2'-*O*-acetylmangiferin (**4**) ([M+H]$^+$) in Fraction 4 obtained by UHPLC-HRMS in positive ionization.

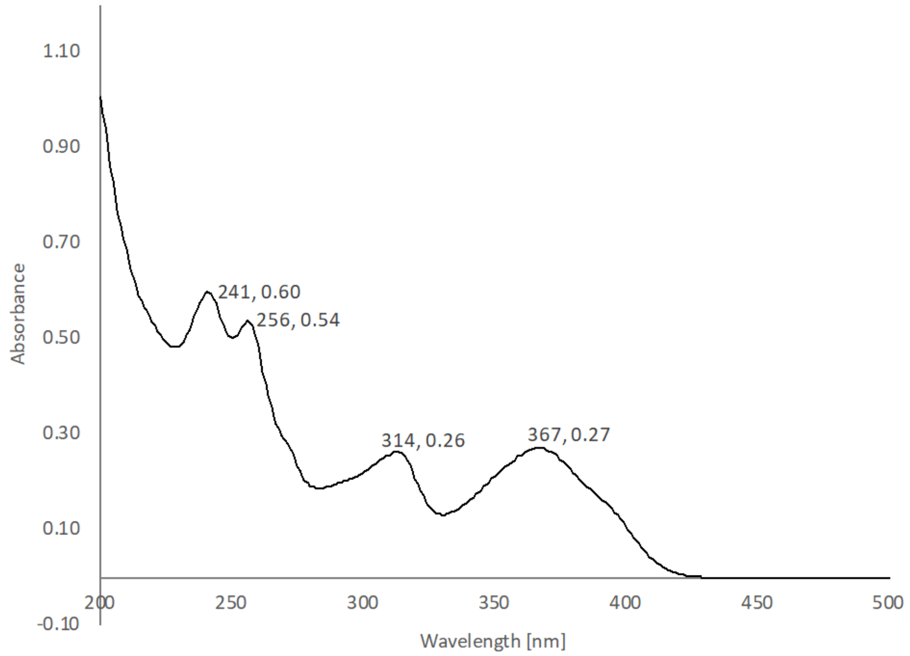

Supplementary Figure 2.35. UV spectrum of 2’-O-acetylmangiferin (**4**) in methanol.

5. 2-*C*-β-glucofuranosylmangiferin (5)

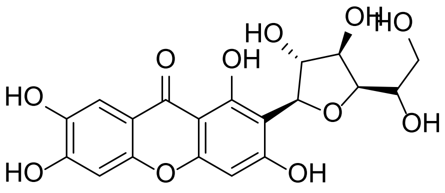

Chemical Formula: $C_{19}H_{18}O_{11}$
Exact Mass: 422.08

Supplementary Figure 2.36. Structure of 2-*C*-β-glucofuranosylmangiferin (5)

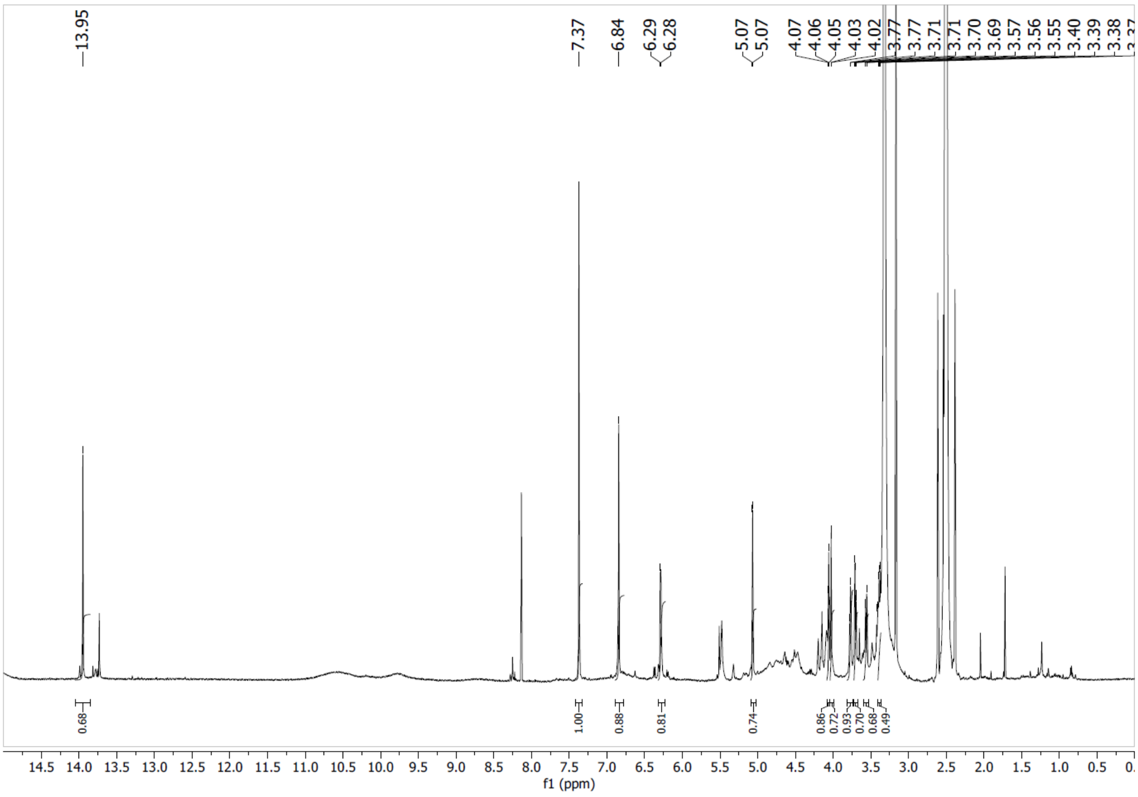

Supplementary Figure 2.37. ^{1}H NMR spectrum of 2-*C*-β-glucofuranosylmangiferin (**5**) in DMSO-d_6 at 600 MHz

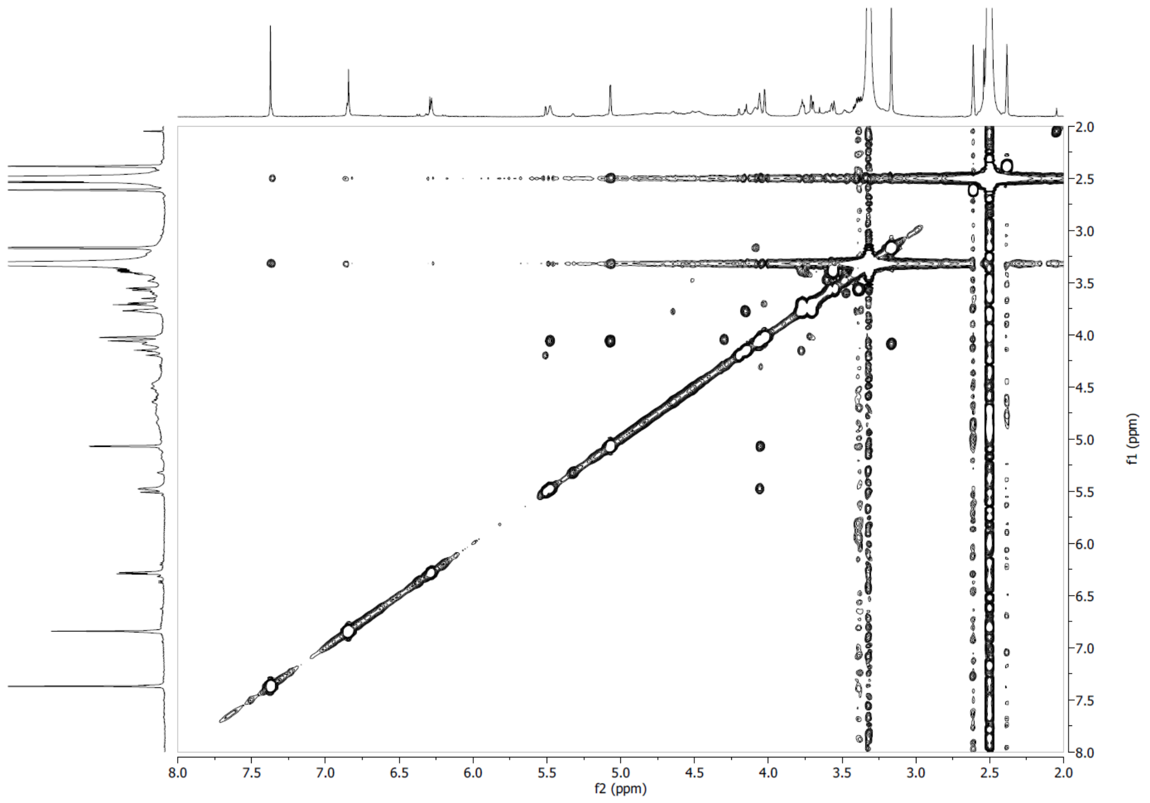

Supplementary Figure 2.38. COSY NMR spectrum of 2-*C*-β-glucofuranosylmangiferin (**5**) in DMSO-*d*6

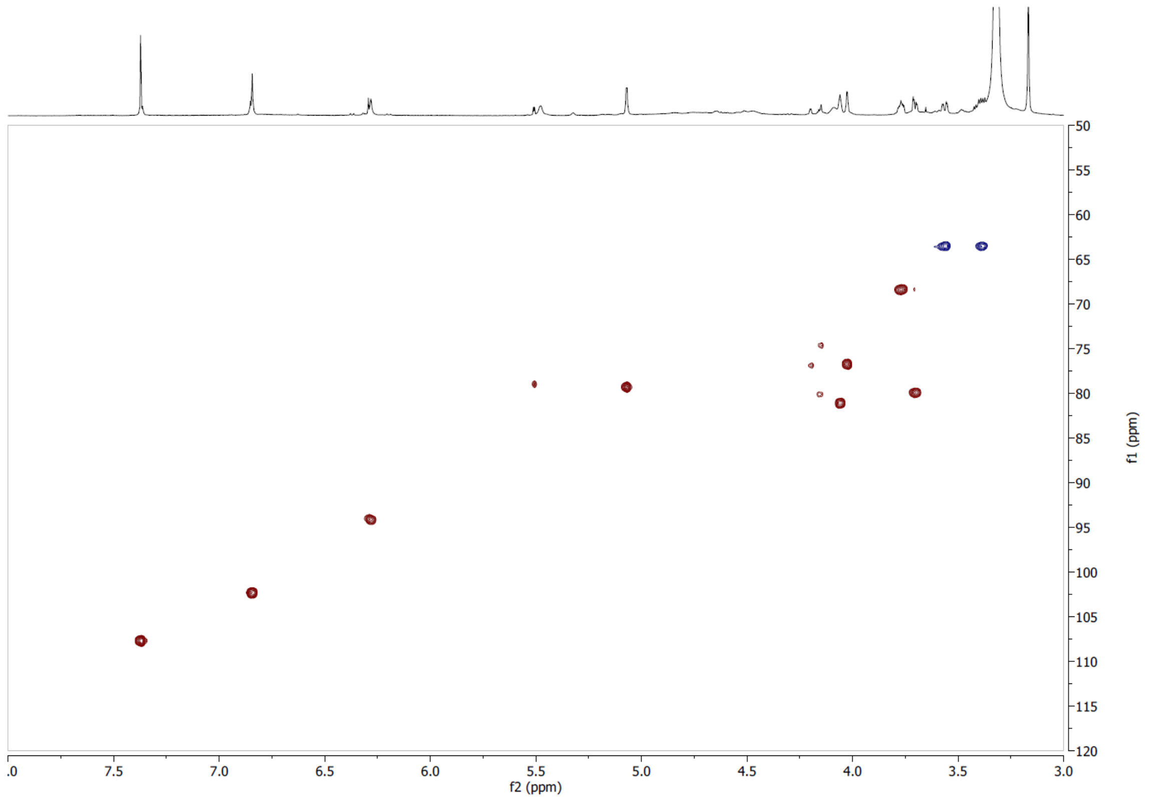

Supplementary Figure 2.39. Edited-HSQC NMR spectrum of 2-*C*-β-glucofuranosylmangiferin (**5**) in DMSO-*d*6

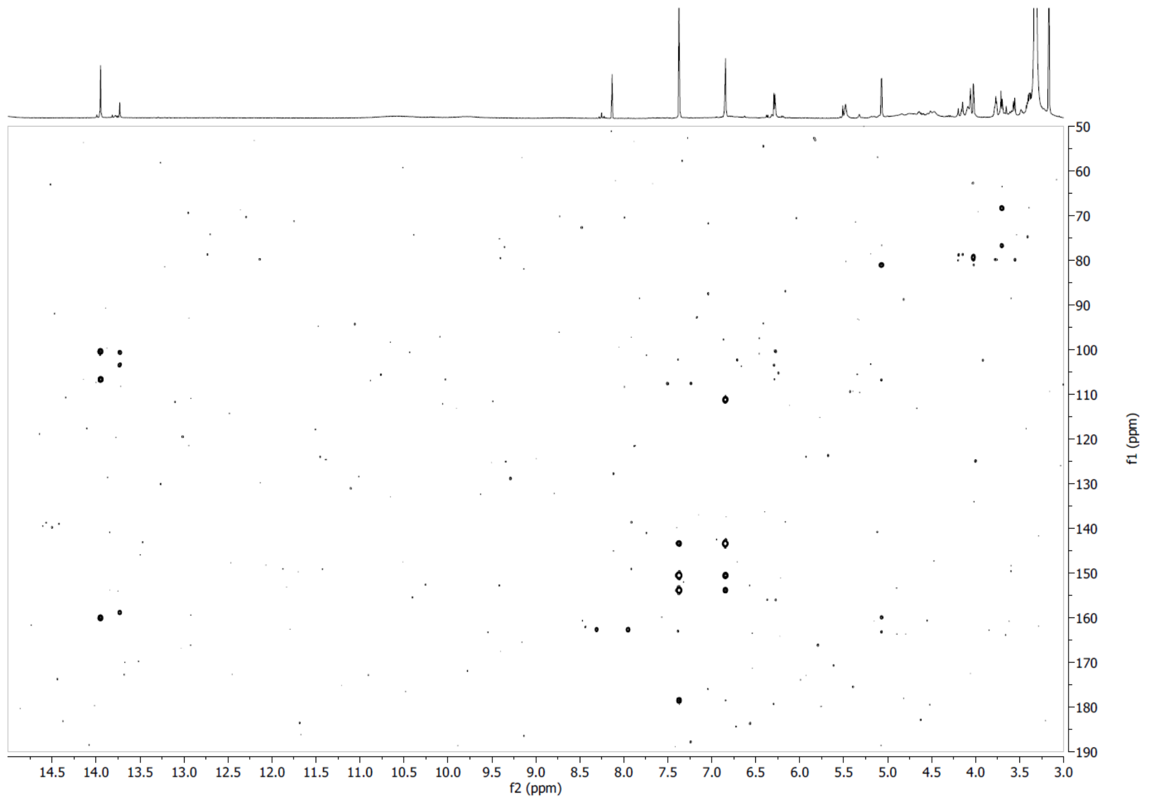

Supplementary Figure 2.40. HMBC NMR spectrum of 2-*C*-β-glucofuranosylmangiferin (**5**) in DMSO-*d*6

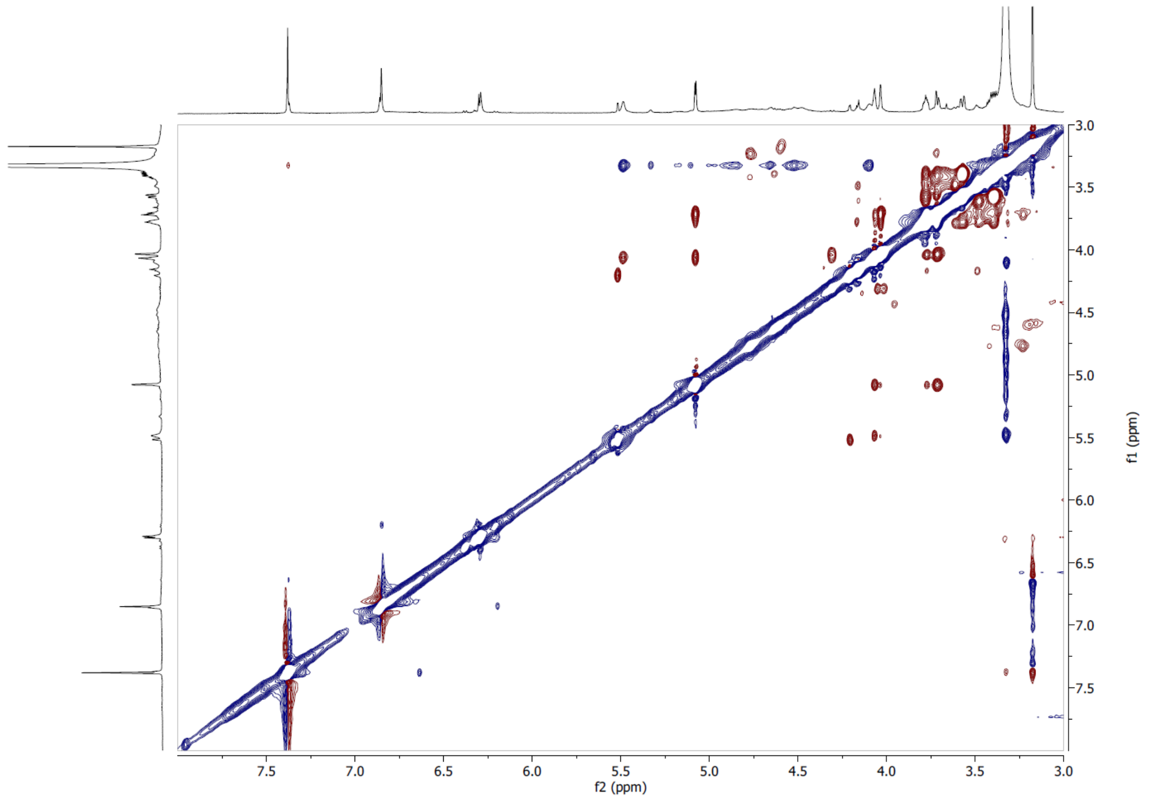

Supplementary Figure 2.41. ROESY NMR spectrum of 2-*C*-β-glucofuranosylmangiferin (**5**) in DMSO-*d*6

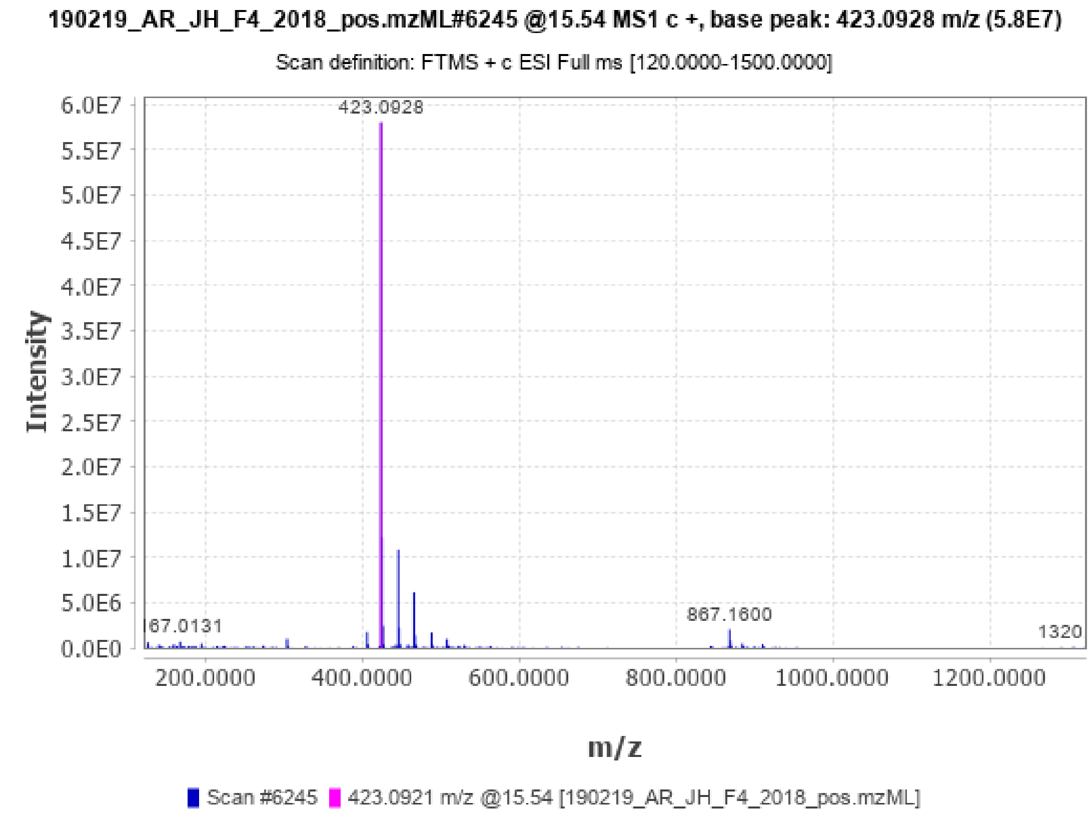

Supplementary Figure 2.42. HRMS spectrum of 2-*C*-β-glucofuranosylmangiferin (**5**) ([M+H]⁺) in Fraction 4 obtained by UHPLC-HRMS in positive ionization.

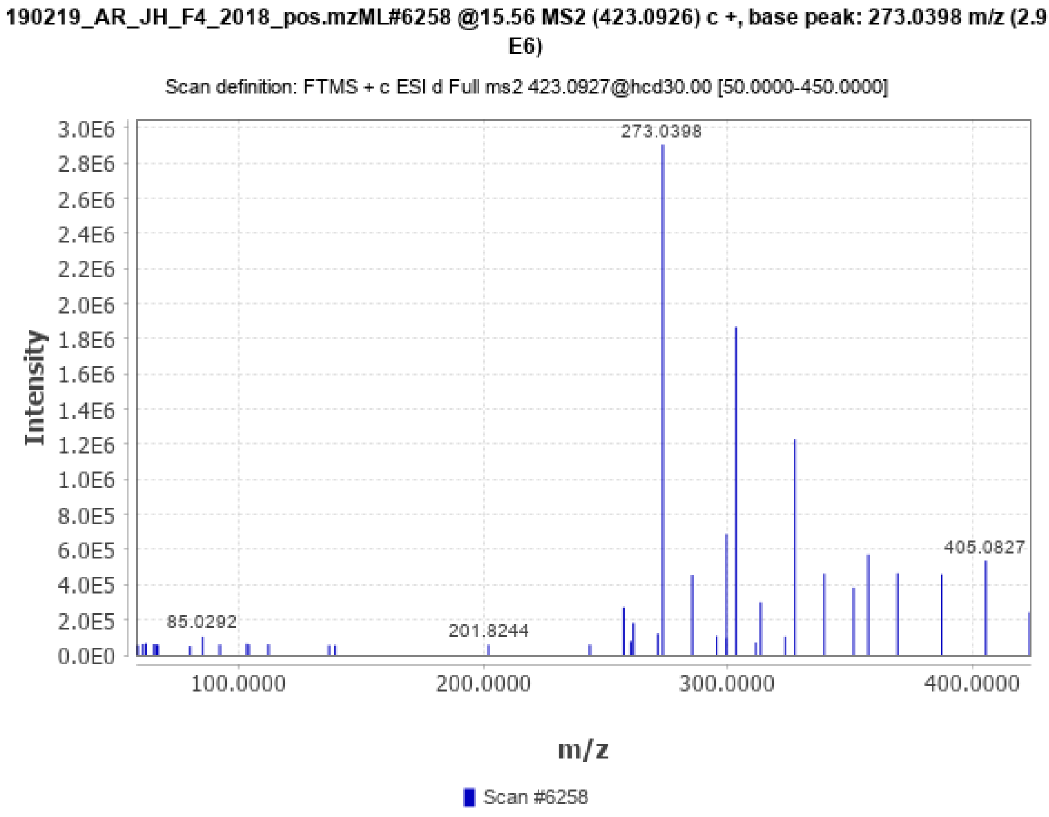

Supplementary Figure 2.43. Fragmentation spectrum of 2-*C*-β-glucofuranosylmangiferin (**5**) ([M+H]⁺) in Fraction 4 obtained by UHPLC-HRMS in positive ionization.

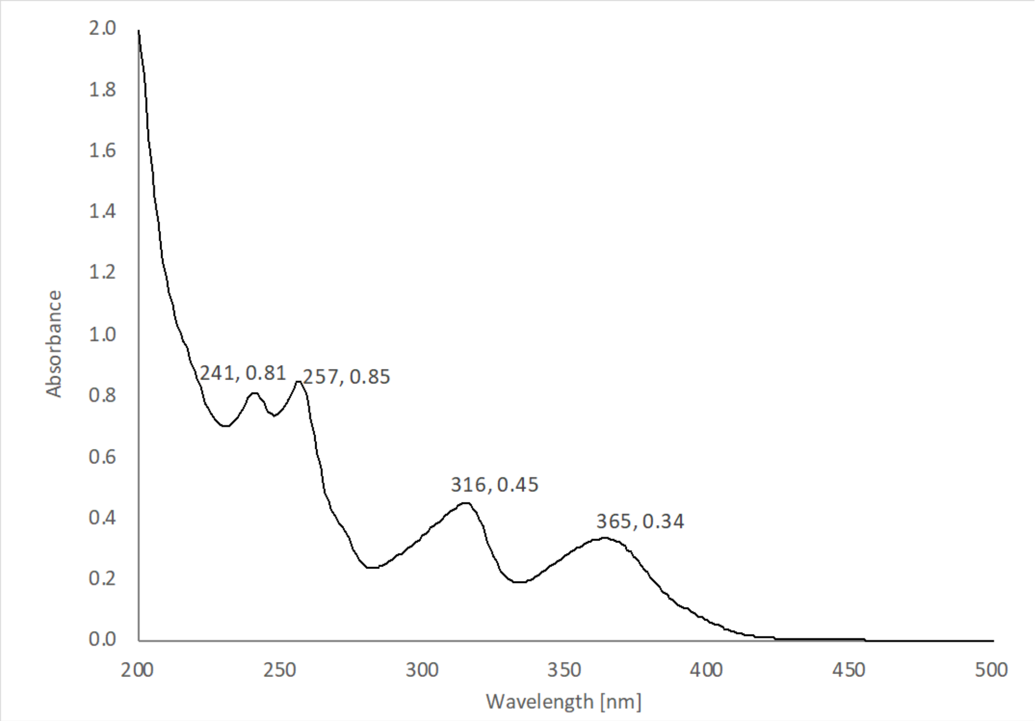

Supplementary Figure 2.44. UV spectrum of 2-*C*-β-glucofuranosylmangiferin (**5**) in methanol.

6. Isovitexin (6)

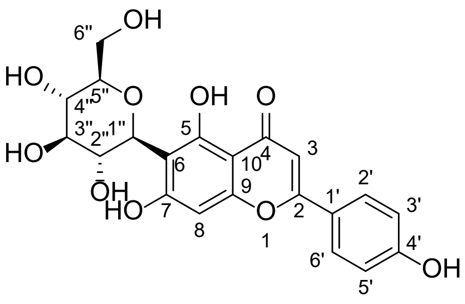

Chemical Formula: $C_{21}H_{20}O_{10}$
Exact Mass: 432.11

Supplementary Figure 2.45. Structure of isovitexin (**6**)

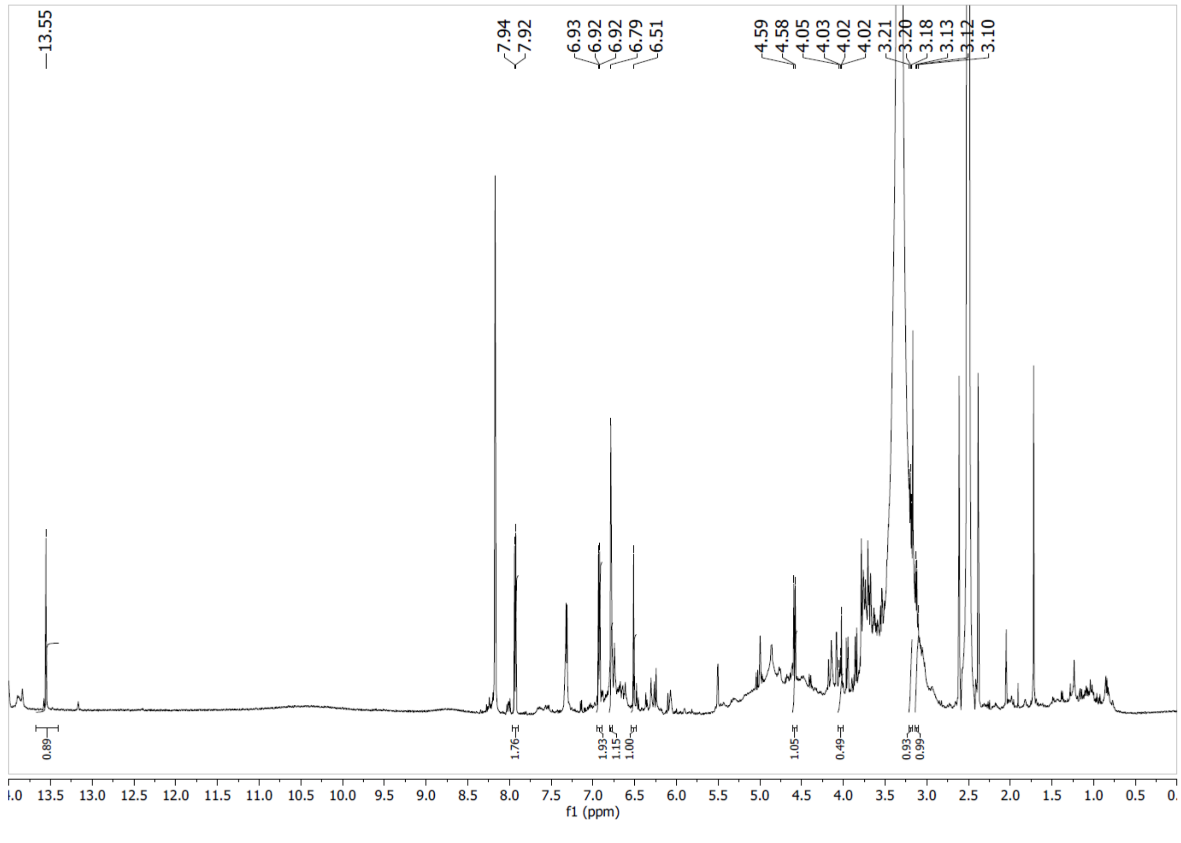

Supplementary Figure 2.46. ^{1}H NMR spectrum of isovitexin (**6**) in DMSO-d_6 at 600 MHz

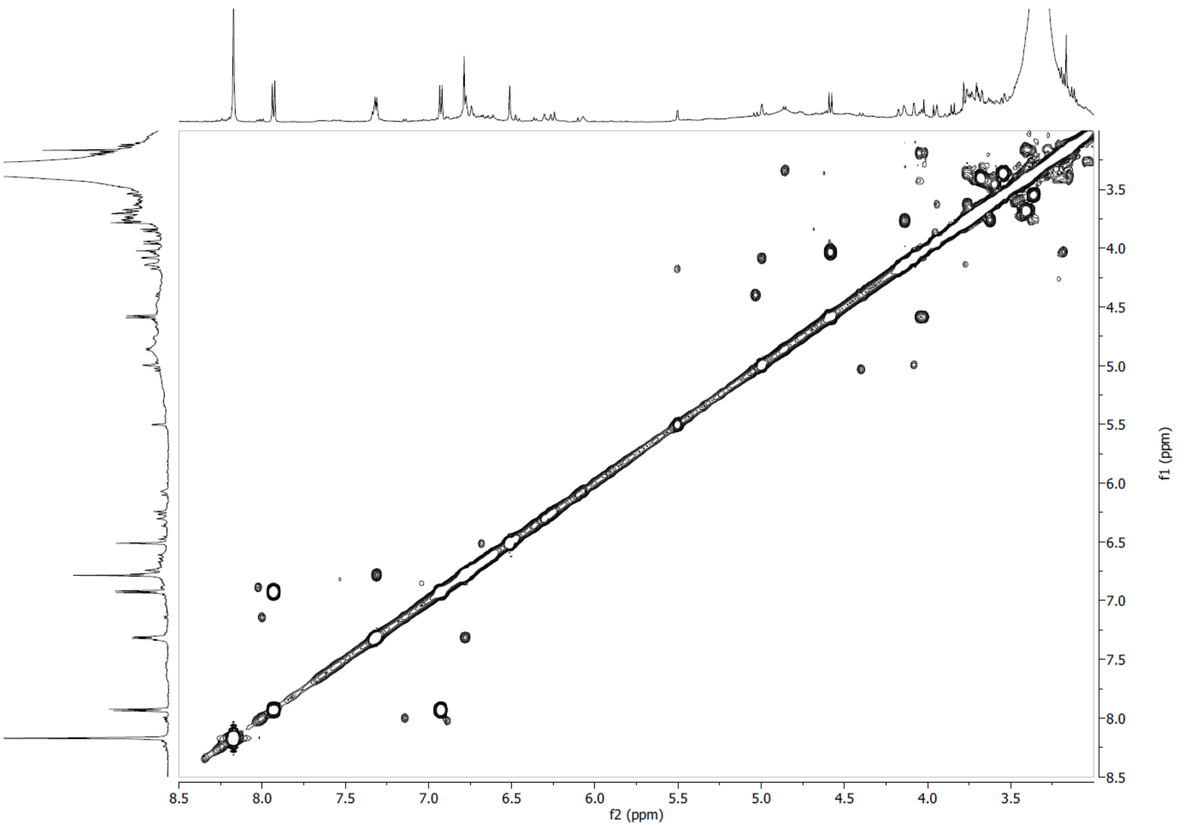

Supplementary Figure 2.47. COSY NMR spectrum of isovitexin (**6**) in DMSO-d_6

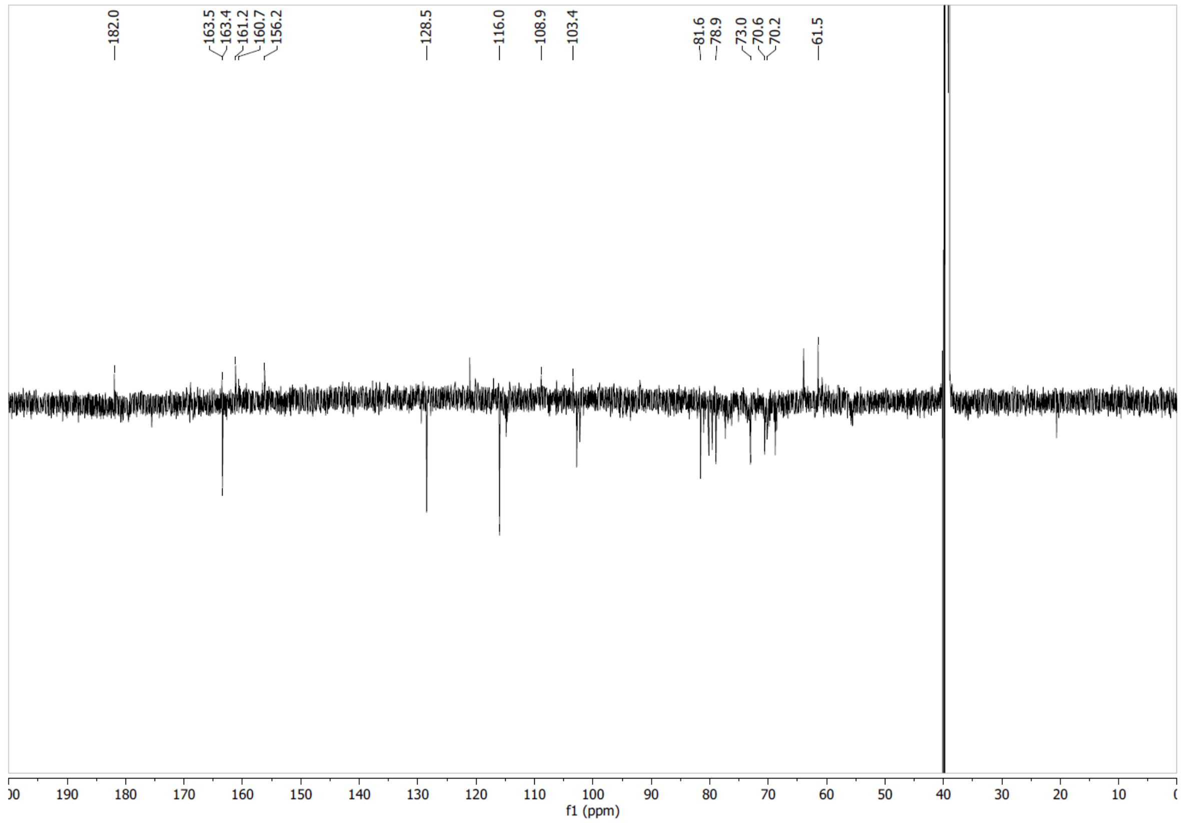

Supplementary Figure 2.48. ^{13}C-DEPTQ NMR spectrum of isovitexin (**6**) in DMSO-d_6 at 151 MHz

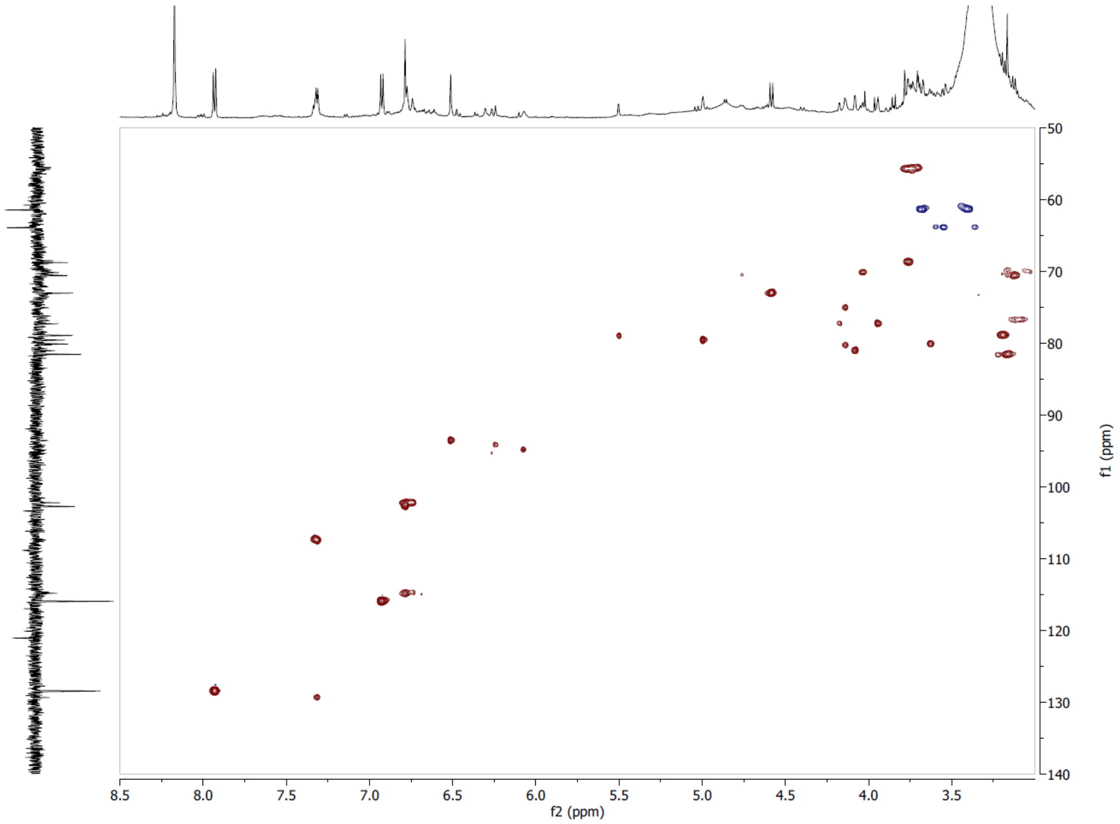

Supplementary Figure 2.49. Edited-HSQC NMR spectrum of isovitexin (**6**) in DMSO-d_6

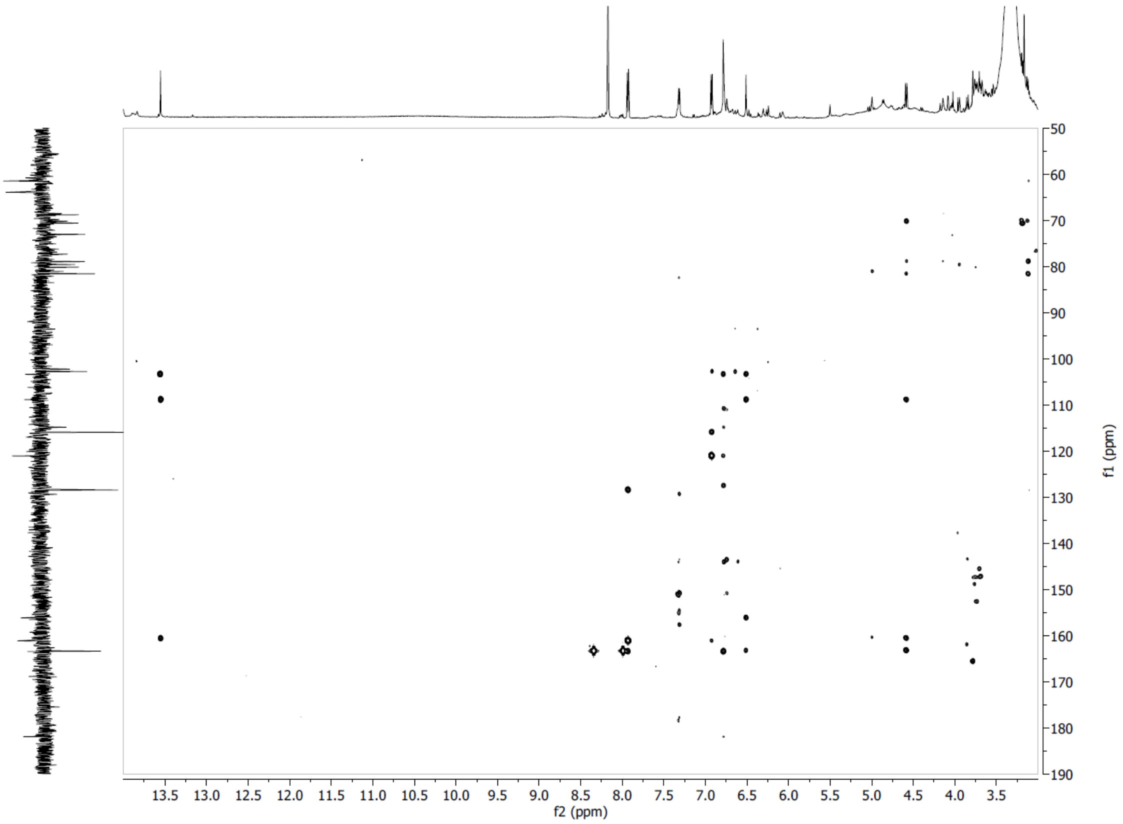

Supplementary Figure 2.50. HMBC NMR spectrum of isovitexin (**6**) in DMSO-d_6

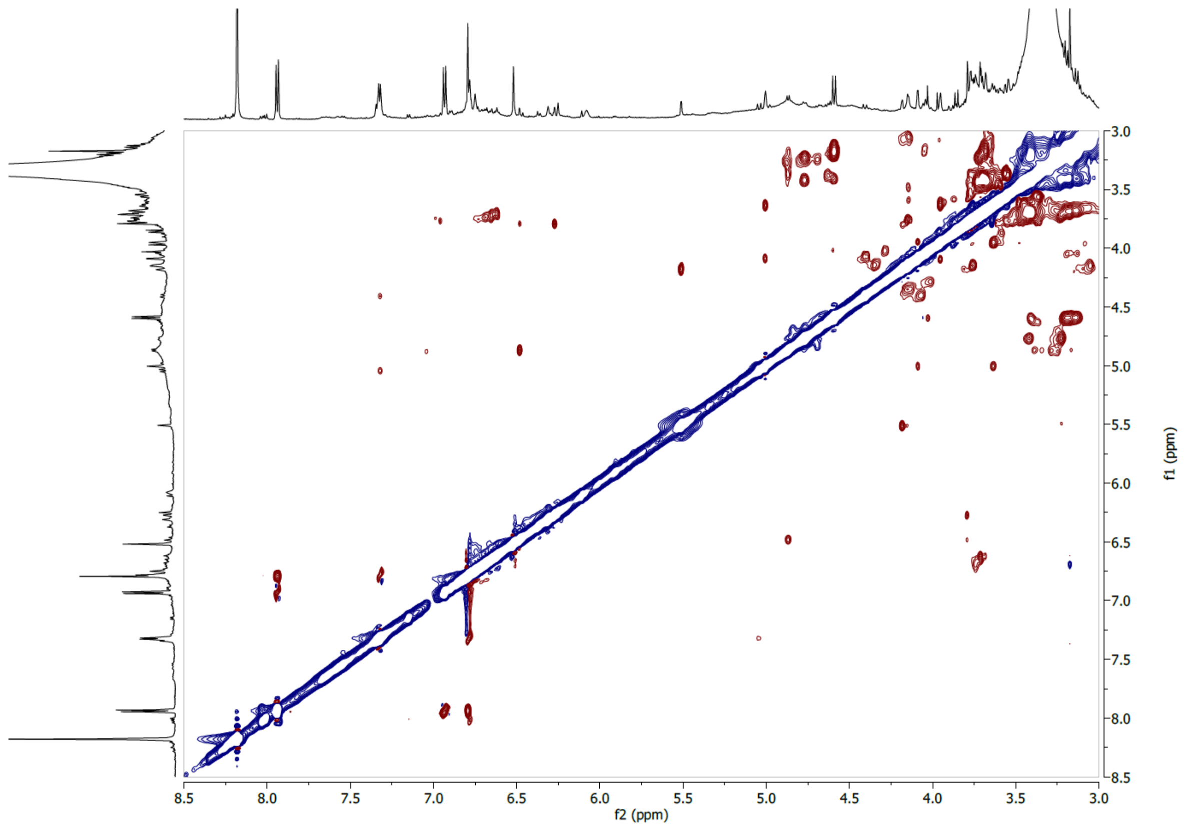

Supplementary Figure 2.51. ROESY NMR spectrum of isovitexin (**6**) in DMSO-d_6

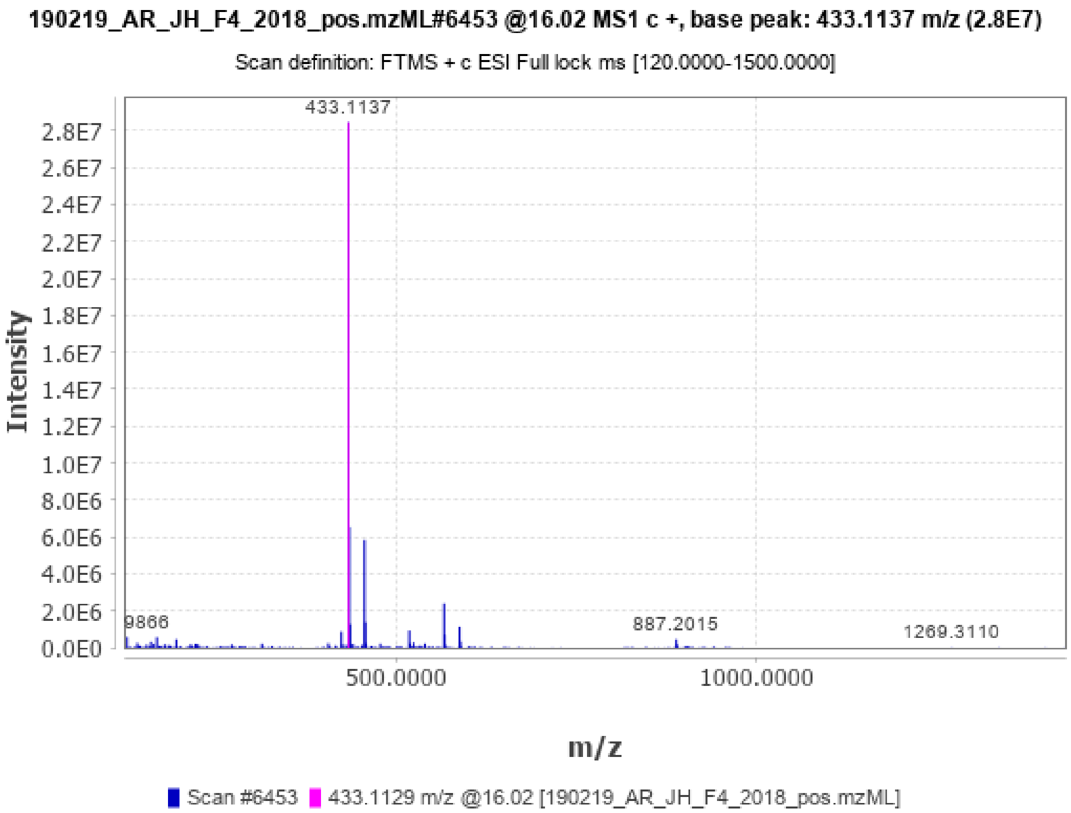

Supplementary Figure 2. 52. HRMS spectrum of isovitexin (**6**) ([M+H]+) in Fraction 4 obtained by UHPLC-HRMS in positive ionization.

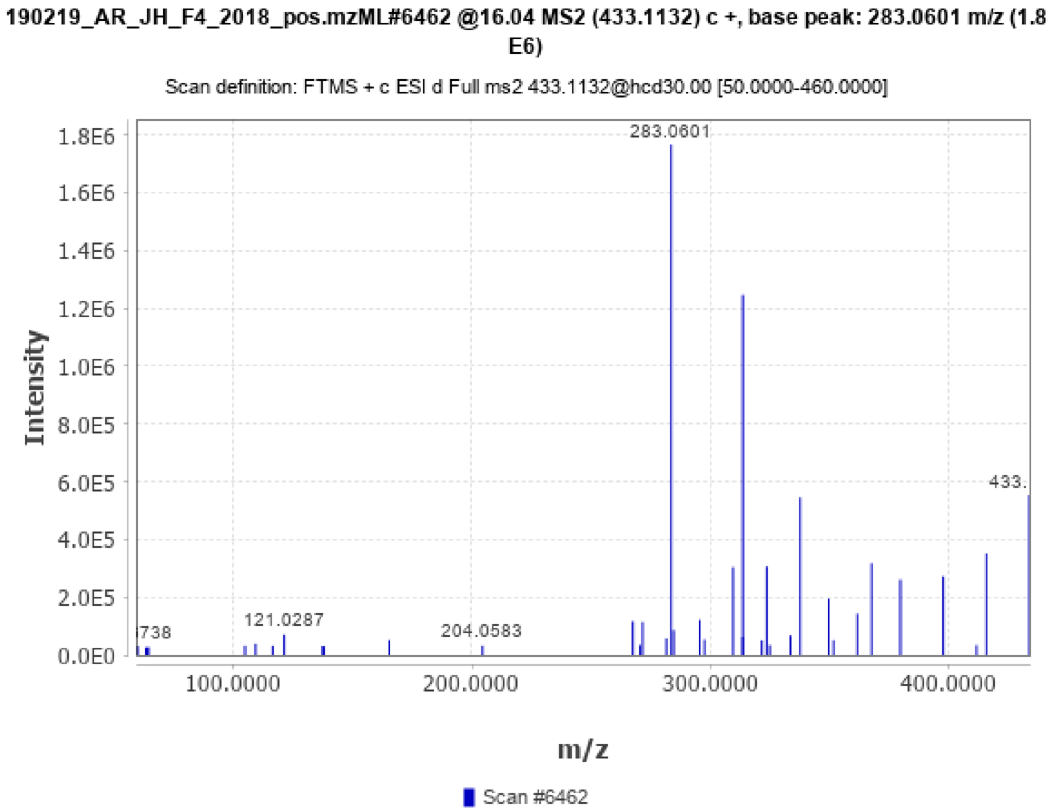

Supplementary Figure 2.53. Fragmentation spectrum of isovitexin (**6**) ([M+H]+) in Fraction 4 obtained by UHPLC-HRMS in positive ionization.

7. 2-*C*-α-glucofuranosylmangiferin (7)

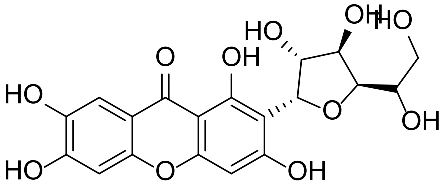

Chemical Formula: $C_{19}H_{18}O_{11}$

Exact Mass: 422.08

Supplementary Figure 2.54. Structure of 2-*C*-α-glucofuranosylmangiferin (**7**)

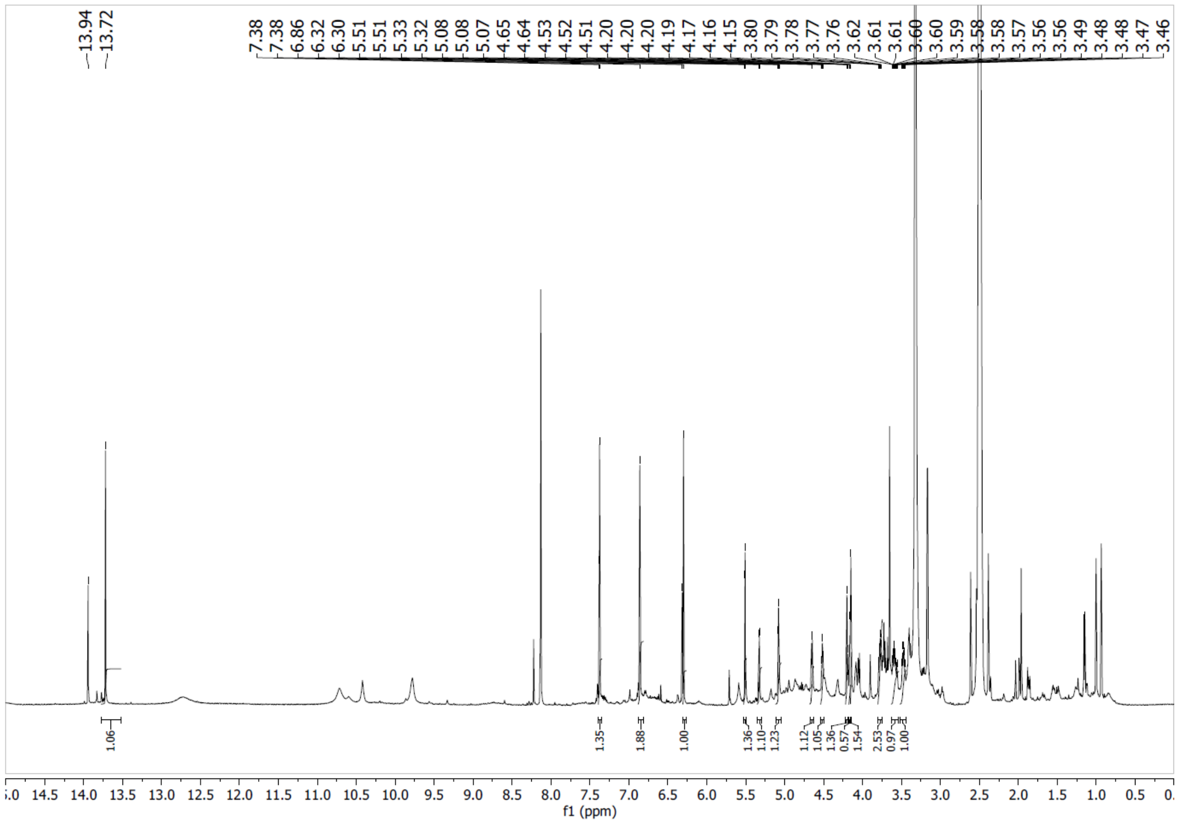

Supplementary Figure 2.55. ^{1}H NMR spectrum of 2-*C*-α-glucofuranosylmangiferin (**7**) in DMSO-d_6 at 600 MHz

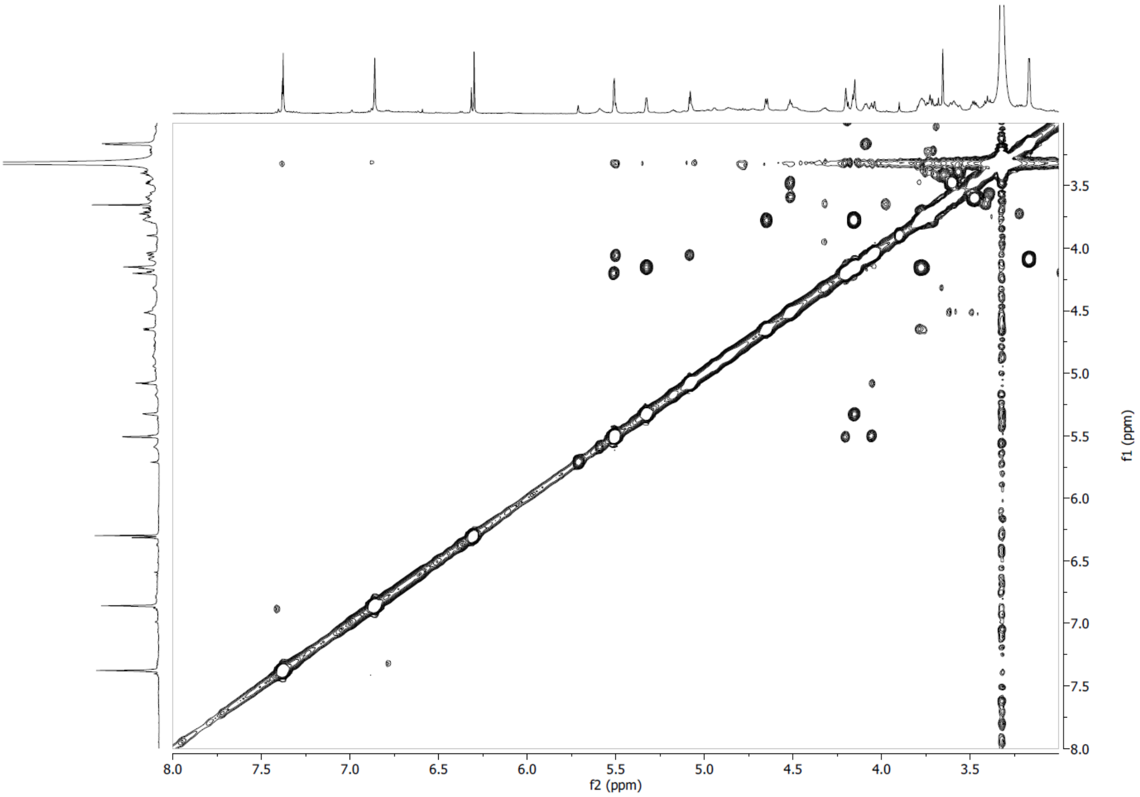

Supplementary Figure 2.56. COSY NMR spectrum of 2-*C*-α-glucofuranosylmangiferin (**7**) in DMSO-d_6

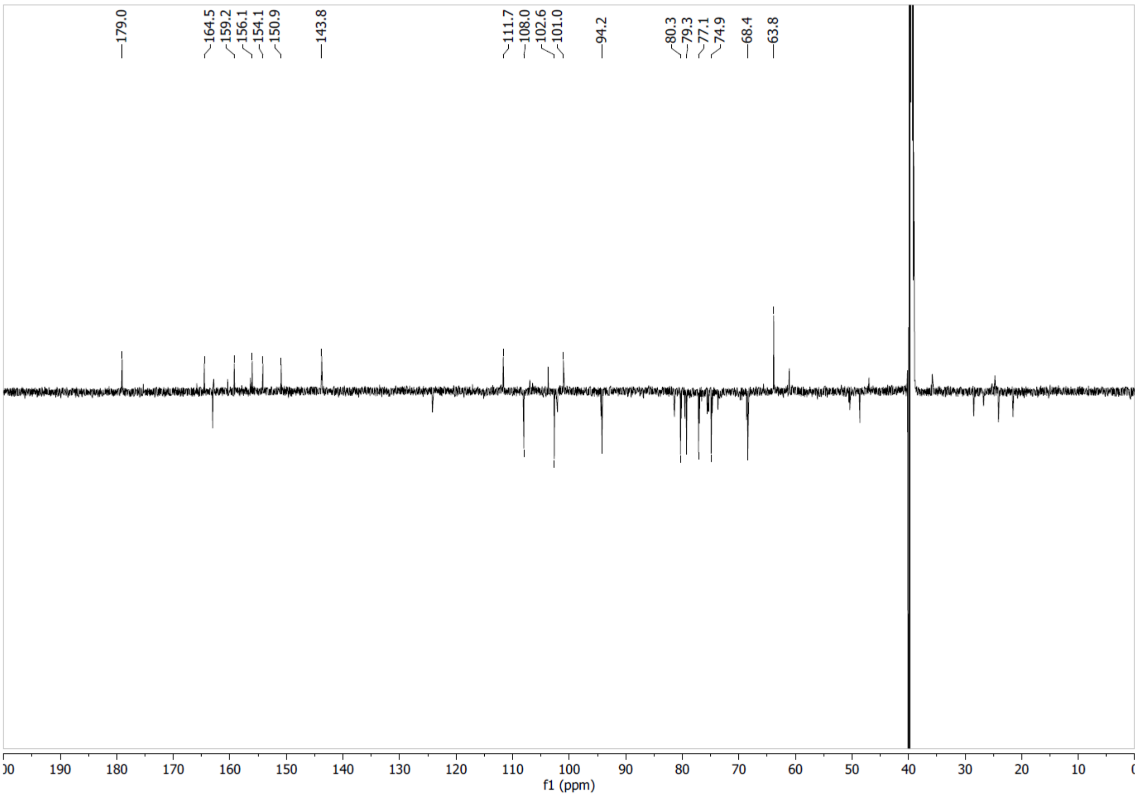

Supplementary Figure 2. 57. ^{13}C-DEPTQ NMR spectrum of 2-*C*-α-glucofuranosylmangiferin (**7**) in DMSO-d_6 at 151 MHz

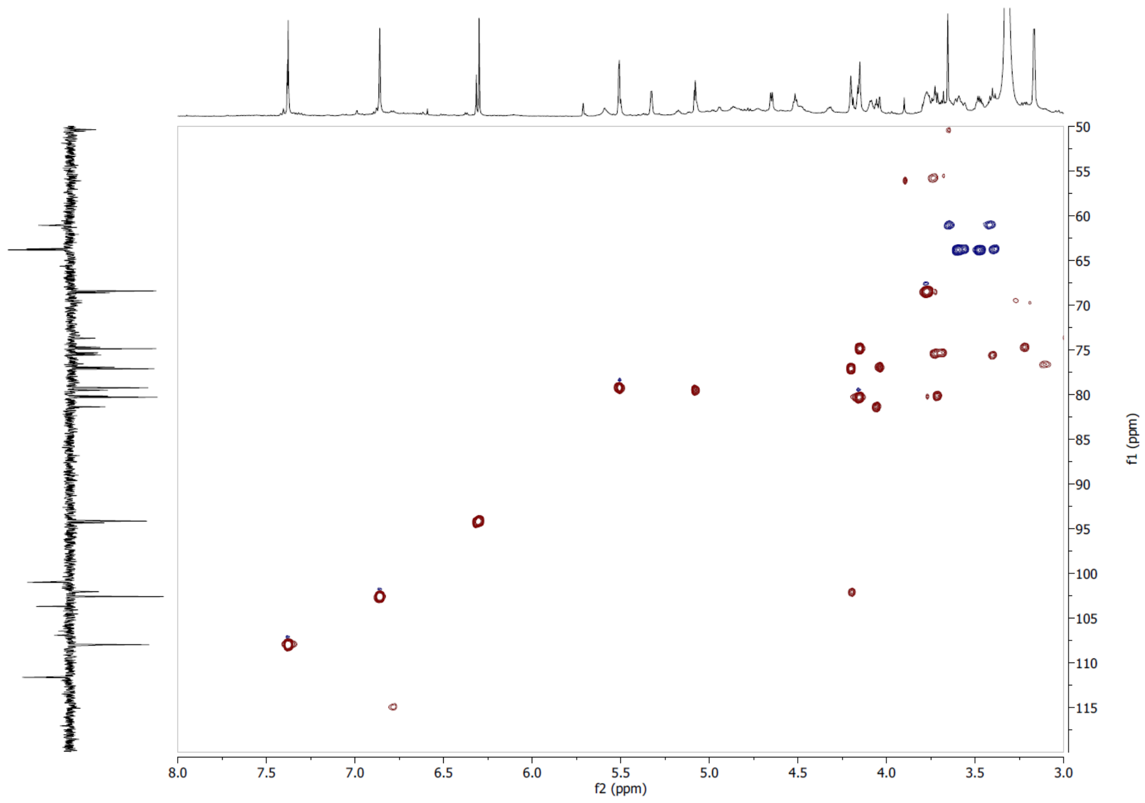

Supplementary Figure 2.58. Edited-HSQC NMR spectrum of 2-*C*-α-glucofuranosylmangiferin (**7**) in DMSO-d_6

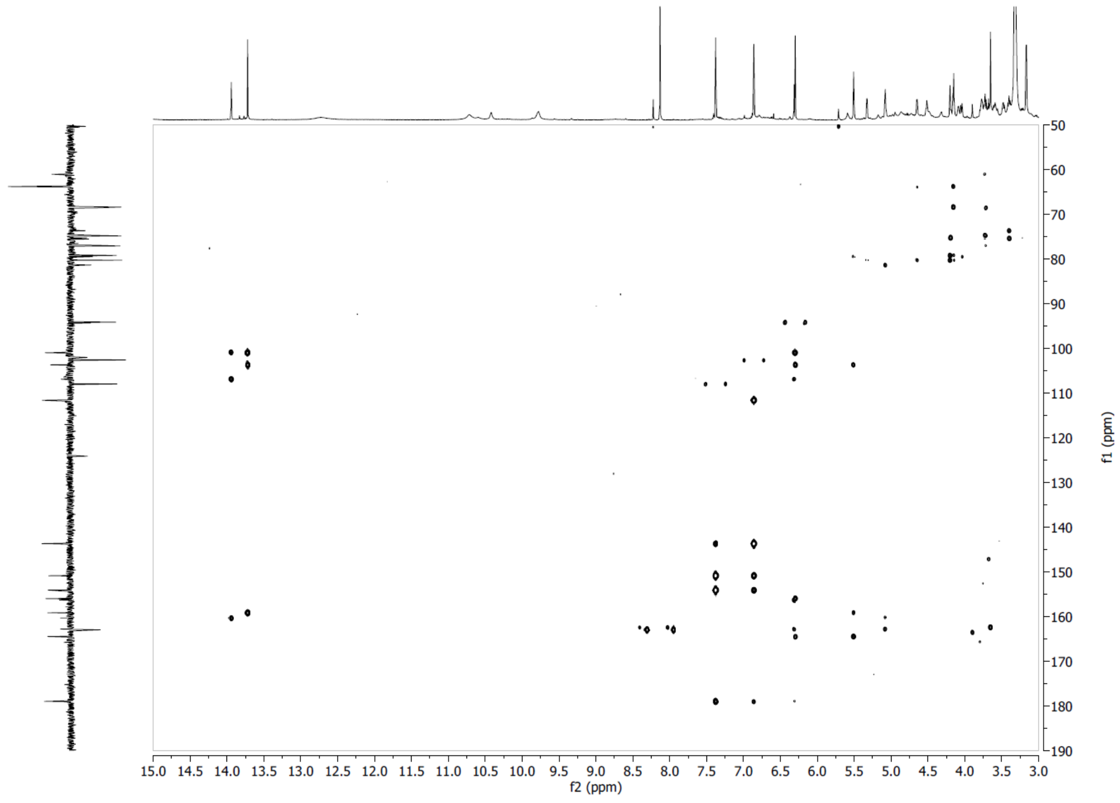

Supplementary Figure 2.59. HMBC NMR spectrum of 2-*C*-α-glucofuranosylmangiferin (**7**) in DMSO-d_6

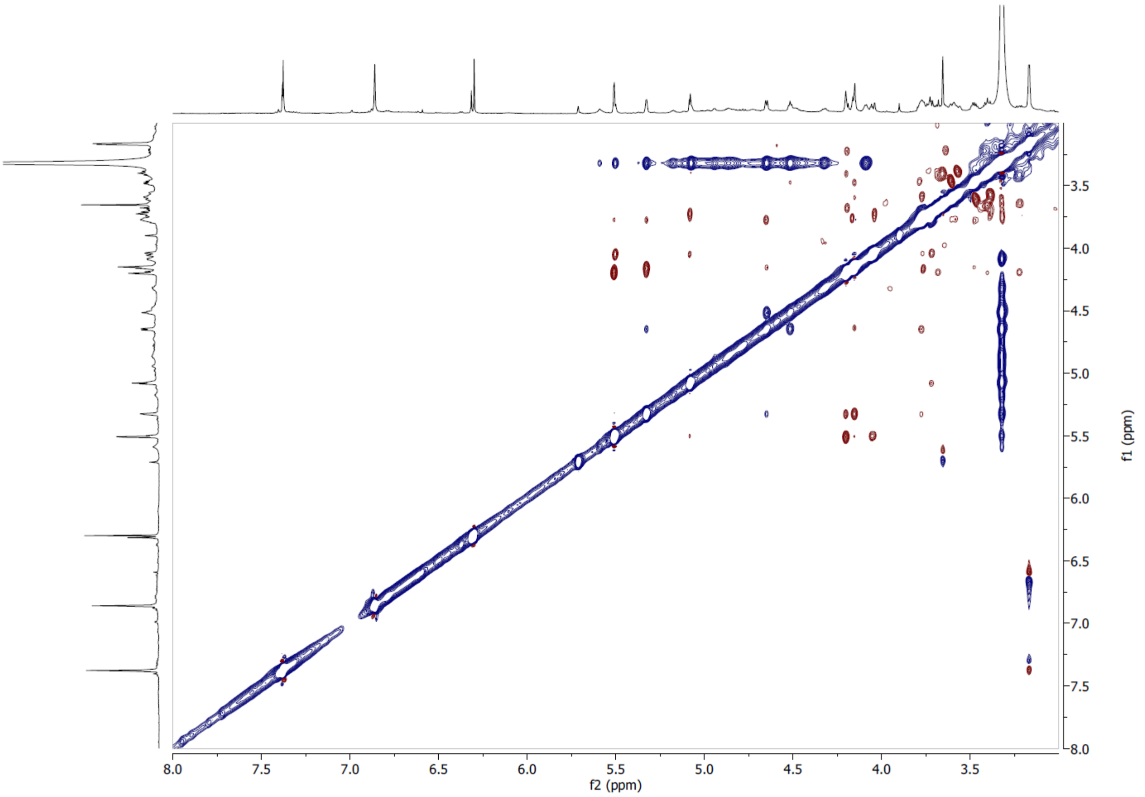

Supplementary Figure 2.60. ROESY NMR spectrum of 2-*C*-α-glucofuranosylmangiferin (**7**) in DMSO-d_6

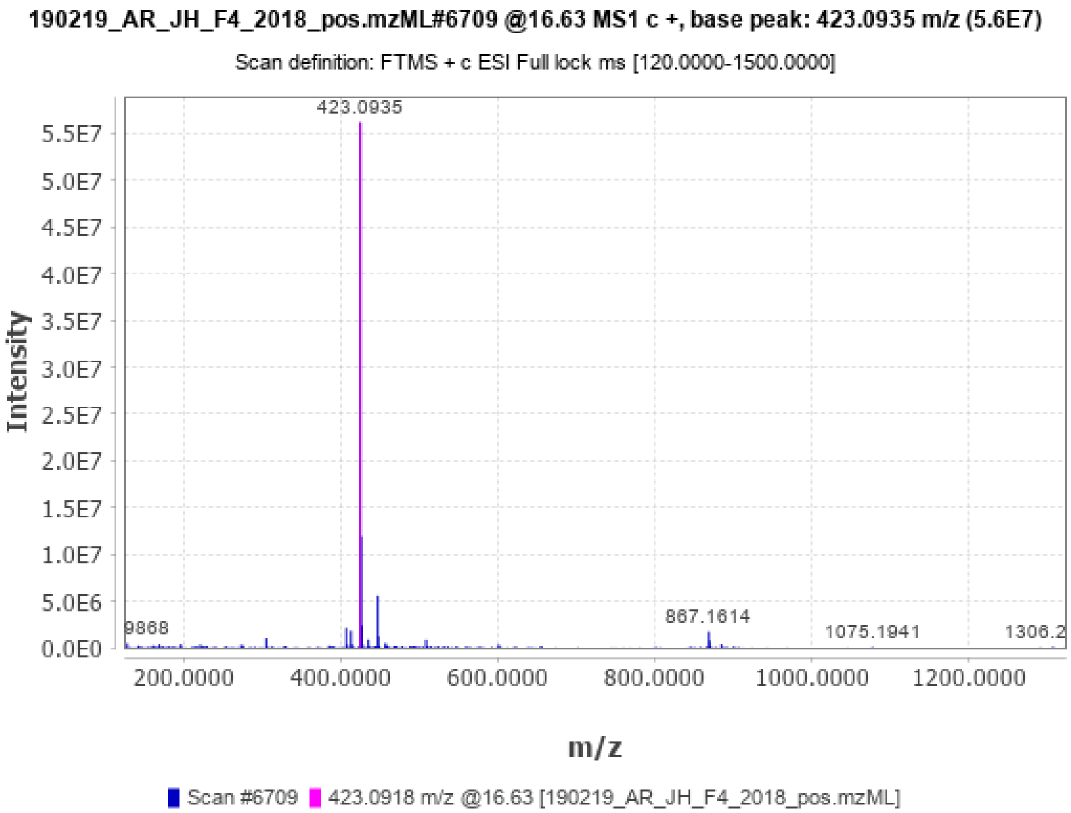

Supplementary Figure 2. 61. HRMS spectrum of 2-*C*-α glucofuranosylmangiferin (**7**) ([M+H]$^+$) in Fraction 4 obtained by UHPLC-HRMS in positive ionization.

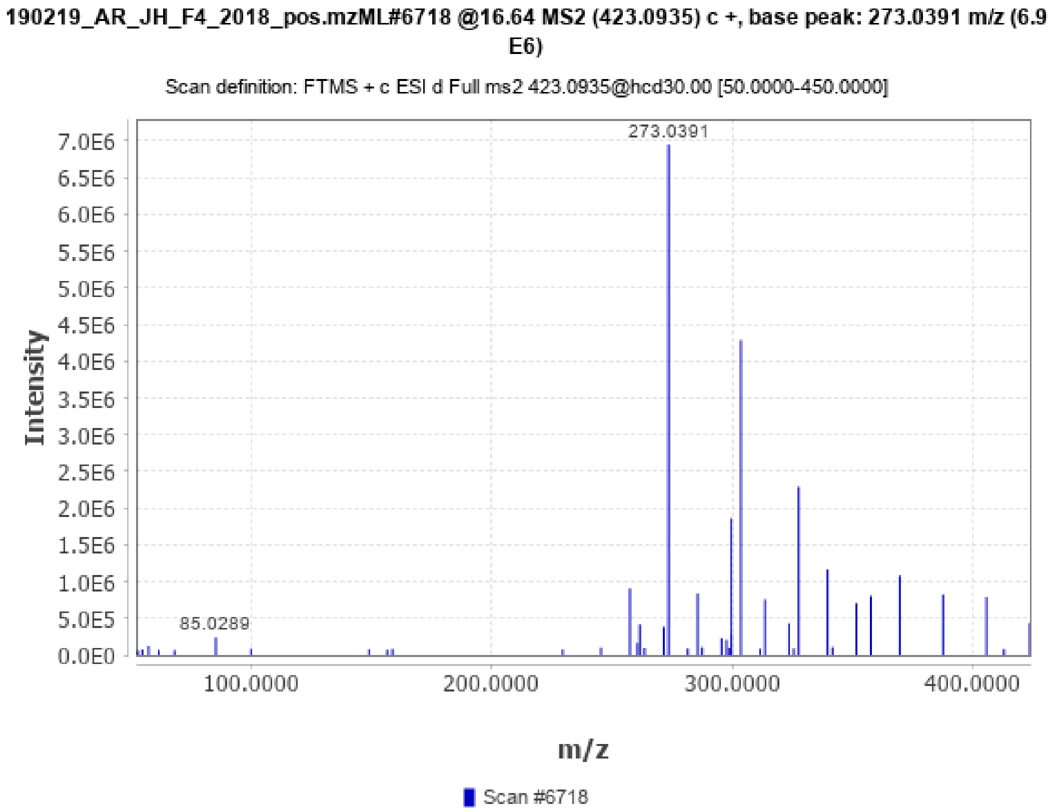

Supplementary Figure 2.62. Fragmentation spectrum of 2-*C*-α-glucofuranosylmangiferin (**7**) ([M+H]$^+$) in Fraction 4 obtained by UHPLC-HRMS in positive ionization.

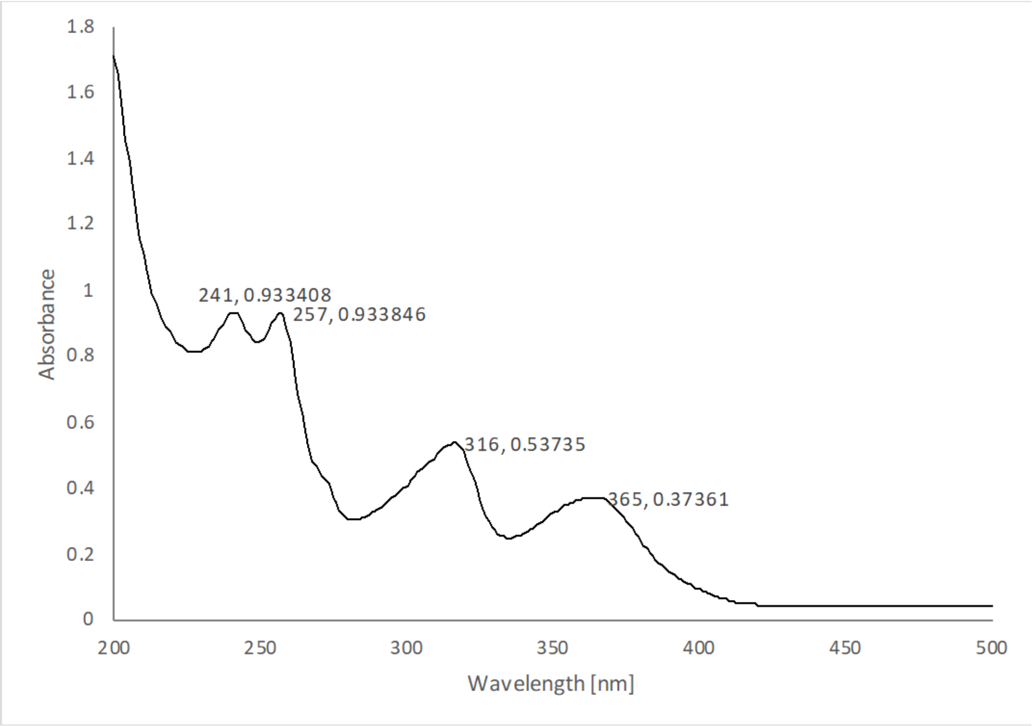

Supplementary Figure 2.63. UV spectrum of 2-*C*-α-glucofuranosylmangiferin (**7**) in methanol.

8. 6'-*O*-acetylmangiferin (8)

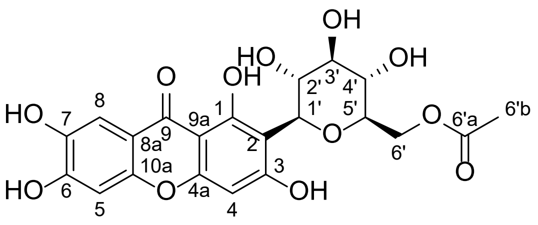

Chemical Formula: $C_{21}H_{20}O_{12}$
Exact Mass: 464.10

Supplementary Figure 2.64. Structure of 6'-O-acetylmangiferin (**8**)

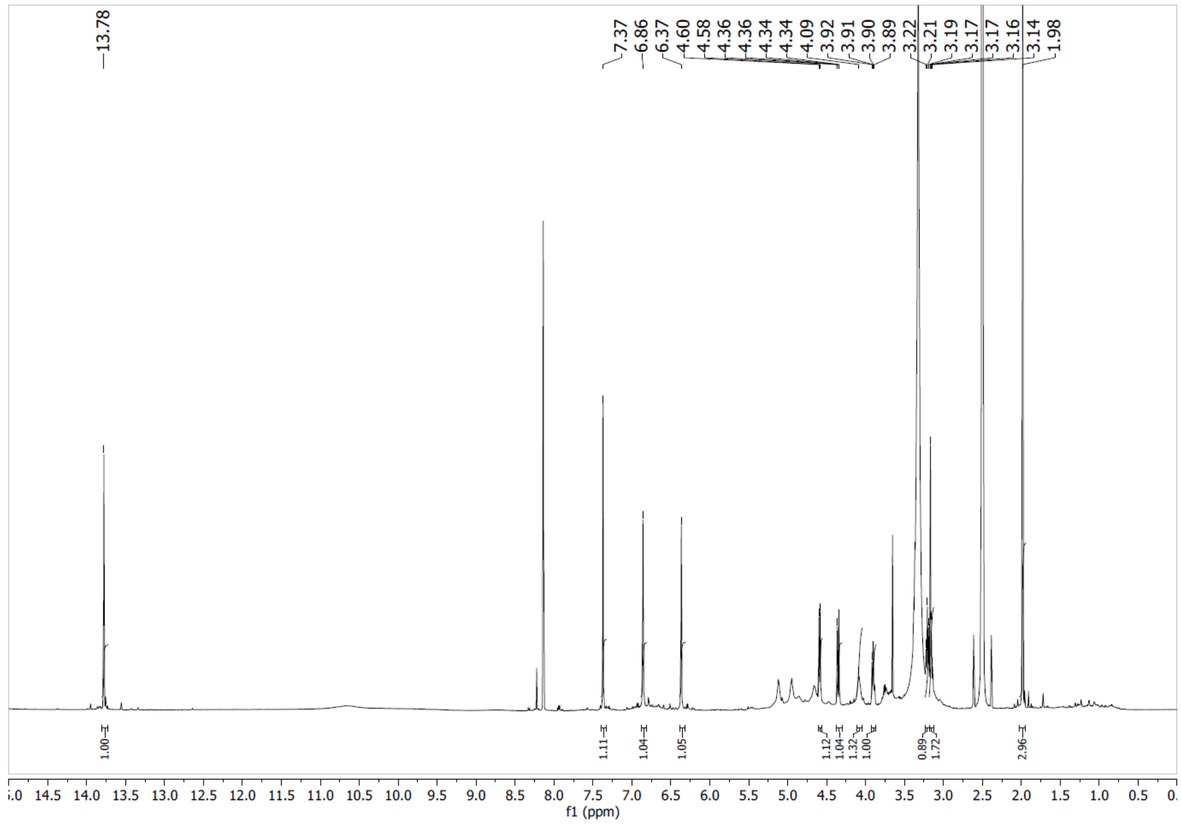

Supplementary Figure 2. 65. ^{1}H NMR spectrum of 6'-*O*-acetylmangiferin (**8**) in DMSO-d_6 at 600 MHz

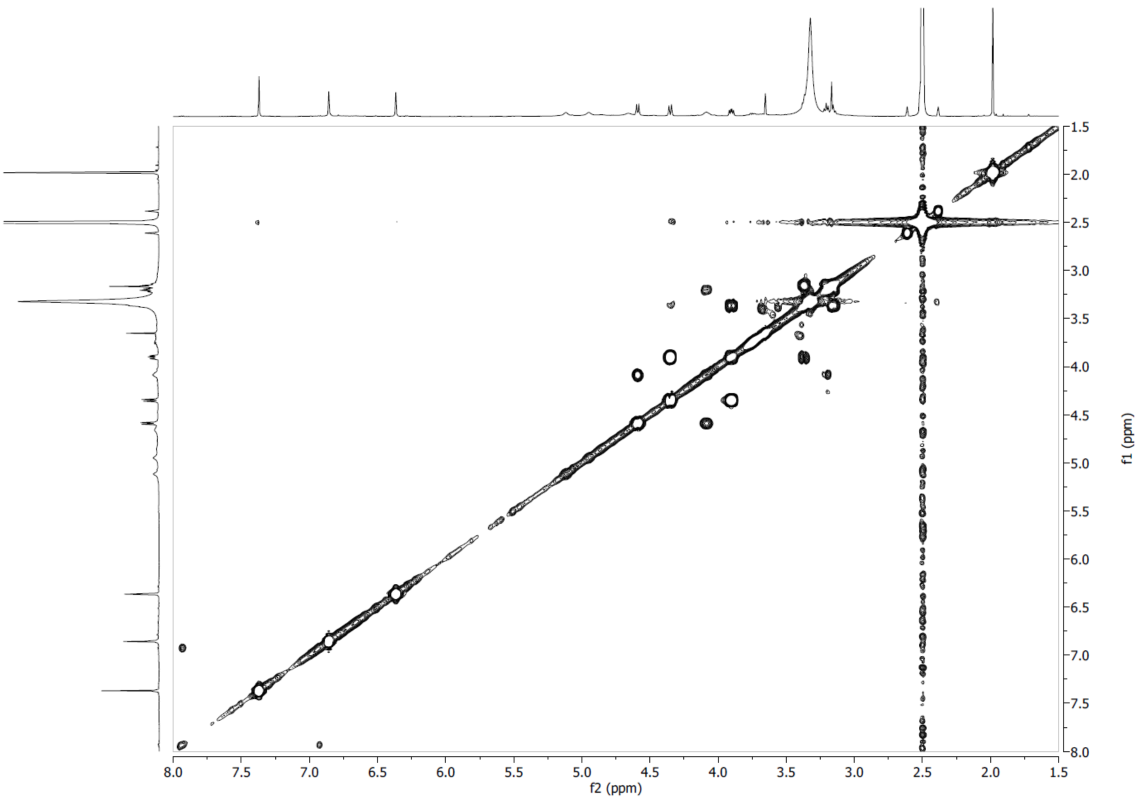

Supplementary Figure 2.66. COSY NMR spectrum of 6'-*O*-acetylmangiferin (**8**) in DMSO-d_6

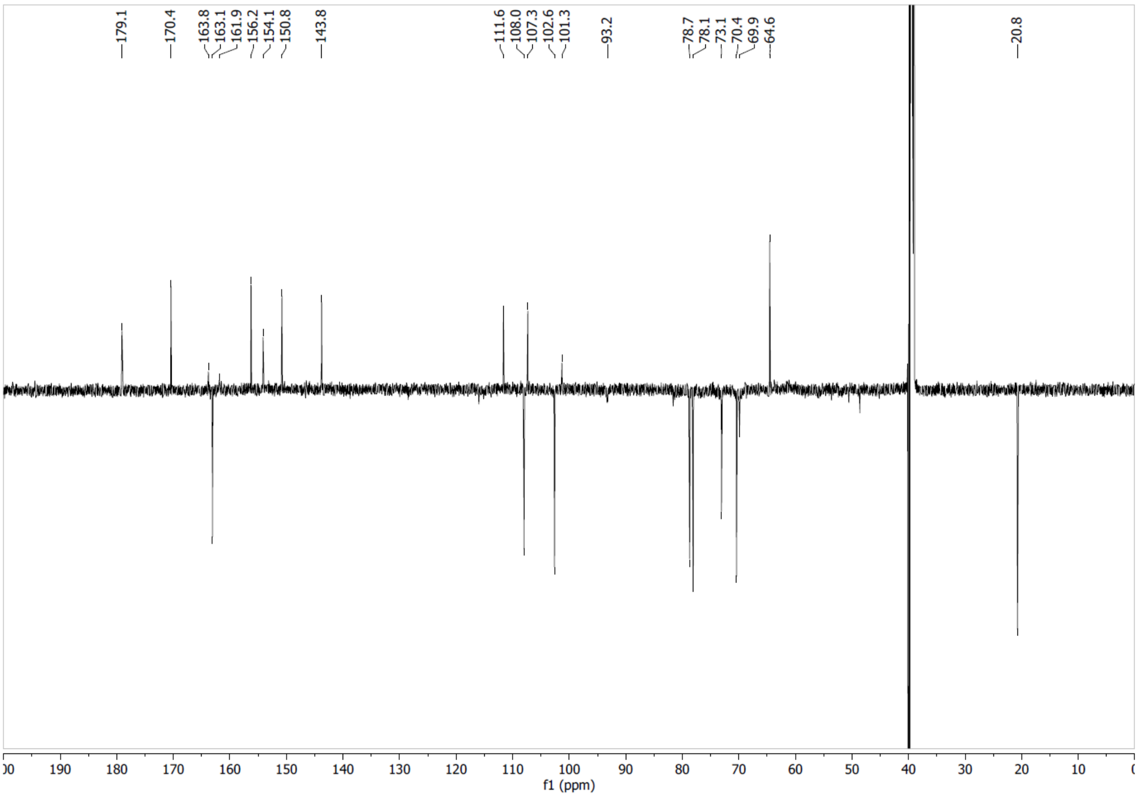

Supplementary Figure 2. 67. ^{13}C-DEPTQ NMR spectrum of 6'-*O*-acetylmangiferin (**8**) in DMSO-d_6 at 600 MHz

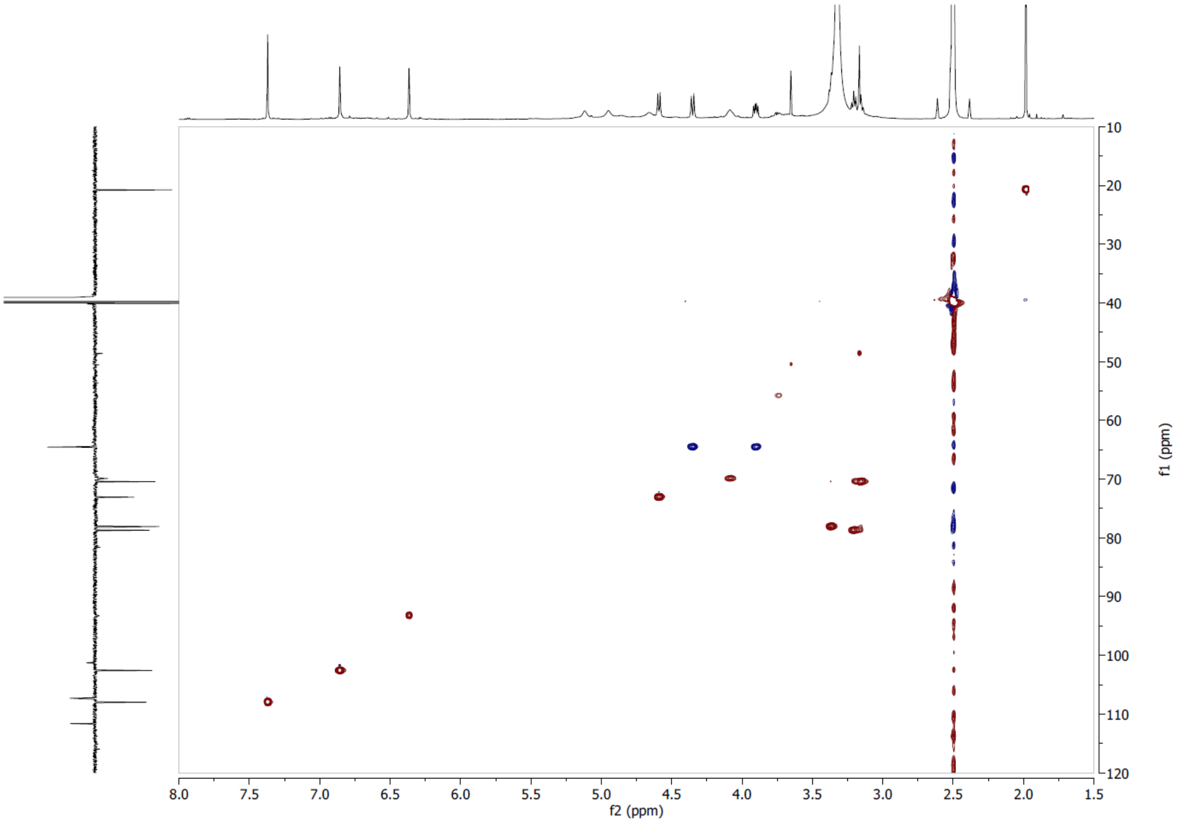

Supplementary Figure 2.68. Edited-HSQC NMR spectrum of 6'-*O*-acetylmangiferin (**8**) in DMSO-d_6

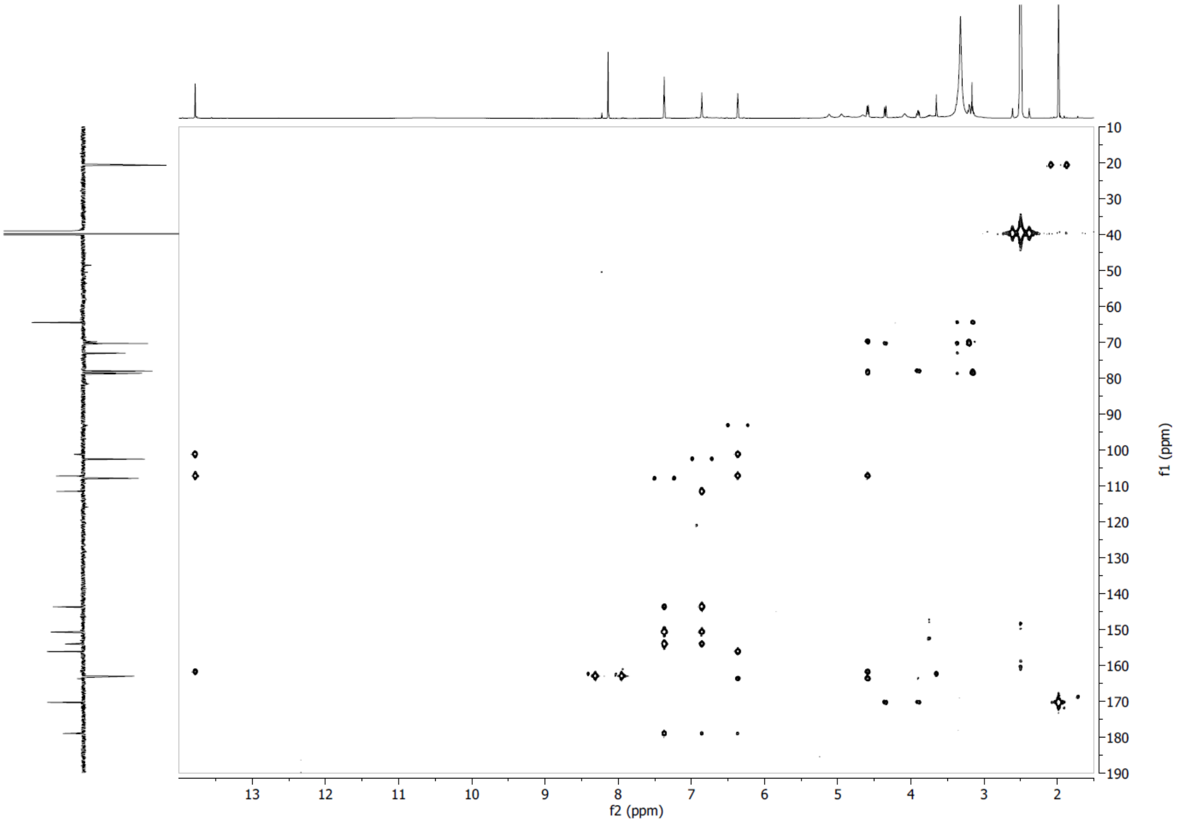

Supplementary Figure 2.69. HMBC NMR spectrum of 6'-*O*-acetylmangiferin (**8**) in DMSO-d_6

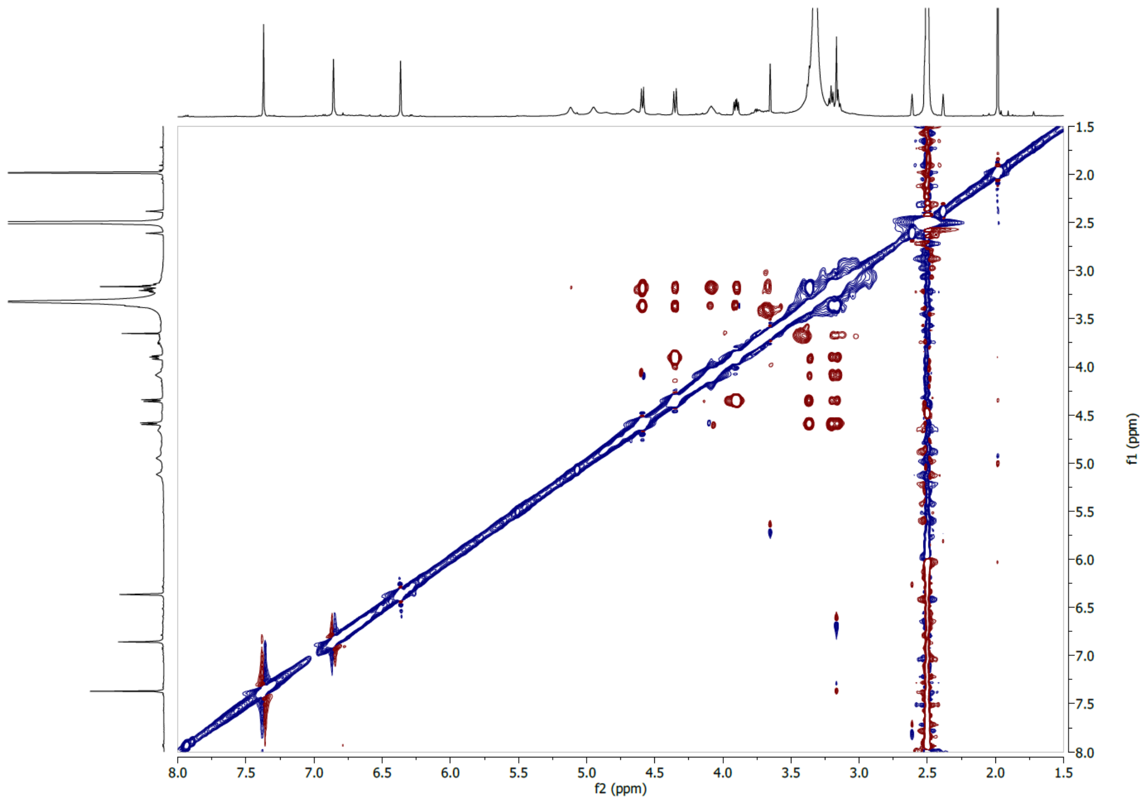

Supplementary Figure 2.70. ROESY NMR spectrum of 6'-*O*-acetylmangiferin (**8**) in DMSO-d_6

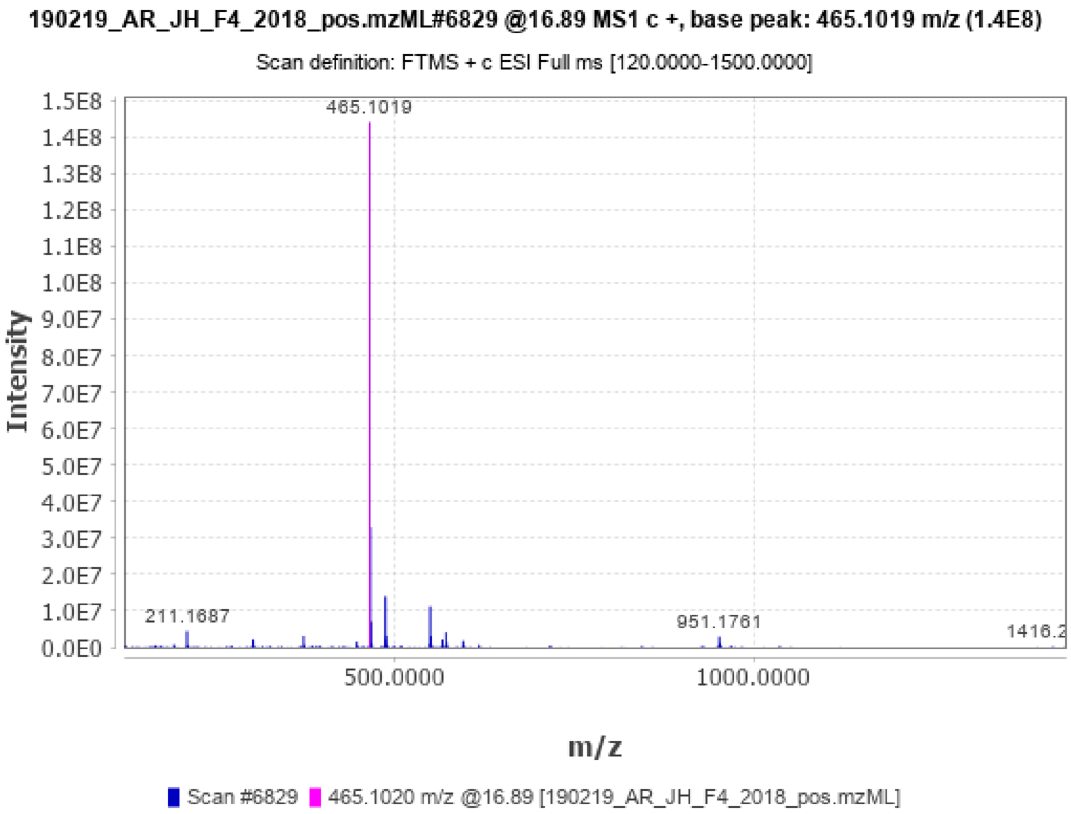

Supplementary Figure 2.71. HRMS spectrum of 6'-*O*-acetylmangiferin (**8**) ([M+H]$^+$) in Fraction 4 obtained by UHPLC-HRMS in positive ionization.

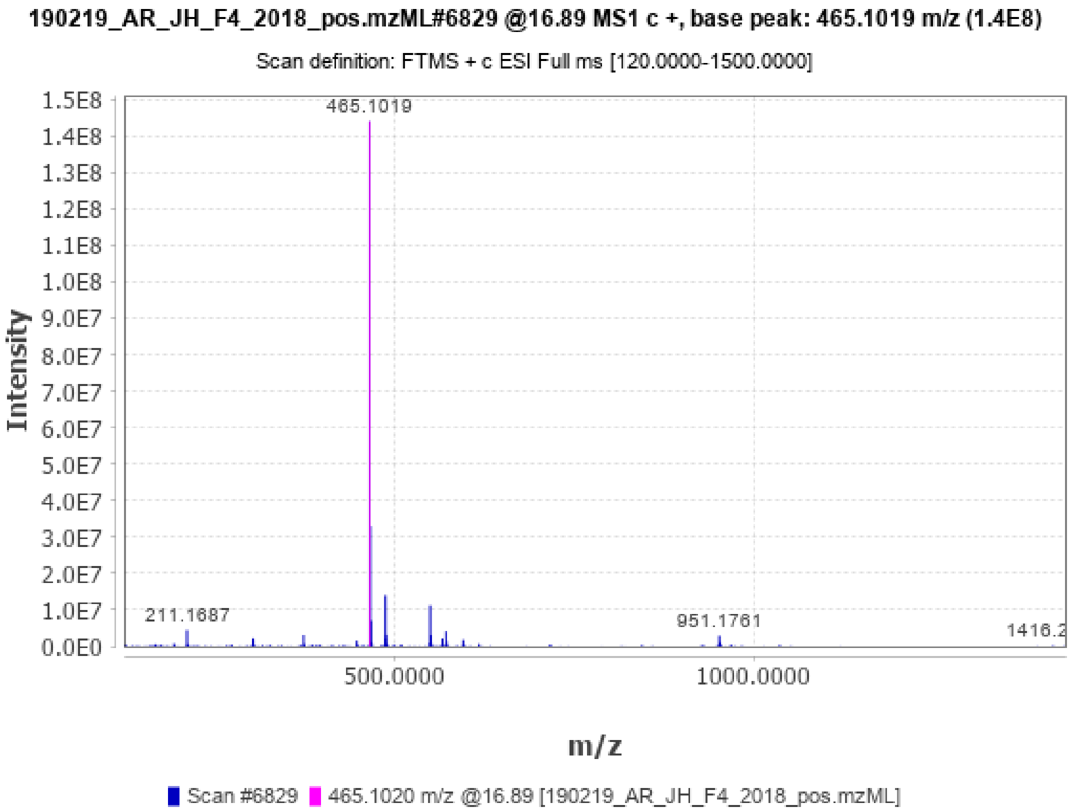

Supplementary Figure 2.72. Fragmentation spectrum of 6'-*O*-acetylmangiferin (**8**) ([M+H]$^+$) in Fraction 4 obtained by UHPLC-HRMS in positive ionization.

9. Genkwanin 5-*O*-β-primeveroside (9)

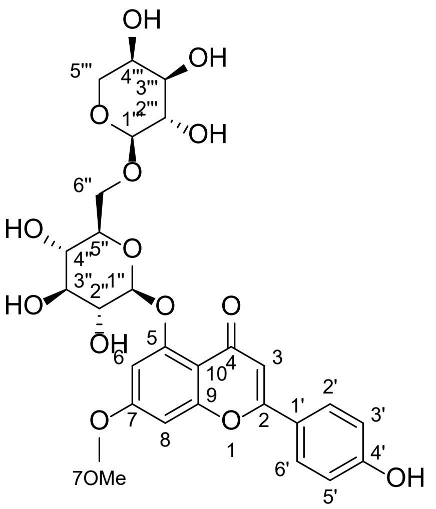

Chemical Formula: $C_{27}H_{30}O_{14}$
Exact Mass: 578.16

Supplementary Figure 2.73. Structure of Genkwanin 5-*O*-β-primeveroside (9)

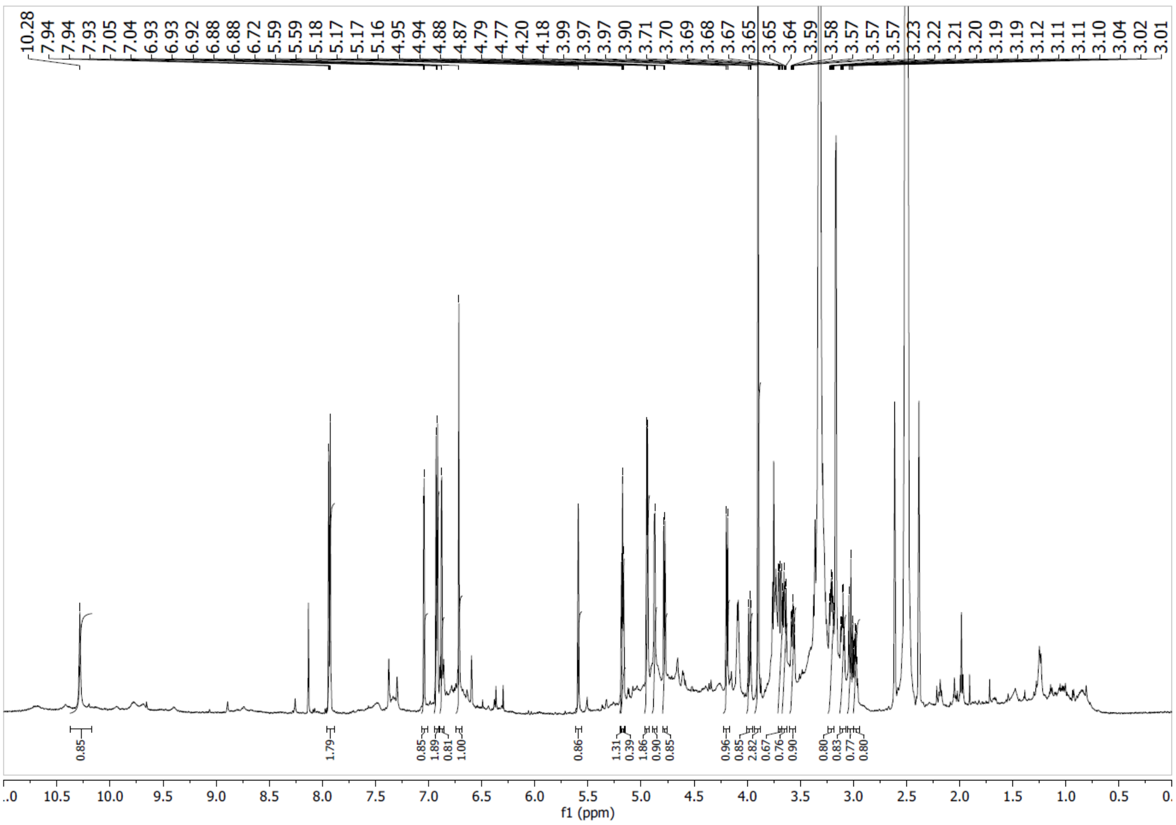

Supplementary Figure 2.74. ^{1}H NMR spectrum of genkwanin 5-*O*-β-primeveroside (**9**) in DMSO-d_6 at 600 MHz

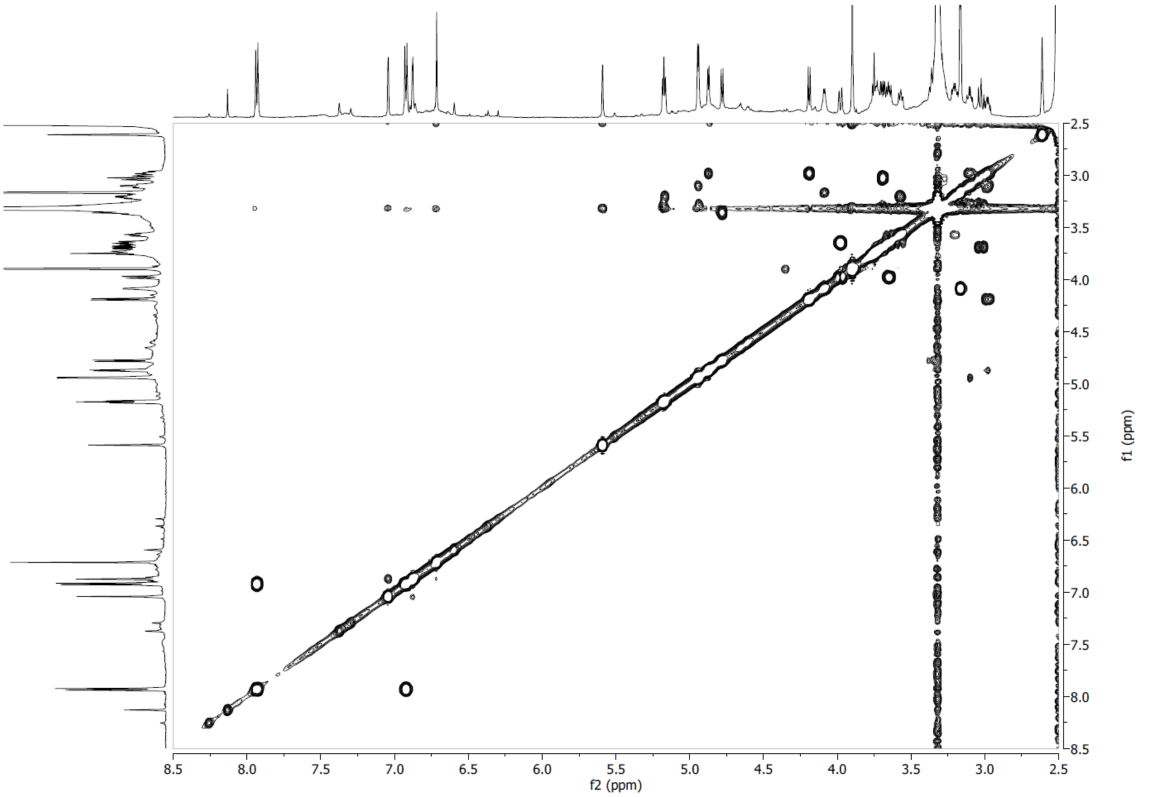

Supplementary Figure 2.75. COSY NMR spectrum of genkwanin 5-*O*-β-primeveroside (**9**) in DMSO-d_6

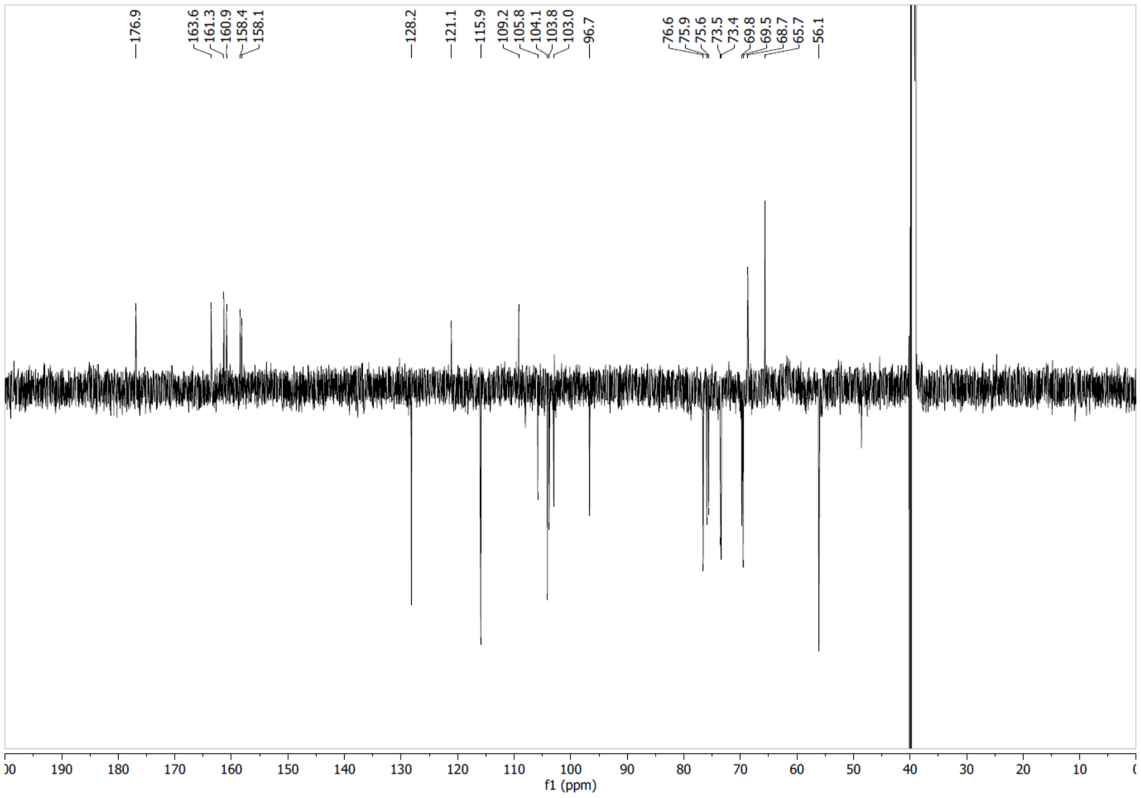

Supplementary Figure 2.76. ^{13}C-DEPTQ NMR spectrum of genkwanin 5-*O*-β-primeveroside (**9**) in DMSO-d_6 at 151 MHz

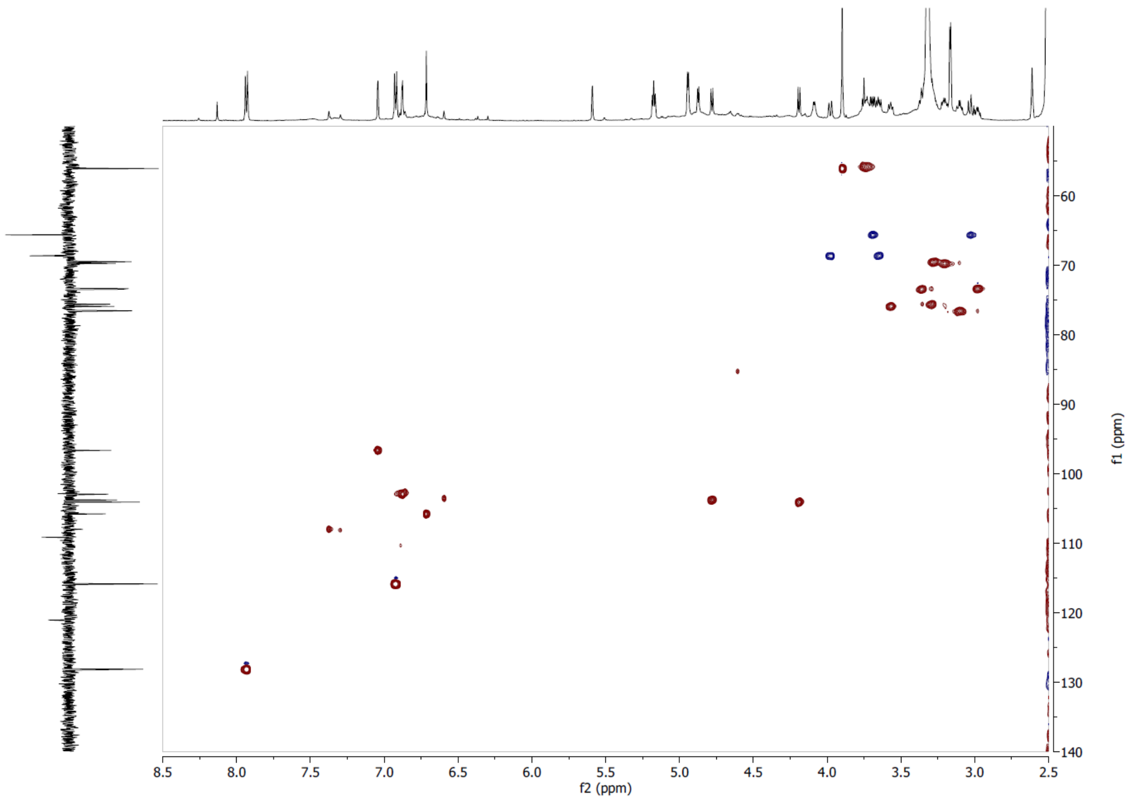

Supplementary Figure 2.77. Edited-HSQC NMR spectrum of genkwanin 5-*O*-β-primeveroside (**9**) in DMSO-*d*6

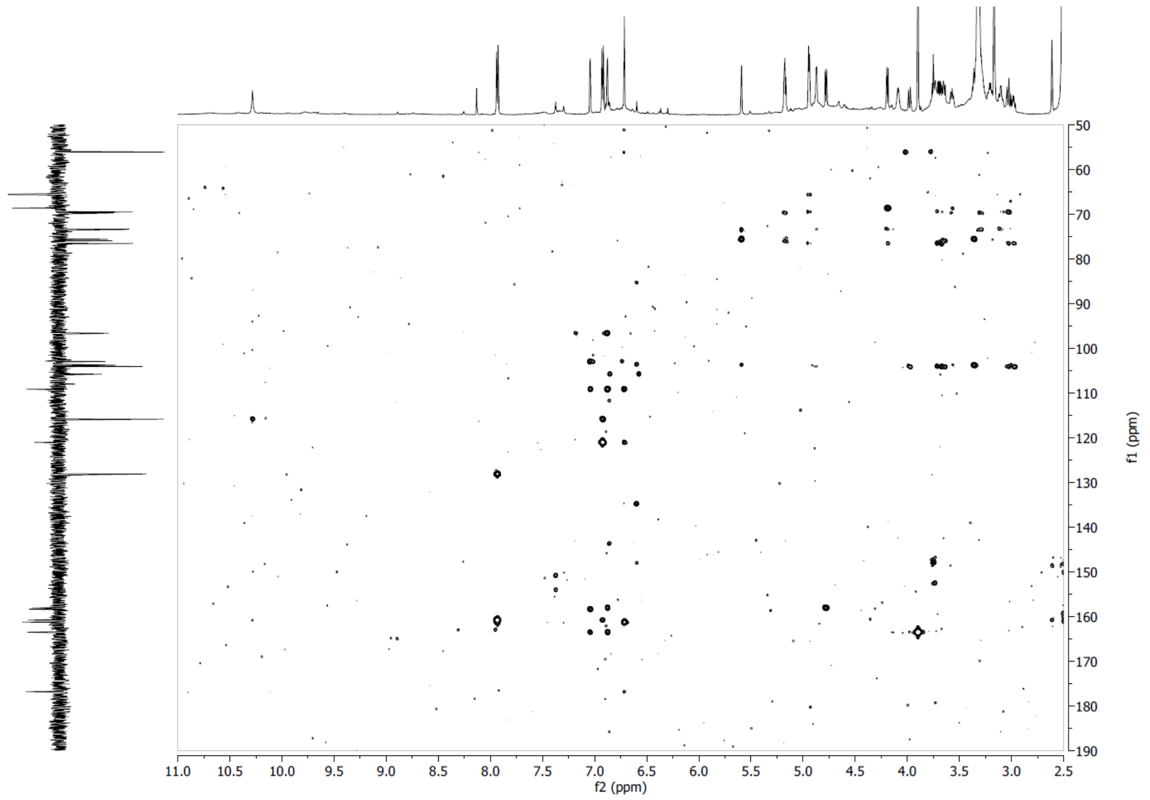

Supplementary Figure 2.78. HMBC NMR spectrum of genkwanin 5-*O*-β-primeveroside (**9**) in DMSO-*d*6

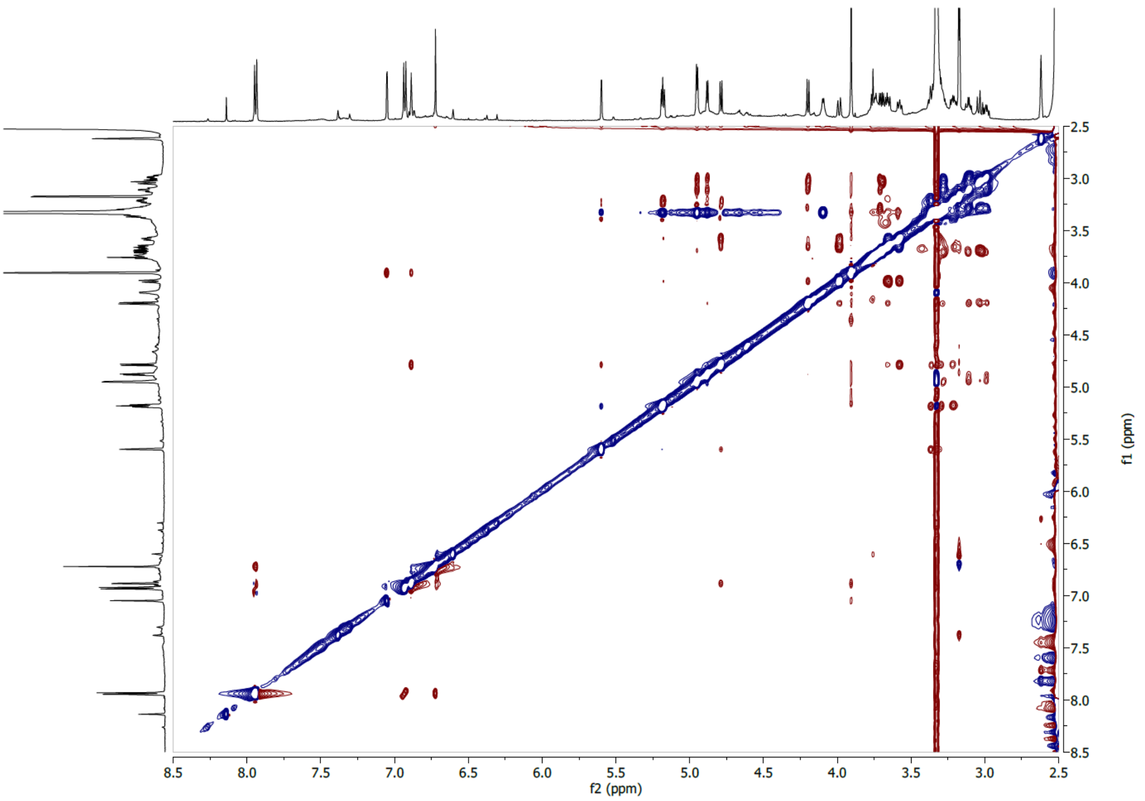

Supplementary Figure 2.79. ROESY NMR spectrum of genkwanin 5-*O*-β-primeveroside (**9**) in DMSO-d_6

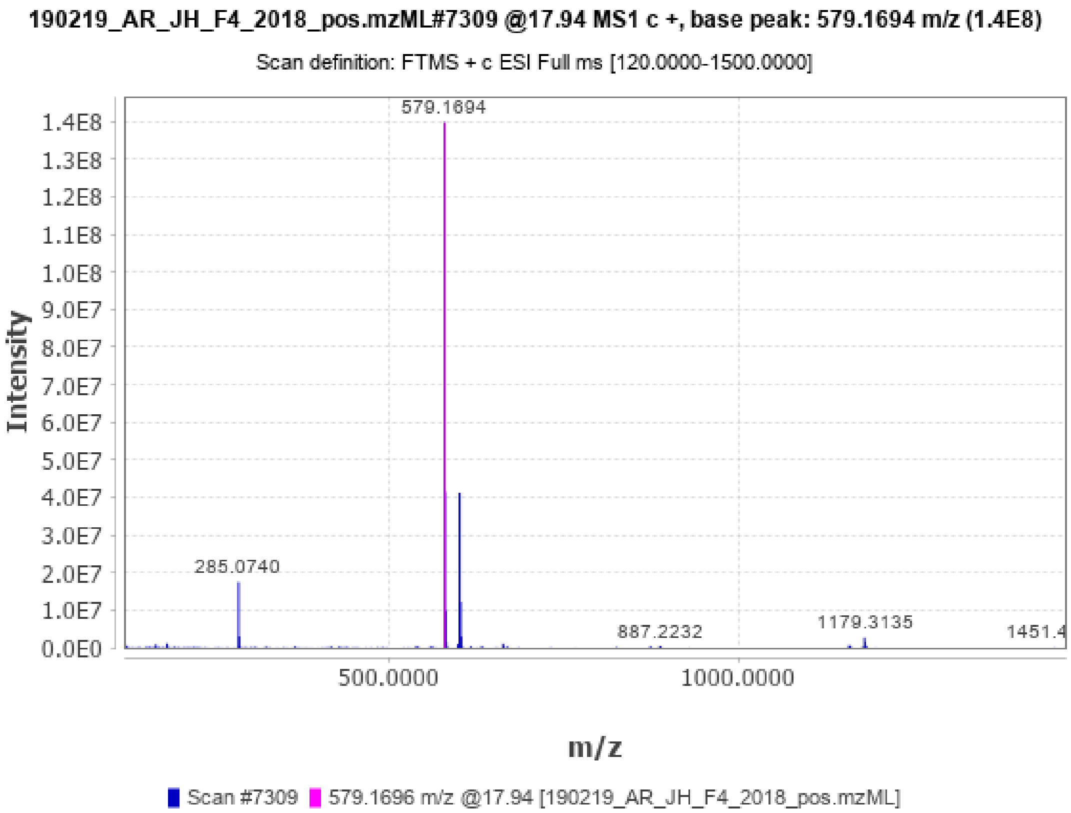

Supplementary Figure 2.80. HRMS spectrum of genkwanin 5-*O*-β-primeveroside (**9**) ([M+H]$^+$) in Fraction 4 obtained by UHPLC-HRMS in positive ionization.

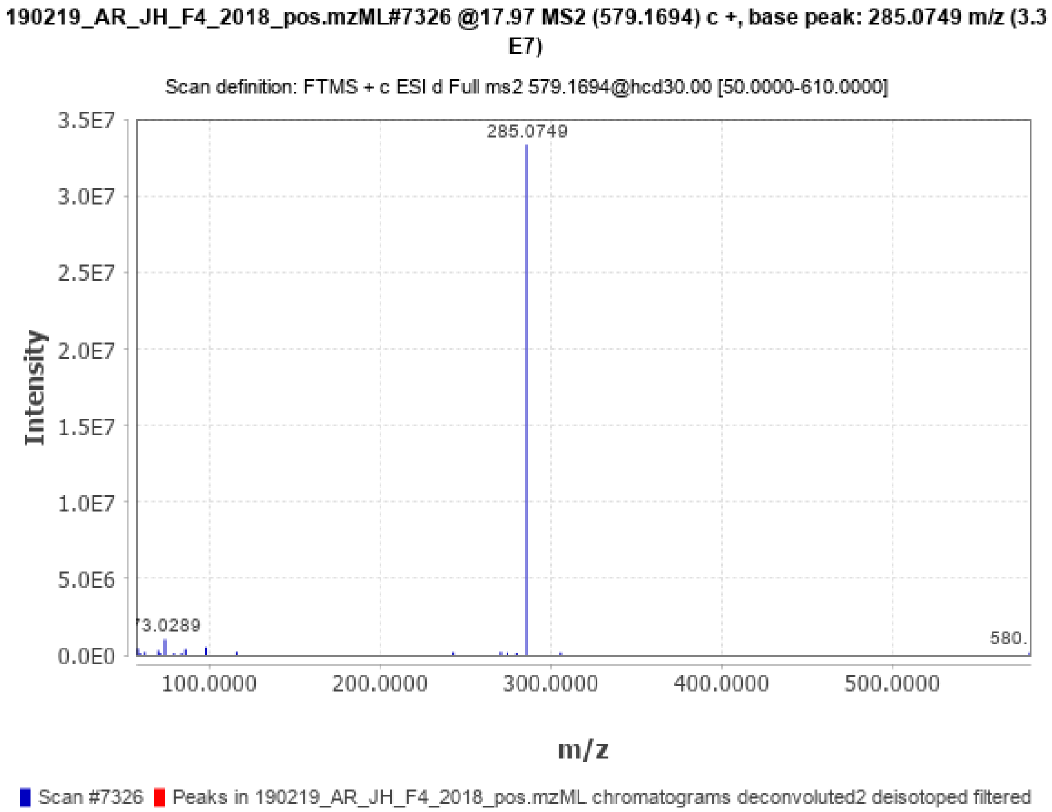

Supplementary Figure 2.81. Fragmentation spectrum of genkwanin 5-*O*-β-primeveroside (**9**) ([M+H]$^+$) in Fraction 4 obtained by UHPLC-HRMS in positive ionization.

10. Genkwanin 5-*O*-β-glucoside (10)

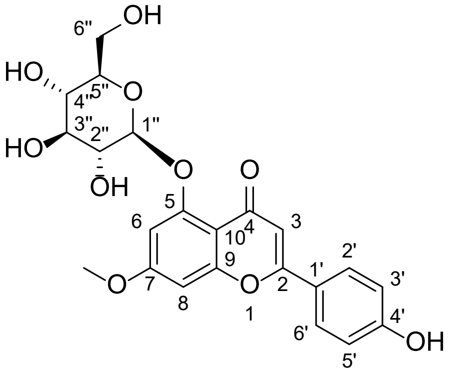

Chemical Formula: $C_{22}H_{22}O_{10}$
Exact Mass: 446.12

Supplementary Figure 2.82. Structure of Genkwanin 5-*O*-β-glucoside (**10**)

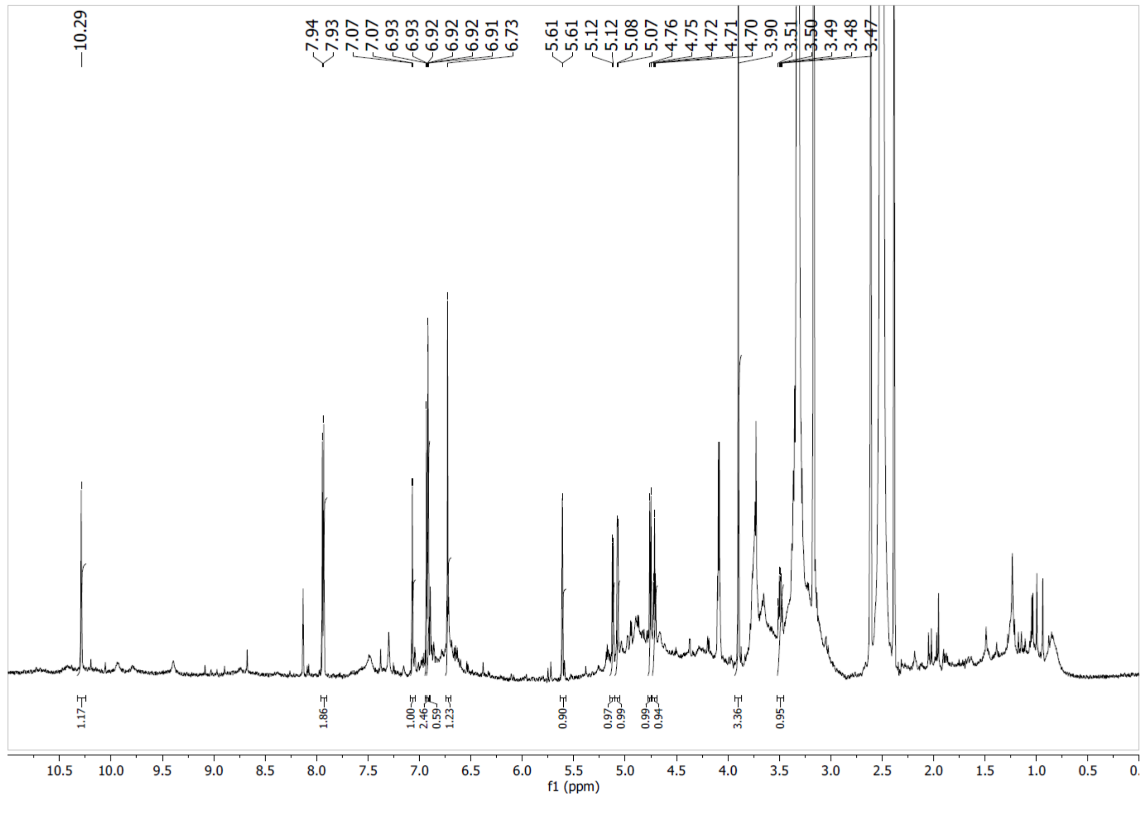

Supplementary Figure 2.83. ^{1}H NMR spectrum of genkwanin 5-*O*-β-glucoside (**10**) in DMSO-d_6 at 600 MHz

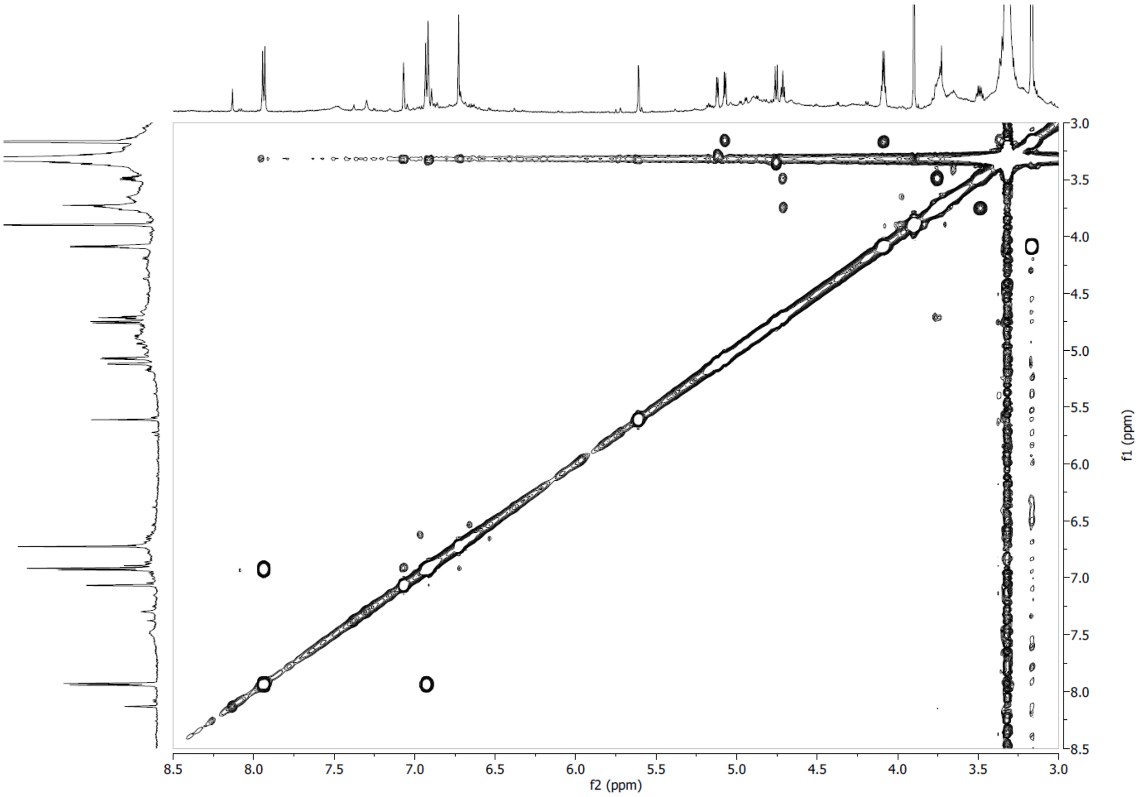

Supplementary Figure 2.84. COSY NMR spectrum of genkwanin 5-*O*-β-glucoside (**10**) in DMSO-d_6

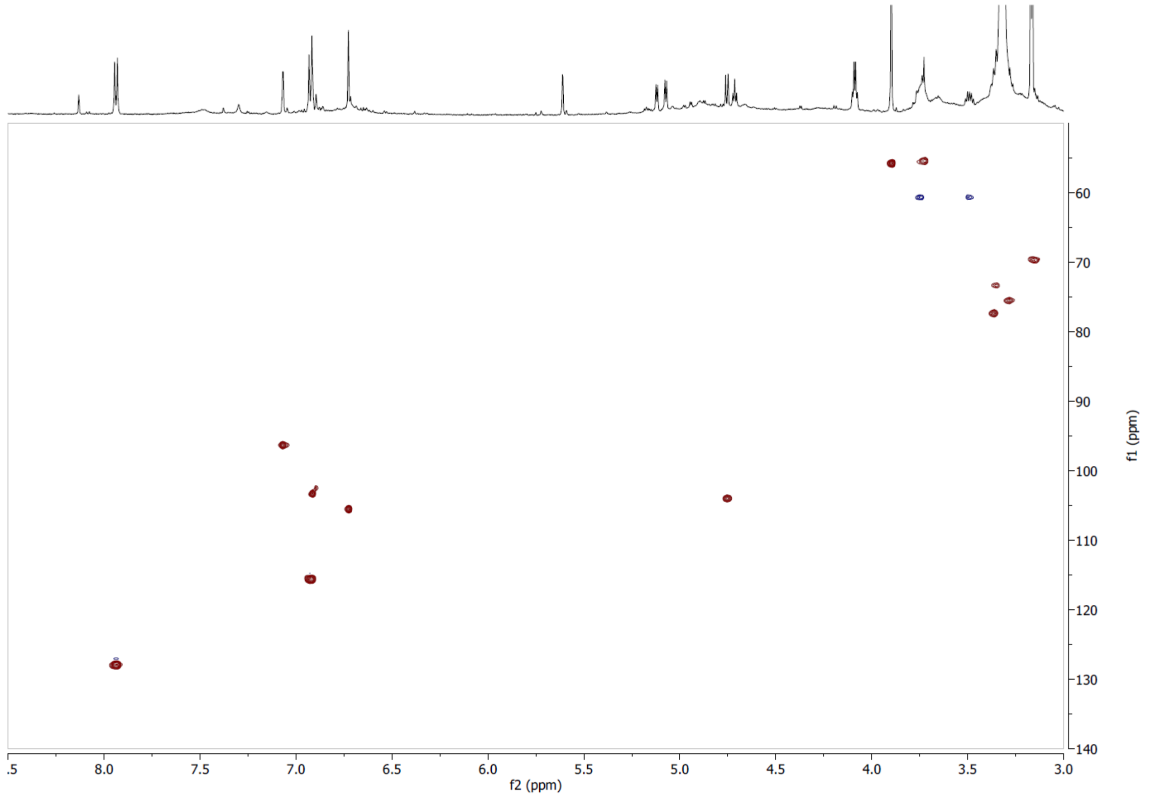

Supplementary Figure 2.85. Edited-HSQC NMR spectrum of genkwanin 5-*O*-β-glucoside (**10**) in DMSO-d_6

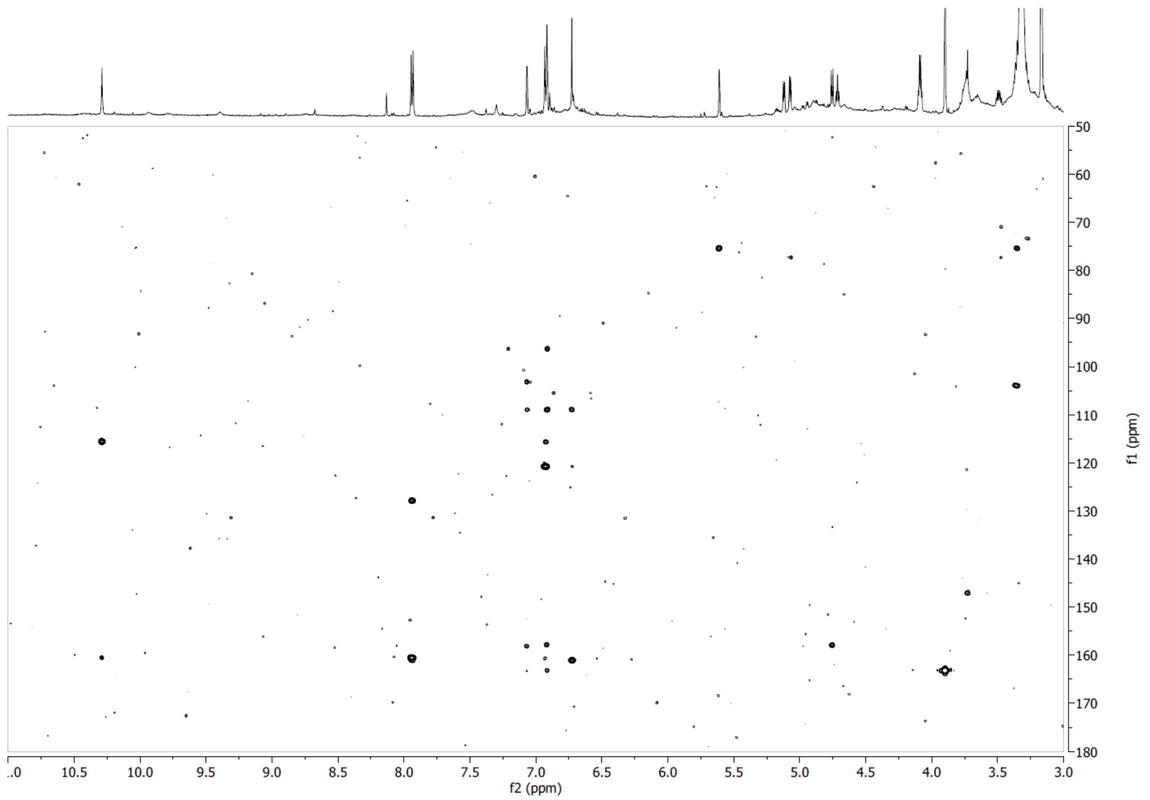

Supplementary Figure 2.86. HMBC NMR spectrum of genkwanin 5-*O*-β-glucoside (**10**) in DMSO-d_6

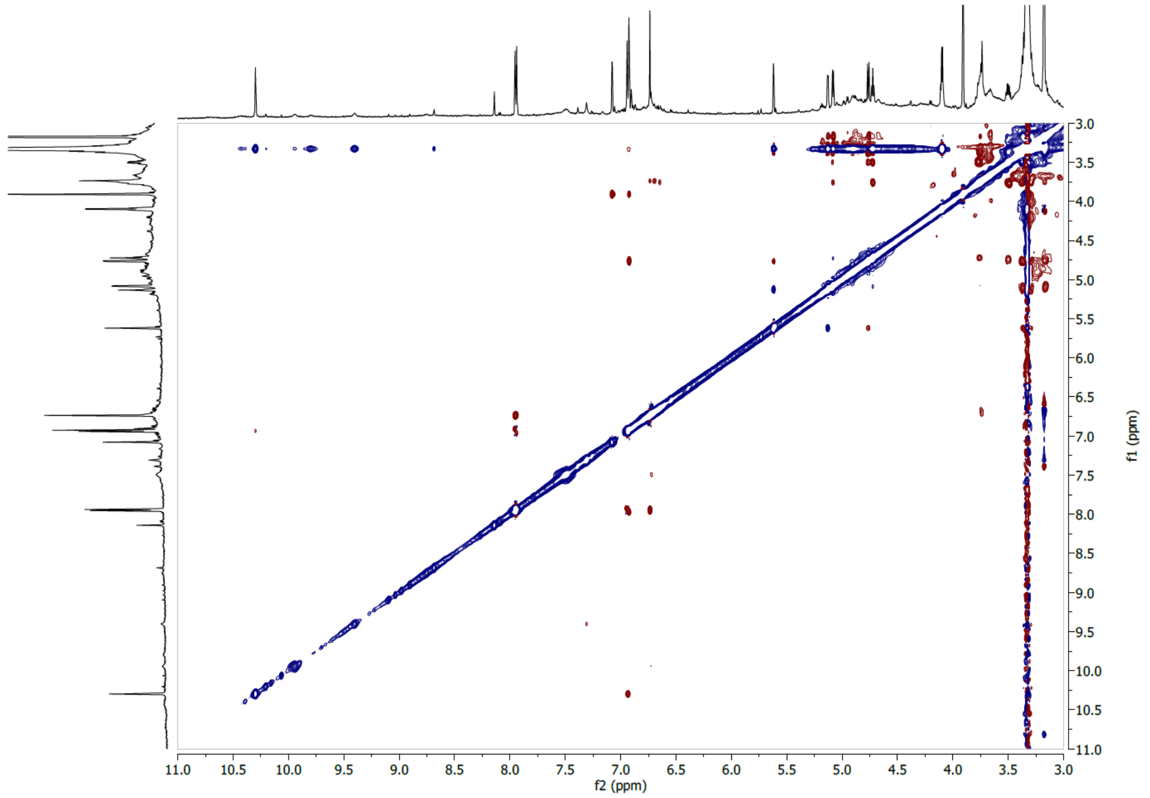

Supplementary Figure 2.87. ROESY NMR spectrum of genkwanin 5-*O*-β-glucoside (**10**) in DMSO-d_6

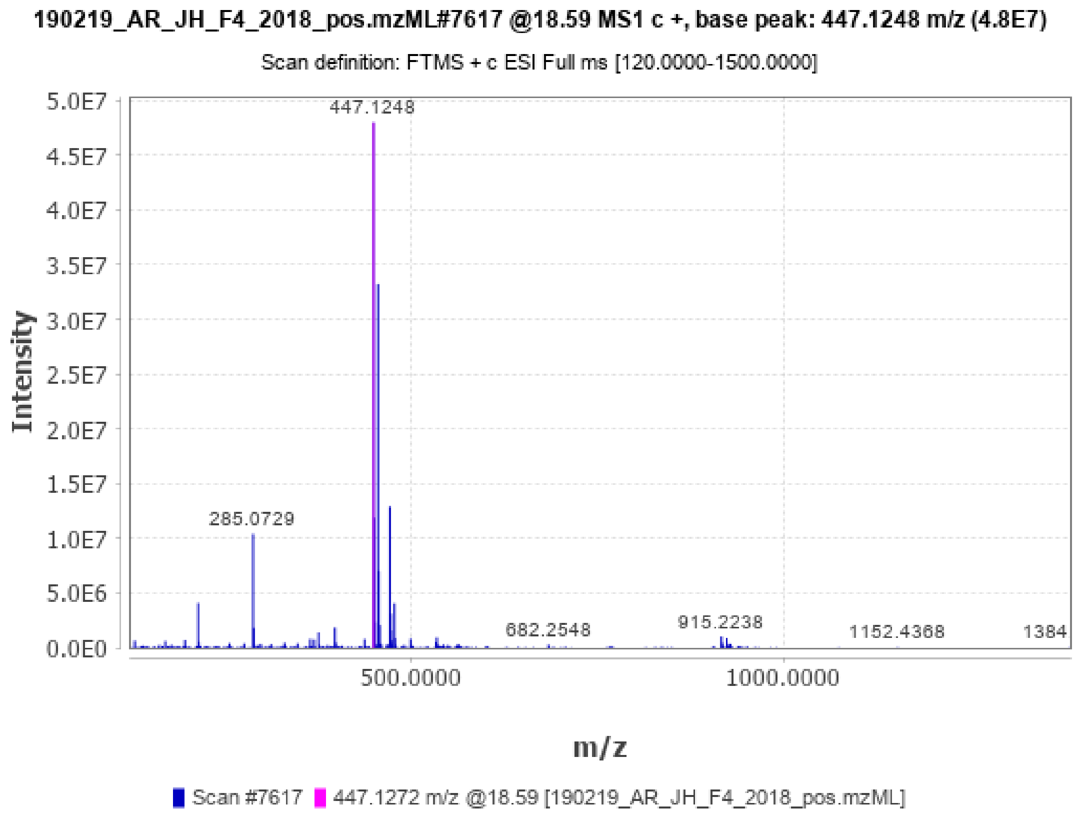

Supplementary Figure 2.88. HRMS spectrum of genkwanin 5-*O*-β-glucoside (**10**) ($[M+H]^+$) in Fraction 4 obtained by UHPLC-HRMS in positive ionization.

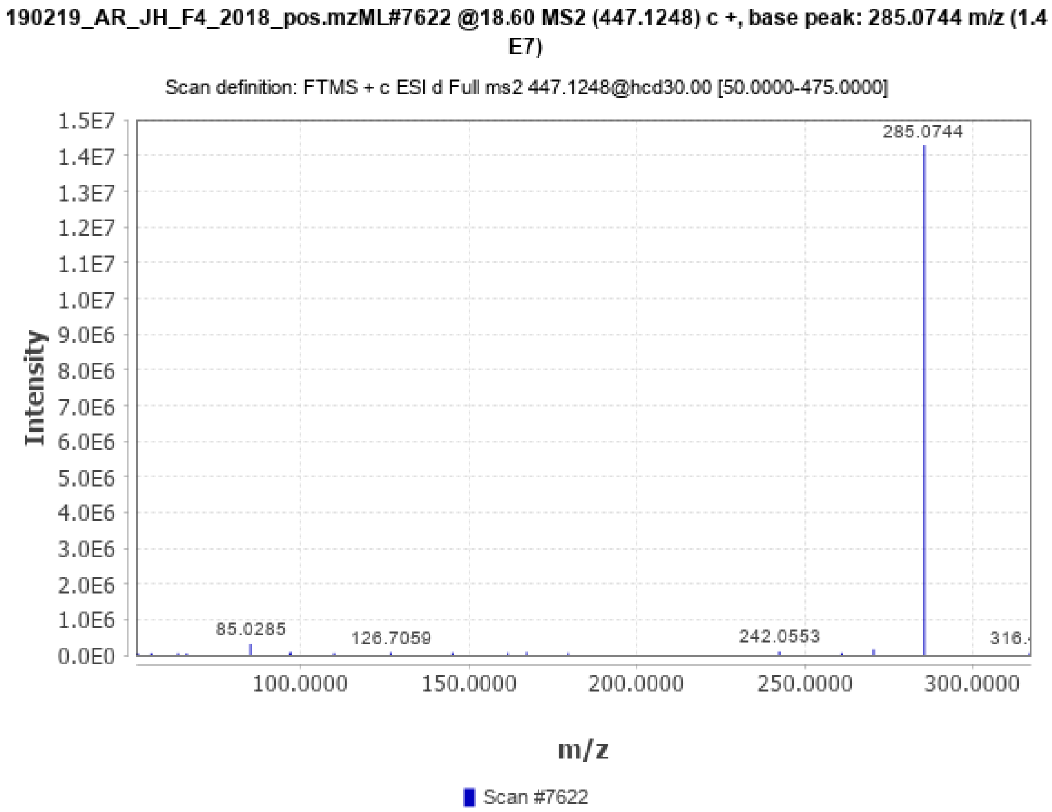

Supplementary Figure 2.89. Fragmentation spectrum of genkwanin 5-*O*-β-glucoside (**10**) ($[M+H]^+$) in Fraction 4 obtained by UHPLC-HRMS in positive ionization.
